# Global burden of acute lower respiratory infection associated with human metapneumovirus in children under 5 years in 2018: a systematic review and modelling study

**DOI:** 10.1016/S2214-109X(20)30393-4

**Published:** 2020-11-26

**Authors:** Xin Wang, You Li, Maria Deloria-Knoll, Shabir A Madhi, Cheryl Cohen, Asad Ali, Sudha Basnet, Quique Bassat, W Abdullah Brooks, Malinee Chittaganpitch, Marcela Echavarria, Rodrigo A Fasce, Doli Goswami, Siddhivinayak Hirve, Nusrat Homaira, Stephen R C Howie, Karen L Kotloff, Najwa Khuri-Bulos, Anand Krishnan, Marilla G Lucero, Socorro Lupisan, Ainara Mira-Iglesias, David P Moore, Cinta Moraleda, Marta Nunes, Histoshi Oshitani, Betty E Owor, Fernando P Polack, Katherine L O'Brien, Zeba A Rasmussen, Barbara A Rath, Vahid Salimi, J Anthony G Scott, Eric A F Simões, Tor A Strand, Donald M Thea, Florette K Treurnicht, Linda C Vaccari, Lay-Myint Yoshida, Heather J Zar, Harry Campbell, Harish Nair, Respiratory Virus Global Epidemiology Network, Romina Libster, Grieven Otieno, Imane Joundi, Shobha Broor, Mark Nicol, Ritvik Amarchand, Ting Shi, F. Xavier López-Labrador, Julia M. Baker, Alexandra Jamison, Avinash Choudekar, Sanjay Juvekar, Patrick Obermeier, Brunhilde Schweiger, Lola Madrid, Elizabeth Thomas, Miguel Lanaspa, Hanna Nohynek, James Nokes, Marta Werner, Anh Danhg, Mandeep Chadha, Joan Puig-Barberà, Mauricio T. Caballero, Maria Mathisen, Sibongile Walaza, Orienka Hellferscee, Matt Laubscher, Melissa M. Higdon, Meredith Haddix, Pongpun Sawatwong, Henry C. Baggett, Phil Seidenberg, Lawrence Mwanayanda, Martin Antonio, Bernard E. Ebruke, Tanja Adams, Mustafizur Rahman, Mohammed Ziaur Rahman, Samboa O. Sow, Vicky L. Baillie, Lesley Workman, Michiko Toizumi, Milagritos D. Tapia, Thi hien anh Nguyen, Susan Morpeth

**Affiliations:** a Centre for Global Health, Usher Institute, Edinburgh Medical School, University of Edinburgh, Edinburgh, UK; b Department of International Health, International Vaccine Access Center, Johns Hopkins Bloomberg School of Public Health, Baltimore, MD, USA; c Medical Research Council: Respiratory and Meningeal Pathogens Research Unit, Faculty of Health Sciences, University of the Witwatersrand, Johannesburg, South Africa; d Department of Science and Technology/National Research Foundation: Vaccine Preventable Diseases, Faculty of Health Sciences, University of the Witwatersrand, Johannesburg, South Africa; e Department of Paediatrics and Child Health, Chris Hani Baragwanath Academic Hospital, Faculty of Health Sciences, University of the Witwatersrand, Johannesburg, South Africa; f School of Public Health, Faculty of Health Sciences, University of the Witwatersrand, Johannesburg, South Africa; g Centre for Respiratory Disease and Meningitis, National Institute for Communicable Diseases, Johannesburg, South Africa; h Department of Pediatrics and Child Health, Aga Khan University, Karachi, Pakistan; i Department of Child Health, Tribhuvan University, Kathmandu, Nepal; j Centre for International Health, University of Bergen, Bergen, Norway; k Barcelona Global Health Institute, Hospital Clínic–University of Barcelona, Barcelona, Spain; l Centro de Investigação em Saúde de Manhiça, Maputo, Mozambique; m Institució Catalana de Recerca i Estudis Avançats, Barcelona, Spain; n Pediatric Infectious Diseases Unit, Pediatrics Department, Hospital Sant Joan de Déu (University of Barcelona), Barcelona, Spain; o Consorcio de Investigación Biomédica en Red de Epidemiología y Salud Pública, Madrid, Spain; p Medical Sciences Technical Office, Department of Medical Sciences, Ministry of Public Health, Nonthaburi, Thailand; q Clinical Virology Unit, Centro de Educación Médica e Investigaciones Clínicas, Buenos Aires, Argentina; r Public Health Institute of Chile, Santiago, Chile; s International Centre for Diarrhoeal Disease Research Bangladesh, Dhaka, Bangladesh; t KEM Hospital Research Centre, Pune, India; u Discipline of Paediatrics, School of Women's and Children's Health, The University of New South Wales, Sydney, NSW, Australia; v Medical Research Council Unit The Gambia at London School of Hygiene & Tropical Medicine, Banjul, The Gambia; w Department of Paediatrics: Child and Youth Health, University of Auckland, Auckland, New Zealand; x Department of Pediatrics and Department of Medicine, Center for Vaccine Development and Global Health, University of Maryland School of Medicine, Baltimore, MD, USA; y Department of Pediatrics, University of Jordan School of Medicine, Amman, Jordan; z Centre for Community Medicine, All India Institute of Medical Sciences, New Delhi, India; aa Research Institute for Tropical Medicine, Muntinlupa, Philippines; ab Área de Investigación en Vacunas, Fundación para el Fomento de la Investigación Sanitaria y Biomédica de la Comunitat Valenciana (Salud Pública), Valencia, Spain; ac Infectious Pediatric Diseases Section, Hospital Universitario de Octubre, Universidad Complutense, Research Institute Hospital de Octubre, Madrid, Spain; ad Department of Virology, Tohoku University Graduate School of Medicine, Sendai, Japan; ae KEMRI–Wellcome Trust Research Programme, Kilifi, Kenya; af Fundacion INFANT, Buenos Aires, Argentina; ag Fogarty International Center, National Institutes of Health, Bethesda, MD, USA; ah Vienna Vaccine Safety Initiative, Berlin, Germany; ai Department of Virology, School of Public Health, Tehran University of Medical Sciences, Tehran, Iran; aj Nuffield Department of Tropical Medicine, Oxford University, Oxford, UK; ak Department of Infectious Disease Epidemiology, London School of Hygiene & Tropical Medicine, London, UK; al Department of Pediatrics, School of Medicine, and Department of Epidemiology and Center for Global Health, Colorado School of Public Health, University of Colorado, Aurora, CO, USA; am Innland Hosptial Trust, Lillehammer, Norway; an Department of Global Health and Development, Boston University School of Public Health, Boston, MA, USA; ao Department of Pediatric Infectious Diseases, Institute of Tropical Medicine, Nagasaki University, Nagasaki, Japan; ap Department of Paediatrics and Child Health and Medical Research Council Unit on Child and Adolescent Health, University of Cape Town, Cape Town, South Africa

## Abstract

**Background:**

Human metapneumovirus is a common virus associated with acute lower respiratory infections (ALRIs) in children. No global burden estimates are available for ALRIs associated with human metapneumovirus in children, and no licensed vaccines or drugs exist for human metapneumovirus infections. We aimed to estimate the age-stratified human metapneumovirus-associated ALRI global incidence, hospital admissions, and mortality burden in children younger than 5 years.

**Methods:**

We estimated the global burden of human metapneumovirus-associated ALRIs in children younger than 5 years from a systematic review of 119 studies published between Jan 1, 2001, and Dec 31, 2019, and a further 40 high quality unpublished studies. We assessed risk of bias using a modified Newcastle-Ottawa Scale. We estimated incidence, hospital admission rates, and in-hospital case-fatality ratios (hCFRs) of human metapneumovirus-associated ALRI using a generalised linear mixed model. We applied incidence and hospital admission rates of human metapneumovirus–associated ALRI to population estimates to yield the morbidity burden estimates by age bands and World Bank income levels. We also estimated human metapneumovirus-associated ALRI in-hospital deaths and overall human metapneumovirus-associated ALRI deaths (both in-hospital and non-hospital deaths). Additionally, we estimated human metapneumovirus-attributable ALRI cases, hospital admissions, and deaths by combining human metapneumovirus-associated burden estimates and attributable fractions of human metapneumovirus in laboratory-confirmed human metapneumovirus cases and deaths.

**Findings:**

In 2018, among children younger than 5 years globally, there were an estimated 14·2 million human metapneumovirus-associated ALRI cases (uncertainty range [UR] 10·2 million to 20·1 million), 643 000 human metapneumovirus-associated hospital admissions (UR 425 000 to 977 000), 7700 human metapneumovirus-associated in-hospital deaths (2600 to 48 800), and 16 100 overall (hospital and community) human metapneumovirus-associated ALRI deaths (5700 to 88 000). An estimated 11·1 million ALRI cases (UR 8·0 million to 15·7 million), 502 000 ALRI hospital admissions (UR 332 000 to 762 000), and 11 300 ALRI deaths (4000 to 61 600) could be causally attributed to human metapneumovirus in 2018. Around 58% of the hospital admissions were in infants under 12 months, and 64% of in-hospital deaths occurred in infants younger than 6 months, of which 79% occurred in low-income and lower-middle-income countries.

**Interpretation:**

Infants younger than 1 year have disproportionately high risks of severe human metapneumovirus infections across all World Bank income regions and all child mortality settings, similar to respiratory syncytial virus and influenza virus. Infants younger than 6 months in low-income and lower-middle-income countries are at greater risk of death from human metapneumovirus-associated ALRI than older children and those in upper-middle-income and high-income countries. Our mortality estimates demonstrate the importance of intervention strategies for infants across all settings, and warrant continued efforts to improve the outcome of human metapneumovirus-associated ALRI among young infants in low-income and lower-middle-income countries.

**Funding:**

Bill & Melinda Gates Foundation.

## Introduction

Acute lower respiratory infections (ALRIs) are one of the leading causes of morbidity and mortality in children globally, accounting for 10% of deaths in children younger than 5 years in 2017.^1^ Human metapneumovirus, first identified in 2001, is an important virus that causes ALRIs in young children.^2^, ^3^, ^4^, ^5^ Previous evidence indicates that most children are infected with human metapneumovirus by age 5 years, with most severe infections occurring in infants, including symptomatic and asymptomatic infections.^6^, ^7^, ^8^, ^9^, ^10^ Pooled analyses have shown that human metapneumovirus is associated with 6·1–6·4% of hospital admissions due to ALRI among patients younger than 20 years worldwide.^11^, ^12^ Few studies have reported the incidence and mortality for human metapneumovirus-associated ALRIs, and studies have rarely reported disease burden in narrower age groups (eg, those younger than 6 months). No global or regional burden estimates are available for children under 5 years.

**Research in context**

**Evidence before this study**

Human metapneumovirus is a common virus in children with acute lower respiratory infection (ALRI). Two pooled analyses have shown that human metapneumoviruses are identified in 6·1–6·4% of ALRIs in patients younger than 20 years globally. However, no global estimates of human metapneumovirus-associated ALRI burden are available for children younger than 5 years. The estimation of global human metapneumovirus burden is challenging due to the paucity of population-based studies on the incidence of human metapneumovirus infections.

**Added value of this study**

We did a meta-analysis based on published and unpublished data on laboratory-confirmed human metapneumovirus-associated ALRI incidence, hospital admissions, and mortality burden. We included 159 studies with data on human metapneumovirus-associated ALRI community incidence, hospital admission rates, proportion of human metapneumovirus-positive cases among hospitalised ALRI cases, and in-hospital case-fatality ratios, which included 40 high quality unpublished studies that provided data stratified by narrow age groups in children younger than 5 years. 27% of the studies were from low-income and lower-middle-income countries. Our estimates of human metapneumovirus-associated ALRI burden were stratified by the level of child mortality of the setting and by age groups, from which we identified the population subgroups most susceptible to human metapneumovirus-associated ALRI morbidity and mortality, identifying target populations and settings for future intervention studies and informing intervention strategies. Since the presence of human metapneumovirus in the upper respiratory tract in children with ALRIs does not indicate causation, we reported the global burden of ALRIs that are causally attributable to human metapneumovirus (human metapneumovirus-attributable ALRI burden), which could aid understanding with regard to the involvement of this virus in causing childhood ALRI. We estimated that human metapneumovirus could be identified in 11% of ALRI cases, 4–13% of hospital admissions due to ALRIs, and 2% of ALRI deaths among children under 5 years globally, using our estimates in conjunction with previously published all-cause ALRI burden estimates. After accounting for the causal attribution of human metapneumovirus, we estimated that 8% of ALRI cases, 3–10% of hospital admissions due to ALRIs, and 1% of ALRI-associated mortality could be attributed to human metapneumovirus. About 58% of these hospital admissions occurred in the first year of life. About 64% of the in-hospital deaths occurred in infants younger than 6 months, including 79% occurring in low-income and lower-middle-income countries.

**Implications of all the available evidence**

To our knowledge, our systematic review provides the first estimates of global human metapneumovirus-associated ALRIs and human metapneumovirus-attributable ALRI morbidity and mortality in children under 5 years overall and stratified by narrow age bands (0–5 months, 6–11 months, and 12–59 months). Our results indicate that infants younger than 1 year have an increased risk of severe human metapneumovirus-associated ALRIs compared with older children. Infants younger than 6 months, especially those in low-income and lower-middle-income countries, are at greater risk of death from human metapneumovirus-associated ALRIs than older infants and those from upper middle-income countries and high-income countries. These findings warrant continued efforts to develop targeted intervention strategies to protect infants from human metapneumovirus infections, and to improve the outcome of infants with human metapneumovirus-associated ALRIs, especially in low-income and lower-middle income countries. Such strategies could include specific antiviral therapeutics, monoclonal antibodies, or vaccination for children and their mothers during pregnancy.

A live-attenuated recombinant human metapneumovirus vaccine has been assessed in a phase 1 clinical trial; however, study findings showed that the vaccine had insufficient immunogenicity in children aged 6–59 months.^13^ Antivirals against human metapneumovirus infections and several other types of vaccines have been investigated, but have not reached clinical trials.^14^, ^15^, ^16^

We aimed to estimate the global and regional number of human metapneumovirus-associated ALRI cases, hospital admissions, and mortality by age strata in children under 5 years for 2018, using published and unpublished data on laboratory-confirmed (culture, immunofluorescence assay, or molecular test) human metapneumovirus morbidity and mortality. Since the presence of human metapneumovirus in the upper respiratory tract in children does not indicate that ALRI symptoms were directly caused by these viruses, we also estimated the global burden of ALRI that is attributable to human metapneumovirus by accounting for the causal attribution of human metapneumovirus. The estimates would provide evidence for the further development of targeted interventions and treatment.

## Methods

### Systematic review

We did a systematic review of the human metapneumovirus-associated ALRI disease burden in children under 5 years (appendix pp 4–5). We searched MEDLINE (Ovid), Embase (Ovid), Global Health 1973 onwards (Ovid), CINAHL, Web of Science, Global Health Library, and three Chinese databases (CNKI, Wanfang, and Chongqing VIP) for studies published between Jan 1, 2001, and Dec 31, 2019. To search the grey literature, we did broad searches through Google using the same date restrictions and the search terms “acute respiratory infections” AND “child” AND “parainfluenza virus”. No language or publication restrictions were applied. Three authors (XW, LCV, and YL) screened the titles and abstracts for eligibility and extracted data independently. Disagreements were resolved by discussion between the authors.

We included studies that reported any of the following data in children younger than 5 years: community incidence rates of ALRIs (ie, clinical pneumonia according to 2005 WHO Integrated Management of Childhood Illnesses^17^) with laboratory-confirmed human metapneumovirus; rates of hospital admissions for ALRIs (ie, physician-confirmed diagnosis of ALRI) or ALRIs with hypoxaemia confirmed by pulse oximetry both with laboratory-confirmed human metapneumovirus (per 1000 children per year); proportion of ALRIs with laboratory-confirmed human metapneumovirus cases among hospitalised ALRI cases; or in-hospital case-fatality ratios (hCFRs) of ALRIs with laboratory-confirmed human metapneumovirus. Case definitions are included in the appendix (pp 2–3).^18^

Studies had to use clear case definitions for specimen collection and testing, and studies that reported incidence and hospital admission rate data had to include data for at least one complete calendar year (or at least one full season if in a temperate region). We included hCFR data for any length of study period. We included data on the proportion of human metapneumovirus-positive cases among hospitalised ALRI cases if reported for at least one full calendar year.

We excluded studies: without a clear denominator population at risk (limited to those reporting incidence and hospital admission rate data); those in which human metapneumovirus was not the primary outcome; those reporting modelled burden estimates; those in which human metapneumovirus infections were diagnosed on the basis of serology alone; and those only including population subgroups with high-risk conditions.

We supplemented data from published studies with high quality unpublished data (from ongoing studies or re-analysis of previously published studies) using agreed standardised approaches and definitions within the collaborative Respiratory Virus Global Epidemiology Network.^19^

### Assessment of risk of biases

We used a modified Newcastle-Ottawa Scale to assess the risk of bias in seven domains, including study design, adjustment for health utilisation, patient groups excluded, definition, sampling strategy, diagnostic testing, and hypoxaemia ascertainment (appendix pp 25–26).^18^, ^20^

### Statistical analysis

Our burden estimation approaches, including main analyses and sensitivity analyses, are summarised in the appendix (p 14)). We estimated the number of human metapneumovirus-associated ALRI cases, hospital admissions, and in-hospital deaths using a similar strategy to our previous analysis.^18^ For the incidence rate and hospital admission rate, we scaled the population-at-risk for the level of testing per study where available before meta-analyses (appendix p 23). In a meta-analysis, we estimated incidence rates, hospital admission rates, and hCFRs of human metapneumovirus-associated ALRI using a generalised linear mixed model.^21^

After meta-analyses, we estimated number of cases and number of hospital admissions using Monte Carlo simulation, which enabled the combination of meta-estimates and population estimates (UN 2018 population estimates).^22^ The median value of 10 000 samples simulated from a log-normal distribution was used as the point burden estimate and the 2·5th and 97·5th percentiles as the 95% uncertainty ranges (URs). We used the same strategy as our previous studies, for consistency; alternative methods (such as resampling observed data) are likely to yield comparable estimates.^23^ In the main analysis, we reported estimates stratified by three non-overlapping age groups (0–5 months, 6–11 months, and 12–59 months) and by child mortality level of the setting from 2018 data (stratified into low or high; using the median under-5 mortality rate [16·6 per 1000 livebirths] as the cutoff point) for each outcome where available.^24^ Global results were calculated as the sum of age-specific and region-specific estimates. The numbers of cases were rounded to the nearest thousand, and deaths were rounded to the nearest hundred. In the community setting, we reported the incidence rate for the overall age group (0–59 months) because data were insufficient to allow disaggregation by narrower age bands. To incorporate information from studies with data for other age bands (eg, 0–11 months), we imputed the numbers of cases for 0–59 months using a multiple imputation approach as previously described (appendix pp 21–22).^18^

We estimated human metapneumovirus-associated ALRI in-hospital deaths by combining the estimates of hospital admissions and hCFRs of human metapneumovirus-associated ALRIs.^18^, ^19^ Similar to morbidity estimation, global estimates of mortality were calculated as the sum of the estimates, stratified by the three age groups and by child mortality settings.

Many child deaths from ALRIs occur outside of hospitals, especially in resource-limited settings due to low care-seeking or restricted access to care. Thus, we estimated an inflation factor ratio of overall ALRI deaths to in-hospital ALRI deaths at eight sites in six countries with high child mortality (Mozambique, Kenya, South Africa, Burkina Faso, and Ghana in sub-Saharan Africa, and Bangladesh in south Asia).^18^, ^25^, ^26^, ^27^ We calculated the median inflation factor, which was extrapolated to other countries with high child mortality, and we applied this inflation factor to estimates of in-hospital deaths to yield overall human metapneumovirus-associated ALRI mortality (appendix p15).^18^, ^19^, ^28^ We assumed that human metapneumovirus prevalence was the same in ALRI community deaths and in-hospital deaths. For settings with low child mortality, the reciprocal of the proportion of children with pneumonia symptoms who received care, measured in Multiple Indicator Cluster Surveys, Demographic and Health Surveys, and other national surveys, was used as a proxy for the inflation factor.^29^ The inflation factor can be calculated for many countries and regions, and it can thus help improve the estimates of overall human metapneumovirus-associated ALRI mortality. The median inflation factor for low child mortality settings was extrapolated to other countries with low child mortality without available data. Compared with approximating the inflation factor using non-specific ALRI mortality, in this approach we additionally assumed that the case-fatality ratios for pneumonia in hospitalised and non-hospitalised cases were broadly similar.

The presence of human metapneumovirus in the upper respiratory tract of a child with ALRI does not indicate that human metapneumovirus caused the ALRI symptoms in every instance. Thus, we estimated the ALRI burden that is causally attributable to human metapneumovirus (ie, human metapneumovirus-attributable ALRI burden) by combining the human metapneumovirus-associated ALRI burden estimates and the proportion of laboratory-confirmed human metapneumovirus ALRI cases or deaths that are attributable to human metapneumovirus (the attributable fraction of human metapneumovirus in human metapneumovirus-associated ALRI cases or deaths). We used a median value of 78% as the input for the attributable fraction of human metapneumovirus-associated ALRI cases based on three multicountry studies.^3^, ^4^, ^5^ The attributable fraction of human metapneumovirus-associated ALRI deaths was calculated using the attributable fraction of human metapneumovirus-associated ALRI cases and the ratio between hCFRs of human metapneumovirus-positive ALRIs and human metapneumovirus-negative ALRIs. We assumed that hCFR of human metapneumovirus-negative ALRIs was equal to the hCFR of human metapneumovirus-positive ALRIs that were not deemed attributable to human metapneumovirus (appendix p 17).

We did sensitivity analyses for human metapneumovirus-associated ALRI morbidity and in-hospital deaths (appendix pp 9–13), and estimated burden by country development status, according to UNICEF definitions and World Bank income levels (low-income and lower-middle-income, upper-middle-income, and high-income).^30^, ^31^ Additionally, we estimated the range of human metapneumovirus-associated ALRI hospital admissions by combining the proportion of hospitalised ALRI cases positive for human metapneumovirus and the 2015–16 estimates of all-cause ALRI hospital admissions among children younger than 5 years.^32^, ^33^ We also estimated overall human metapneumovirus-associated ALRI deaths for settings with high child mortality by applying the proportion of human metapneumovirus-positive deaths among all ALRI deaths to the number of ALRI deaths in children under 5 years for 2017.^1^ The proportion of human metapneumovirus-positive deaths among all ALRI deaths was estimated using data from hospital-based studies included in the systematic review.

As a final sensitivity analysis, we estimated human metapneumovirus-attributable mortality for settings with high child mortality by applying the proportion of human metapneumovirus–attributable ALRI deaths to the number of ALRI deaths in children under 5 years from 2017.^1^ The proportion of human metapneumovirus-attributable ALRI deaths was estimated using data for December, 2016, to October, 2019, obtained from Child Health and Mortality Prevention Surveillance (appendix p 18).^34^ Details of included studies are in the appendix (pp 34–53, 60–67). All analyses were done in R (version 3.5.2).^35^ This study was done in accordance with the Guidelines for Accurate and Transparent Health Estimates Reporting recommendations (appendix p 59).^36^

### Role of the funding source

The funder of the study had no role in study design, data collection, data analysis, data interpretation, or writing of the report. XW and HN had full access to all the data in the study and HN had final responsibility for the decision to submit for publication.

## Results

We identified 159 studies with data on human metapneumovirus-associated ALRI community incidence, hospital admission rates, the proportion of human metapneumovirus-positive cases among hospitalised ALRI cases, and hCFRs (figure); 119 studies were published and 40 were unpublished studies from the Respiratory Virus Global Epidemiology Network. Of the 159 studies included, seven (4%) were from low-income countries, 36 (23%) from lower-middle-income countries, 67 (42%) from upper-middle-income countries, and 49 (31%) from high-income countries. The characteristics of the included studies are summarised in the appendix (pp 6–7, 34–53).

**Figure:**
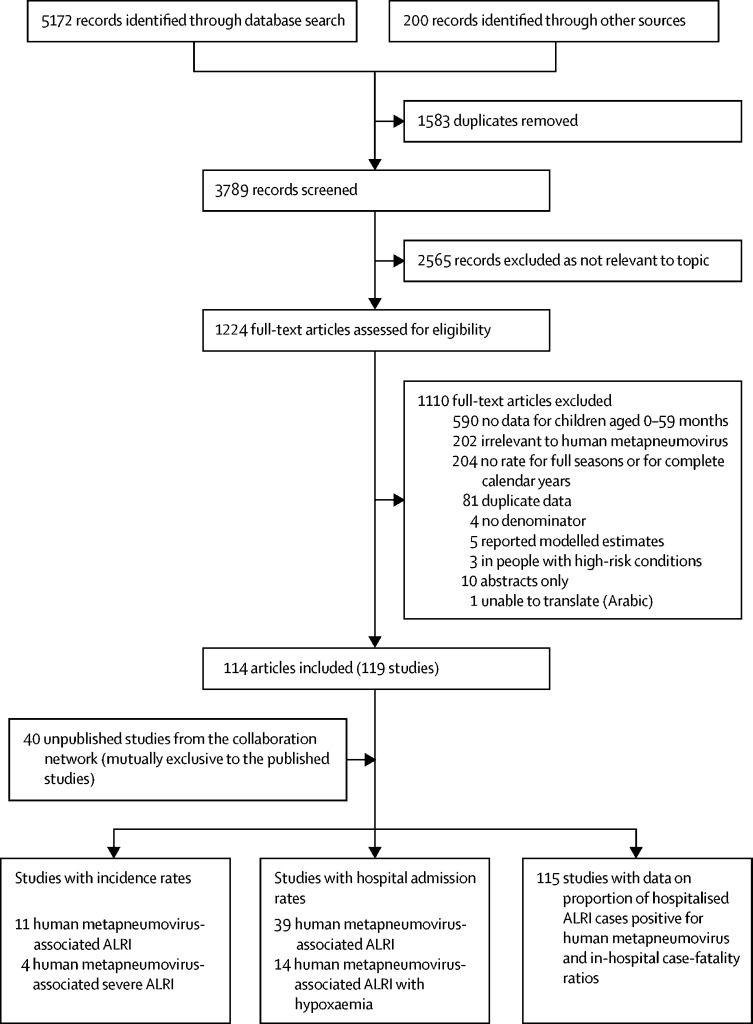
Flow diagram for selection of studies on human metapneumovirus-associated ALRIs ALRIs=acute lower respiratory infections.

11 studies reported the incidence of human metapneumovirus-associated ALRIs (ie, ALRI cases with laboratory confirmation of human metapneumovirus). Ten studies provided data for the 0–59-month age group after imputation, including six studies from settings with high child mortality; five studies were from lower-middle-income countries, two studies from upper-middle-income countries, and three studies from high-income countries (table 1). Four studies reported incidence before 2010. Between 1990 and 2015, the mean human metapneumovirus-associated ALRI incidence meta-estimate was 22·1 per 1000 children per year (95% CI 17·0–28·7) in children aged 0–59 months in settings with high child mortality, and 18·9 (11·2–31·9) in settings with low child mortality. The high incidence observed in settings with low child mortality was mainly driven by two Australian studies (done between 1996 and 1999 and between 2010 and 2014).^37^, ^38^ Using these meta-estimates, we estimated that there were 14·2 million human metapneumovirus-associated ALRI cases (UR 10·2 million to 20·1 million) globally in children younger than 5 years.

**Table 1:** Estimates of the incidence (per 1000 children per year) and number of human metapneumovirus-associated acute lower respiratory infection cases in children younger than 5 years in the community in 2018, by age group, and by World Bank income level and level of child mortality by setting (low *vs* high)

	**LMICs**	**UMICs**	**HICs**	**Settings with low child mortality**	**Settings with high child mortality**
**0–5 months**					
Studies, n	5	1	0	0	6
Incidence (per 1000 children per year)*	30·2 (7·8–109·8)	NA	NA	NA	35·4 (12·0–99·3)
Episodes (thousands)	1337 (359–4983)	NA	NA	NA	1629 (570–4662)
**6–11 months**					
Studies, n	5	1	0	0	6
Incidence (per 1000 children per year)*	30·7 (12·2–75·1)	NA	NA	NA	32·8 (16·0–65·9)
Episodes (thousands)	1347 (546–3327)	NA	NA	NA	1496 (740–3026)
**12–59 months**					
Studies, n	4	1	0	0	5
Incidence (per 1000 children per year)*	23·1 (16·7–31·9)	NA	NA	NA	21·0 (15·4–28·6)
Episodes (thousands)	7923 (5743–10 933)	NA	NA	NA	7490 (5506–10 192)
**0–59 months**†					
Studies, n‡	5 (1)	2 (1)	3 (2)	4 (3)	6 (1)
Incidence (per 1000 children per year)*	21·3 (15·5–29·3)	16·5 (8·3–32·8)	22·6 (13·4–38·2)	18·9 (11·2–31·9)	22·1 (17·0–28·7)
Episodes (thousands)	9202 (6711–12 620)	3019 (1521–5996)	1436 (854–2415)	4347 (2588–7304)§	9891 (7620–12 842)§

Data in parentheses are estimated uncertainty ranges. Data were imputed using a multiple imputation approach. Meta-analyses were only done when two or more studies were available. Settings in which the under-5 mortality rate was higher than the median under-5 mortality rate for 2018, were considered high child mortality settings, and settings in which the under-5 mortality rate was lower than the median under-5 mortality rate for 2018, were considered low child mortality settings. LMICs=lower-middle income countries. UMICs=upper middle-income countries. HICs= high-income countries. NA=not available.

* Derived from meta-analysis.

† Estimates were calculated by applying rates for children aged 0–59 months to the population estimates.

‡ Data in parentheses are the number of imputed studies.

§ Global number of human metapneumovirus-associated acute lower respiratory infections cases in children aged 0–59 months was 14·2 million (uncertainty range 10·2 million to 20·1 million), which was calculated by adding estimates from the two child mortality setting groups.

We identified 39 studies reporting human metapneumovirus-associated ALRI hospital admission rates, including 29 studies reporting data by three narrow age groups (0–5 months, 6–11 months, or 12–59 months; table 2). The hospital admission rate meta-estimate was more than four times higher in infants aged 0–5 months and 6–11 months (2·2–3·3 admissions per 1000 children per year) than children aged 12–59 months (0·3–0·6 admissions per 1000 children per year) when stratified by World Bank income levels and child mortality settings. In the analysis stratified by child mortality settings, we estimated that there were 643 000 human metapneumovirus-associated ALRI hospital admissions (UR 425 000–977 000) globally in children younger than 5 years in 2018, of which 374 000 (58%) occurred in infants.

**Table 2:** Hospital admission rates (per 1000 children per year) and hospital admissions of human metapneumovirus-associated ALRI in children younger than 5 years in 2018, by age group and by World Bank income levels and level of child mortality by setting (low *vs* high)

		**LMICs**	**UMICs**	**HICs**	**Settings with low child mortality**	**Settings with high child mortality**	Global estimates*
**Human metapneumovirus-associated ALRIs**							
0–5 months							
	Studies, n	8	6	5	7	12	..
	Hospital admission rate (per 1000 children per year)†	2·4 (1·6–3·5)	3·3 (1·6–7·1)	3·3 (2·2–5·1)	2·7 (1·8–4)	3·0 (1·9–4·9)	..
	Hospital admissions (thousands)	106 (72–157)	61 (29–128)	21 (14–32)	62 (42–93)	138 (86–221)	200 (128–314)
6–11 months							
	Studies, n	7	5	4	5	11	..
	Hospital admission rate (per 1000 children per year)†	2·7 (1·7–4·3)	2·5 (1–5·9)	2·8 (2·2–3·5)	2·2 (1·5–3·4)	2·7 (1·7–4·4)	..
	Hospital admissions (thousands)	119 (75–188)	46 (19–111)	18 (14–22)	51 (34–76)	123 (77–198)	174 (110–274)
12–59 months							
	Studies, n	9	8	5	7	15	..
	Hospital admission rate (per 1000 children per year)†	0·6 (0·3–1)	0·4 (0·2–0·8)	0·3 (0·2–0·7)	0·3 (0·2–0·5)	0·6 (0·4–0·8)	..
	Hospital admissions (thousands)	206 (113–375)	59 (29–117)	15 (8–28)	55 (35–87)	214 (152–302)	269 (187–389)
0–59 months‡							
	Hospital admissions (thousands)	431 (260–720)	165 (77–356)	54 (36–83)	168 (110–255)	475 (315–721)	643 (425–977)
**Human metapneumovirus-associated ALRIs with hypoxaemia**							
0–5 months							
	Studies, n	6	3	1	2	8	..
	Hospital admission rate (per 1000 children per year)†	0·3 (0–2·1)	1·3 (0·3–5·4)	NA	0·6 (0·2–1·5)	0·7 (0·2–2·5)	..
	Hospital admissions (thousands)	13 (1–268)	24 (6–101)	NA	14 (5–38)	32 (9–113)	46 (14–151)
6–11 months							
	Studies, n	6	3	1	2	8	..
	Hospital admission rate (per 1000 children per year)†	0·4 (0·1–2)	0·3 (0·1–1·2)	NA	0·3 (0·1–1·7)	0·5 (0·2–1·7)	..
	Hospital admissions (thousands)	18 (4–78)	6 (2–19)	NA	7 (2–28)	23 (8–66)	30 (10–94)
12–59 months							
	Studies, n	6	4	1	3	8	..
	Hospital admission rate (per 1000 children per year)†	0·1 (0–0·2)	0·1 (0–0·7)	NA	0 (0–0·1)	0·1 (0–0·3)	..
	Hospital admissions (thousands)	34 (5–215)	15 (1–171)	NA	1 (0–4)	36 (5–273)	37 (5–277)
0–59 months‡							
	Hospital admissions (thousands)	65 (10–560)	44 (9–291)	NA	22 (7–70)	91 (22–452)	112 (29–522)

Data in parentheses are estimated uncertainty ranges. Meta-analyses were only done when at least two studies were available. Settings in which the under-5 mortality rate was higher than the median under-5 mortality rate for 2018, were considered high child mortality settings, and settings in which the under-5 mortality rate was lower than the median under-5 mortality rate for 2018, were considered low child mortality settings. ALRI=acute lower respiratory tract infection. LMICs=lower-middle-income countries. UMICs=upper-middle-income countries. HICs=high-income countries. NA=not available.

* Global estimates by age strata are the sum of estimates by child mortality settings; global estimates for 0–59 months are the sum of estimates by age groups and child mortality settings.

† Derived from the meta-analysis.

‡ Estimates are the sum of estimates for all three age groups (0–5 months, 6–11 months, and 12–59 months).

14 studies included hospital admission rates for human metapneumovirus-associated ALRIs with hypoxaemia by three age groups, including five studies from settings with low child mortality (table 2). In the analysis stratified by child mortality settings, we estimated that, globally, 112 000 (UR 29 000–522 000) hospital admissions were for human metapneumovirus-associated ALRIs with hypoxaemia in children aged 0–59 months in 2018, accounting for 17% (ie, 112 000 of 643 000 cases) of the human metapneumovirus-associated ALRI hospital admissions.

73 studies reported hCFRs for human metapneumovirus-associated ALRI in children younger than 5 years, including 28 studies with data stratified by three narrow age bands (table 3). hCFRs were highest in infants aged 0–5 months from settings with high child mortality (3·3%, 95% CI 1·7–6·1) and from lower-middle-income countries (4·5%, 2·3–8·6). hCFRs were lower in children from high-income countries who were aged 6–11 months (0·6%, 0·1–3·9) and 12–59 months (0·5%, 0–7·0) than infants aged 0–5 months from high child mortality settings and from lower-middle-income countries, with the estimates in older children having notably wide confidence intervals. On the basis of these meta-estimates, we estimated that there were 7700 human metapneumovirus-associated ALRI in-hospital deaths (UR 2600–48 800) in children younger than 5 years in 2018, of which 5500 (71%) occurred in infants. About 64% of these deaths were in young infants aged 0–5 months (4900 deaths of 7700 total in-hospital deaths), and 88% (6800 deaths of 7700 total in-hospital deaths) occurred in countries with high child mortality.

**Table 3:** hCFR meta-estimates of human metapneumovirus-associated ALRI and in-hospital deaths in children younger than 5 years in 2018, by age group and by World Bank income levels and level of child mortality by setting (low *vs* high)

		**LMICs**	**UMICs**	**HICs**	**Settings with low child mortality**	**Settings with high child mortality**	Global estimates*
Studies, n		15	6	7	7	21	..
0–5 months							
	hCFR, %†	4·5% (2·3–8·6)	1·7% (0·6–5·1)	0·4% (0–8·6)	0·4% (0–8·6)	3·3% (1·7–6·1)	..
	Deaths, n	4300 (2000–9200)	1000 (300–3700)	100 (0–3400)	200 (0–9900)	4600 (2100–9900)	4900 (2100–19 300)
6–11 months							
	hCFR, %†	0·7% (0–9)	NA	0·6% (0·1–3·9)	0·6% (0·1–3·9)	0·2% (0–7·6)	..
	Deaths, n	900 (0–37 800)	NA‡	100 (0–700)	300 (0–1900)	200 (0–9400)	600 (100–11 300)
12–59 months							
	hCFR, %†	0·9% (0·3–2·8)	1·1% (0·1–9·1)	0·5% (0–7·0)	0·5% (0–7)	0·9% (0·2–3·6)	..
	Deaths, n	1800 (500–6500)	600 (100–6600)	100 (0–2800)	300 (0–10 100)	1900 (400–8300)	2200 (400–18 300)
0–59 months§							
	Deaths, n	7200 (2600–52 300)	1700 (300–10 300)	300 (0–6900)	800 (100–22 200)	6800 (2500–27 100)	7700 (2600–48 800)

Data in parentheses are estimated uncertainty ranges. Settings in which the under-5 mortality rate was higher than the median under-5 mortality rate for 2018, were considered high child mortality settings, and settings in which the under-5 mortality rate was lower than the median under-5 mortality rate for 2018, were considered low child mortality settings. hCFR=in-hospital case-fatality ratio. ALRI=acute lower respiratory tract infection. LMICs=lower-middle-income countries. UMICs=upper middle-income countries. HICs=high-income countries. NA=not available.

* Global estimates by age strata are the sum of estimates by child mortality settings; global estimates for children aged 0–59 months are the sum of estimates by age and child mortality settings.

† Estimates derived from the meta–analysis.

‡ No human metapneumovirus-associated ALRI deaths were reported in any of the studies from upper middle-income countries; thus hCFR for the strata was not estimated.

§ Estimates for the 0–59 month age group are the sum of estimates for the three age groups (0–5 months, 6–11 months, and 12–59 months).

Across 28 countries or regions with low child mortality, 22–94% of children with pneumonia received care from a health provider (appendix p 16). About 71% of the data were for the period 2010–14. On the basis of these data, we estimated the median inflation factor was 1·3 across regions or countries, and thus that there were about 1100 human metapneumovirus-associated ALRI deaths (UR 100–28 800; after rounding in-hospital deaths), in hospitals and in the community, among children younger than 5 years in settings with low child mortality in 2018. Of the eight sites with data on pneumonia mortality in high child mortality settings, five sites were in rural areas, and six sites were in African countries. Six studies reported data between 2010 and 2016, and the remaining two studies reported data from 2008 onwards. The inflation factor ranged from 1·5 to 3·5 across the eight sites, with a median value of 2·2 (IQR 1·7–2·7). Thus, we estimated that there were 14 900 human metapneumovirus-associated ALRI deaths (UR 5600–59 700; after rounding in-hospital deaths), in hospitals and in the community, in settings with high child mortality. We therefore estimate that there were 16 100 human metapneumovirus-associated ALRI deaths (5700–88 000; ie, ALRI deaths in children with laboratory-confirmed human metapneumovirus) among children globally in 2018.

By applying the attributable fraction of human metapneumovirus to the estimated human metapneumovirus-associated burden, we estimated that 11·1 million ALRI cases (UR 8·0 million to 15·7 million) and 502 000 ALRI hospital admissions (UR 332 000–762 000) could be attributed to human metapneumovirus in children under 5 years (table 4). We estimated that the ratio of in-hospital case fatalities of human metapneumovirus-attributable ALRIs to human metapneumovirus-associated cases was 0·9 (appendix p 18), and the attributable fraction for human metapneumovirus-associated ALRI deaths was 70% (appendix pp 17–18), indicating that 11 300 ALRI deaths (UR 4000–61 600) globally could be attributed to human metapneumovirus in 2018, including 10 400 deaths (3900–41 800) in settings with high child mortality (table 4).

**Table 4:** Estimates of global human metapneumovirus-attributable ALRI cases, hospital admissions, and mortality, projected using attributable fractions that were calculated by use of inflation factors

	Attributable fraction, %*	**Global human metapneumovirus-associated burden estimates**	**Global human metapneumovirus-attributable burden estimates**†
Human metapneumovirus-attributable ALRI cases (millions)	78%‡	14·2 (10·2–20·1)	11·1 (8·0–15·7)
Human metapneumovirus-attributable ALRI hospital admissions (thousands)	78%	643 (425–977)	502 (332–762)
Human metapneumovirus-attributable ALRI deaths	70%§	16 100 (5700–88 000)	11 300 (UR 4000–61 600)

Data in parentheses are uncertainty ranges. ALRI=acute lower respiratory tract infection.

* The proportion of human metapneumovirus-positive cases and deaths attributable to human metapneumovirus.

† Applying the corresponding attributable fraction to the estimates of human metapneumovirus-associated burden.

‡ The attributable fraction for human metapneumovirus-associated ALRI cases was calculated using odds ratios from a systematic review and two multi-country studies; the median odds ratio from the three studies was input to yield the attributable fraction for human metapneumovirus-ALRI cases (78%); all references are in the appendix (p 18).

§ The attributable fraction for human metapneumovirus-associated ALRI deaths was modelled using the attributable fraction for human metapneumovirus cases and the ratio of case-fatality between human metapneumovirus-attributable cases and human metapneumovirus-associated cases (appendix pp 17–18).

We did several sensitivity analyses to estimate hospital admissions, in-hospital deaths, and overall human metapneumovirus-associated ALRI deaths, and of human metapneumovirus-attributable ALRI deaths (by country development strata and World Bank income level) using different approaches (appendix pp 8–13,16,19). For global human metapneumovirus-associated ALRI hospital admissions, the estimates in children younger than 5 years ranged from 626 000 to 650 000 in analyses by different stratification groups (appendix p 9); the proportion-based approach yielded a broader range (282 000–902 000; appendix p 10). The point estimate of global in-hospital deaths ranged from 7200 to 9100 in children younger than 5 years, and an estimated 79% of the global in-hospital deaths occurred in low-income and lower-middle-income countries (7200 of 9100 deaths) when stratified by World Bank income level (appendix p 11). We estimated 19 900 human metapneumovirus-associated ALRI deaths (UR 12 100–33 200) and 9900 human metapneumovirus-attributable ALRI deaths (UR 2600–39 300) in children aged 1–59 months in settings with high child mortality using a different approach (appendix pp 16, 19).

## Discussion

To our knowledge, this is the first study to report global human metapneumovirus-associated ALRI burden estimates both among children younger than 5 years overall and stratified by narrow age groups (0–5 months, 6–11 months, and 12–59 months). When compared with our previously published all-cause ALRI burden estimates, human metapneumovirus is identified in 11% of ALRI cases, 4–13% of ALRI hospital admissions, and 2% of ALRI deaths among children under 5 years globally.^1^, ^32^, ^33^ About 8% of ALRI cases, 3–10% of ALRI hospital admissions, and 1% of ALRI mortality can be attributed to human metapneumovirus. The wide URs of these burden estimates reflect differences across studies, arising from variation in human metapneumovirus epidemiology between populations and methodological differences, and from paucity of data, especially mortality data.

Similar to respiratory syncytial virus and influenza, we found that the human metapneumovirus-associated ALRI hospital admission rate was much higher in infants than older children, with about 58% of hospital admissions and 71% of in-hospital deaths due to human metapneumovirus-associated ALRIs in children under 5 years occurring in the first year of life.^18^, ^19^ The high burden in infants could be due to the immaturity of infants' immune systems and decaying maternal antibodies during the first several months of life.^39^, ^40^ The consistently high human metapneumovirus-associated ALRI hospital admission rate among infants across different settings highlights the importance of developing safe and effective maternal human metapneumovirus vaccines, particularly for infants.

Our hCFR estimates show that young infants aged 0–5 months are at an increased risk of human metapneumovirus–associated ALRI mortality compared with infants older than 5 months. hCFR estimates for infants aged 0–5 months varied substantially across settings, possibly reflecting the differences in disease severity at admission and in the quality of hospital care. Increased disease severity at presentation could be associated with the high prevalence of certain underlying conditions and delays in care-seeking.^3^, ^33^ The case-fatality ratio (overall and in-hospital) and overall mortality due to ALRI among children under 5 years has substantially reduced in the past 15 years due to socioeconomic development, reduced prevalence of pneumonia risk factors, and increased use of interventions.^33^, ^41^ However, most studies spanning several years reported one to three deaths from human metapneumovirus-associated ALRIs, and we were unable to identify any changes in the hCFR of human metapneumovirus-associated ALRI over time. When stratifying the data using 2010 as a cutoff and using available age-stratified data from studies done before 2010 (eligible if part of a study was done before 2010 and data could not be stratified by year), we estimated that there were 9600 human metapneumovirus-associated ALRI in-hospital deaths (UR 2300–51 200) among children under 5 years in 2010 (appendix p 12).

For settings with high child mortality, the inflation factor was based on scarce data.^19^ The estimates could be biased due to limited generalisability and location-specific characteristics (ie, prevalence of human metapneumovirus in childhood ALRI deaths). However, our reported estimates of overall human metapneumovirus-associated ALRI deaths for settings with high child mortality are conservative and could increase by approximately 30% (19 900 deaths) using an alternative estimation approach (appendix p 16). For settings with low child mortality, the inflation factor and overall mortality estimate are likely to be underestimated because the definition of care-seeking is broader than the definition of in-hospital care: contact with primary care is included as care-seeking in surveys, but not included in the in-hospital mortality estimates in the present analysis. The US vital statistics data showed that about 40% of under-5 ALRI deaths (International Classification of Diseases, 10th revision diagnostic codes J09–22; U04) occurred in outpatient or emergency departments between 2010 and 2017.^42^ Additionally, this analysis was based on one further assumption that no differences exist between the case-fatality ratio for hospitalised and non-hospitalised pneumonia cases. The direction of bias associated with this assumption could be complicated by the two-way association between disease severity and care-seeking: children with more severe symptoms are more likely to receive hospital care; whereas, supportive care in hospitals can reduce the risk of death, and lack of appropriate care or delays in care can lead to rapid deterioration.^43^, ^44^, ^45^ Moreover, data on pneumonia care-seeking in high-income countries were not available in published reports; extrapolation from other countries with low child mortality to high-income countries might result in bias. The estimates of inflation factor could also be affected by accuracy of assessment and completeness of documentation for ALRI or pneumonia. For example, the diagnosis of (presumptive) pneumonia in the UNICEF dataset is based on caregivers' report, thus might be inaccurate and affect the estimates of inflation factor.

Our study had several other limitations. Little data were available for Europe and Latin America, which could have affected the generalisation of the estimates. Moreover, heterogeneity in the methodology (ie, variations in precise case definitions, proportion of eligible ALRI cases tested for human metapneumovirus) existed across studies for each outcome and is likely to have biased our estimates (appendix p 7). We adjusted for levels of testing when estimating hospital admission rates by assuming that the proportion of tested patients with ALRIs who were positive for human metapneumovirus was the same as the proportion in untested patients with ALRIs. However, we did not adjust for the underdetection of human metapneumovirus when estimating hCFRs. The hCFR of human metapneumovirus-associated ALRIs and in-hospital mortality might be underestimated, as suggested by the higher hCFR in patients tested than untested patients (appendix p 24). The incidence and hospital admission rates of human metapneumovirus-associated ALRI are unlikely to be affected by the accuracy of test methods since 92% of the studies used PCR to detect human metapneumovirus. In addition to potential biases, we summed yearly data to ensure precision in the age-stratified analysis; however, we did not account for variation across years (appendix p 20).

Our results show that infants younger than 1 year have disproportionately high risks of severe human metapneumovirus infections across settings. Infants aged 0–5 months in low-income and lower-middle-income countries have an increased risk of mortality from human metapneumovirus-associated ALRIs compared with those in upper-middle-income countries and high-income countries, accounting for nearly eight of every ten global deaths occurring in this age group. These estimates highlight the importance of interventions for infants. In low-income and lower-middle-income countries, continued efforts are needed to reduce human metapneumovirus-associated ALRI mortality among young children, especially infants, in hospitals and communities through improving access to care and case management in hospitals. Considering the paucity of data at the regional level, these estimates should be viewed as preliminary estimates. In the future, additional high quality data on childhood human metapneumovirus-associated ALRI cases, hospital admissions, and mortality, especially age-stratified data, would help refine the estimates and track changes in human metapneumovirus-associated ALRI morbidity and mortality over time.
