# Supplementary material for: Global burden of acute lower respiratory infection associated with human metapneumovirus in children under 5 years in 2018: a systematic review and modelling study
Source: Lancet Glob Health. 2020 Nov 26;9(1):e33–43. doi: 10.1016/S2214-109X(20)30393-4 (PMC7783516; doi:10.1016/S2214-109X(20)30393-4)
Supplement: Supplementary appendix [file mmc1.pdf]

THE LANCET
Global Health

Supplementary appendix

This appendix formed part of the original submission and has been peer reviewed. We post it as supplied by the authors.

Supplement to: Wang X, Li Y, Deloria-Knoll M, et al. Global burden of acute lower respiratory infection associated with human metapneumovirus in children under 5 years in 2018: a systematic review and modelling study. *Lancet Glob Health* 2020; published online Nov 26. http://dx.doi.org/10.1016/S2214-109X(20)30393-4.

Supplementary materials for "Global burden of acute lower respiratory infections associated with human metapneumovirus in children under five years for 2018: a systematic review and modelling analysis"

Respiratory Virus Global Epidemiology Network

Harish Nair, Harry Campbell, Xin Wang, You Li, Linda C Vaccari, Ting Shi (University of Edinburgh, Scotland, UK); Anand Krishnan, Ritvik Amarchand, Avinash Choudekar (All India Institute of Medical Sciences, New Delhi, India); F Xavier López-Labrador, Ainara Mira-Iglesias, Joan Puig-Barberà (Área de Investigación en Vacunas, Fundación para el Fomento de la Investigación Sanitaria y Biomédica de la Comunitat Valenciana, Valencia, Spain); Cheryl Cohen, Florette K Treurnicht, Orienka Hellferscee, Sibongile Walaza (Centre for Respiratory Disease and Meningitis, National Institute for Communicable Diseases, Johannesburg, South Africa); Marcela Echavarria (Clinical Virology Unit, Centro de Educación Médica e Investigaciones Clínicas, Argentina); Sudha Basnet (Deparment of Child Health, Tribhuvan University, Katmandu, Nepal and the Centre for International Health, University of Bergen, Norway); Katherine L. O'Brien, Maria Deloria-Knoll, Melissa M. Higdon, Meredith Haddix, W. Abdullah Brooks (Department of International Health, Johns Hopkins Bloomberg School of Public Health, Baltimore, Maryland, USA); Lay-Myint Yoshida, Michiko Toizumi (Department of Pediatric Infectious Diseases, Institute of Tropical Medicine, Nagasaki University, Japan); Asad Ali (Department of Pediatrics and Child Health, Aga Khan University, Pakistan); Eric AF Simões (Department of Pediatrics, Section of Infectious Diseases, University of Colorado, School of Medicine; Department of Epidemiology and Center for Global Health, Colorado School of Public Health, Aurora CO, USA); Vahid Salimi (Department of Virology, School of Public Health, Tehran University of Medical Sciences, Tehran, Iran); Heather J. Zar (Department of Paediatrics & Child Health, and MRC unit on Child & Adolescent Health, University of Cape Town, South Africa); Julia M Baker, Zeba A Rasmussen, Elizabeth D Thomas, Alexandra F Jamison (Fogarty International Center, NIH, USA); Romina Libster, Mauricio T Caballero (Fundacion INFANT, Argentina); Marta Werner (Hospital Guillermo Grant Benavente, Concepcion, Chile); Tor A. Strand (Innland Hosptial Trust, Lillehammer, Norway and the Centre for International Health, University of Bergen, Norway); Nusrat Homaira (International Centre for Diarrhoeal Disease Research, Bangladesh, icddr,b, Dhaka, Bangladesh and School of Women's and Children's Health, UNSW Sydney, Australia); Doli Goswami, Mustafizur Rahman (International Centre for Diarrhoeal Disease Research, Bangladesh, icddr,b); Miguel Lanaspa, Imane Jroundi, Lola Madrid, Quique Bassat, Cinta Moraleda (ISGlobal, Barcelona Ctr Int Health Res, Hospital Clínic—Universitat de Barcelona, Barcelona, Spain); Siddhivinayak Hirve, Sanjay Juvekar (KEM Hospital Research Center, India); Mandeep Chadha (National Institute of Virology, Pune, India; Grieven P. Otieno, Betty E Owor, D. James Nokes (KEMRI-Wellcome Trust Research Programme, Kilifi, Kenya); Shabir A. Madhi, Marta Nunes, Vicky Baillie, Matt Laubscher (Medical Research Council: Respiratory and Meningeal Pathogens Research Unit; Department of Science and Technology/National Research Foundation: Vaccine Preventable Diseases, University of the Witwatersrand, Faculty of Health Sciences, Johannesburg, South Africa); Hanna Nohynek (National Institute for Health and Welfare, Helsinki, Finland); Dang Duc Anh , Hien-Anh Thi Nguyen (National Institute of Hygiene and Epidemiology, Hanoi, Vietnam); Rodrigo A. Fasce (Public Health Institute of Chile, Chile); Marilla G. Lucero, Socorro Lupisan (Research Institute for Tropical Medicine, Phillipines); Brunhilde Schweiger (Robert Koch Institute, Berlin, Germany); Fernando P. Polack (Fundacion INFANT, Argentina); Shobha Broor (SGT Univeristy Gurgaon, Haryana, India); Histoshi Oshitani (Tohoku University Graduate School of Medicine, Japan); Mark P Nicol, Lesley Workman (University of Cape Town and National

Health Laboratory Services, Cape Town, South Africa); Najwa Khuri-Bulos (University of Jordan school of medicine, Jordan); Maria Mathisen (Vestre Viken Hospital Trust, Drammen, Norway); Barbara A Rath, Patrick Obermeier (Vienna Vaccine Safety Initiative, Berlin, Germany); Malinee Chittaganpitch (Medical Sciences Technical Office, Department of Medical Sciences, MoPH, Thailand); Pongpun Sawatwong (Division of Global Health Protection, Thailand Ministry of Public Health - US Centers for Disease Control and Prevention Collaboration, Nonthaburi, Thailand); Henry C. Baggett (Global Disease Detection Center, Thailand Ministry of Public Health - US Centers for Disease Control and Prevention Collaboration, Nonthaburi, Thailand; Division of Global Health Protection, US CDC, Atlanta, USA); Donald M. Thea (Department of Global Health and Development, Boston University School of Public Health, Boston, MA, USA); Phil Seidenberg (Department of Global Health and Development, Boston University School of Public Health, Boston, MA, USA; Department of Emergency Medicine, University of New Mexico, Albuquerque, New Mexico, USA); Lawrence Mwanayanda (Department of Global Health and Development, Boston University School of Public Health, Boston, MA, USA; Right To Care-Zambia, Lusaka, Zambia); Stephen R.C.Howie (Medical Research Council Unit, The Gambia at London School of Hygiene & Tropical Medicine; Department of Paediatrics, University of Auckland, New Zealand); Martin Antonio (Medical Research Council Unit The Gambia at London School of Hygiene & Tropical Medicine); Bernard E. Ebruke (Medical Research Council Unit The Gambia at London School of Hygiene & Tropical Medicine); David P. Moore (Medical Research Council: Respiratory and Meningeal Pathogens Research Unit University of the Witwatersrand, Johannesburg, South Africa; Department of Paediatrics & Child Health, Chris Hani Baragwanath Academic Hospital and University of the Witwatersrand, South Africa); Vicky L. Baillie (Medical Research Council: Respiratory and Meningeal Pathogens Research Unit University of the Witwatersrand, Johannesburg, South Africa); Tanja Adams (National Health Laboratory Services, South Africa); Doli Goswami, Mustafizur Rahman, Mohammed Ziaur Rahman (International Centre for Diarrhoeal Disease Research, Bangladesh, icddr,b); J. Anthony G. Scott (KEMRI-Wellcome Trust Research Programme, Centre for Geographic Medicine Research - Coast, Kilifi, Kenya; Nuffield Department of Tropical Medicine, Oxford University, Oxford, UK; Department of Infectious Disease Epidemiology, London School of Hygiene & Tropical Medicine, London, UK); Susan Morpeth (KEMRI-Wellcome Trust Research Programme, Centre for Geographic Medicine Research - Coast, Kilifi, Kenya); Karen L. Kotloff (Department of Pediatrics and Department of Medicine, Center for Vaccine Development and Global Health, University of Maryland School of Medicine, Baltimore, MD, USA); Milagritos D. Tapia (Department of Pediatrics, Center for Vaccine Development and Global Health, University of Maryland School of Medicine, Baltimore, MD, USA); Samboa O. Sow (Centre pour le Développement des Vaccins – Mali, Bamako, Mali).

Contents

Respiratory Virus Global Epidemiology Network 2

Appendix 1. Case definitions and glossary 2

Appendix 2. Search strategy for hMPV 4

Appendix 3. Characteristics of included studies 6

Appendix 4. Details of sensitivity analysis and summary of overall approaches used in this study 8

Appendix 5. Overall hMPV-associated ALRI mortality 15

Appendix 6. Estimating the attributable fraction for hMPV-associated ALRI deaths 17

Appendix 7. Estimating hMPV-attributable ALRI mortality in high child mortality settings using CHAMPS data 19

Appendix 8. Yearly variation in the hMPV-associated ALRI hospital admission rate 20

Appendix 9. Data imputation 21

Appendix 10. Details of denominator scaling 23

Appendix 11. Assessment tool for risk of bias in individual studies 25

Appendix 12. Risk of bias in individual studies 27

Appendix 13. Details of individual studies 34

Appendix 14. Meta-estimates by narrow age groups 54

Appendix 15. Checklist of information that should be included in new reports of global health estimates 59

References 60

Appendix 1. Case definitions and glossary

Community-based studies: studies where eligible cases were actively identified through regular visits to households. We considered studies conducted in outpatient departments or general practitioners' rooms in industrialised countries as good proxies of community-based studies.
Hospital-based studies: studies where children are enrolled when admitted into hospital.

Case definition for community-based studies
hMPV-associated ALRI: cough or difficulty breathing with increased respiratory rate for age (cut-offs same as in WHO IMCI case definition) AND laboratory confirmed hMPV.
hMPV-associated severe ALRI: children aged 2-59 months - cough or difficulty in breathing with chest wall indrawing AND laboratory confirmed hMPV; children aged <2 months - increased RR (≥60 breaths/min) OR chest wall indrawing AND laboratory confirmed hMPV.
Case definition for hospital-based studies
Hospitalised hMPV-associated ALRI: all children with physician confirmed diagnosis of ALRI (pneumonia or bronchiolitis) that were hospitalised, or recommended hospital admission AND laboratory confirmed hMPV.
Hospitalised hMPV-associated ALRI with hypoxaemia: hospitalised ALRI cases with hypoxaemia (as defined below) AND laboratory confirmed hMPV.
Hypoxaemia: at altitude ≤2500 m above sea level, SpO_2 <90% in children aged 1-59 months and <88% for neonates; at altitude >2500 m above sea level, SpO_2 <87% in children aged 1-59 months and <85% for neonates.
The relationship between the case definitions of different severities is displayed in Figure S1.1 A and B.
We defined hMPV–associated ALRI burden as the burden of ALRI with laboratory confirmed hMPV.
We defined hMPV–attributable ALRI burden as the ALRI burden that are causally attributable to hMPV.
Some other abbreviations used in this supplemental material:

LIC: low income countries; LMIC: lower-middle income countries; UMIC: upper-middle income countries; HIC: high income countries as per World Bank Classification.
AFR: WHO African region; AMR: WHO Region of the Americas; EMR: WHO Eastern Mediterranean region; EUR: WHO European region; SEAR: WHO South-East Asian region; WPR: WHO Western Pacific region.
Neonates: children aged 0-27 days.
hMPV: human metapneumovirus.
ALRI: acute lower respiratory infection.
hCFR: in-hospital case-fatality ratio.
AF: attributable fraction.

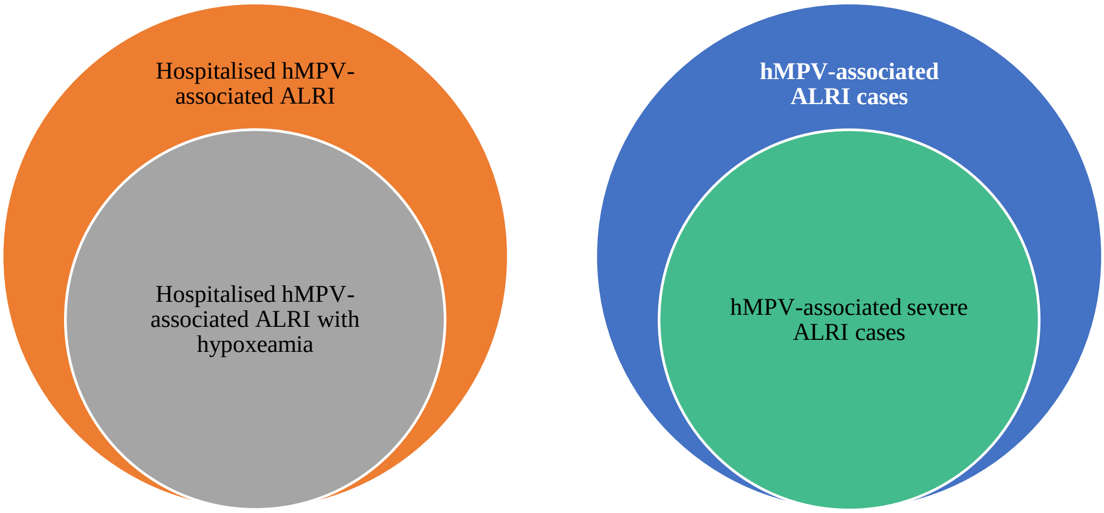

A B

Figure S1.1 The relationship between hospitalised hMPV-associated ALRI and hMPV-associated ALRI with hypoxaemia (A). The relationship between community hMPV-associated ALRI and hMPV-associated severe ALRI (B). The size of each circle is not proportionate to the number of cases for each severity.

Appendix 2. Search strategy for hMPV

Medline (Ovid)
1. exp Parainfluenza Virus 1, Human/ or exp Parainfluenza Virus 2, Human/ or exp Parainfluenza Virus 3, Human/ or exp Parainfluenza Virus 4, Human/ or exp Parainfluenza virus infection/ or infection, parainfluenza virus.mp. or infections, parainfluenza virus.mp. or virus infection, parainfluenza.mp. or virus infections, parainfluenza.mp. or parainfluenza vaccine.mp. or exp Parainfluenza Vaccines/ or PIV.mp. or Parainfluenza.mp.
2. metapneumovirus.mp. or exp metapneumovirus/ or hMPV.mp.
3. Bronchiolitis.mp. or exp Bronchiolitis/ or Bronchiolitis, Viral/
4. exp Respiratory Tract Diseases/
5. exp Respiratory Tract Infections/
6. acute respiratory infections.mp.
7. exp Pneumonia, Viral/ or *Pneumonia/ or exp Pneumonia/ or Pneumonia.mp.
8. acute lower respiratory infections.mp.
9. exp Incidence/ or exp Prevalence/ or exp morbidity/ or exp child mortality/ or exp infant mortality/ or exp hospital mortality/ or *hospital mortality/ or hospitalization rate.mp. or hospitalisation rate.mp. or exp Death/ or exp Cause of Death/ or burden.mp. /or proportion.mp.
10. 1 or 2
11. 3 or 4 or 5 or 6 or 7 or 8
12. 9 and 10 and 11
13. limit 12 to (humans and ("all infant (birth to 23 months)" or "newborn infant (birth to 1 month)" or "infant (1 to 23 months)" or "preschool child (2 to 5 years)"))
14. limit 14 to yr="1995 -2019"

Embase (Ovid)
1. exp parainfluenza vaccine/ or exp parainfluenza virus infection/ or para influenza virus.mp. or parainfluenza virus.mp. or parainfluenza viruses.mp. or Parainfluenzavirus.mp. or virus, parainfluenza.mp. or piv.mp. or exp Paramyxovirinae/
2. exp metapneumovirus/ or exp metapneumovirus infection/ or mpv.mp.
3. exp respiratory tract infection/ or exp pneumonia/ or exp bronchiolitis/ or exp viral bronchiolitis/
4. exp incidence/ or exp prevalence/ or exp morbidity/ or hospitalization rate.mp. or hospitalisation rate.mp. or exp hospital mortality/ or exp mortality/ or exp childhood mortality/ or exp infant mortality/ or exp death/ or exp child death/ or burden.mp./ or proportion.mp.
5. 1 and 3 and 4
6. 2 and 3 and 4
7. limit 5 to (human and (infant <to one year> or preschool child <1 to 6 years>))
8. limit 6 to (human and (infant <to one year> or preschool child <1 to 6 years>))
9. 7 or 8
10. limit 9 to yr="1995 -2019"

CINAHL
TI parainfluenza OR TI HPIV
TI metapneumovirus OR TI HMPV
AND
TI acute respiratory infection
AND
TI children
1995-2019

Global Health Library
(tw:(parainfluenza)) OR (tw:(piv)) OR (tw:(hpiv)) OR (tw:(metapneumovirus)) OR (tw:(mpv)) OR (tw:(hmpv)) AND (limit:("infant" OR "child, preschool" OR "child" OR "newborn"))
1995-2019

Web of Science
TITLE: (parainfluenza) OR TITLE: (HPIV) OR TITLE: (metapneumovirus) OR TITLE: (HMPV)
AND Title= (Acute Respiratory Infections) OR Title= (Pneumonia)
AND TOPIC: (children) OR TOPIC: (child) OR TOPIC: (infant)
1995-2019

Global Health (Ovid)
1. exp parainfluenza/ or exp parainfluenza viruses/ or exp human parainfluenza virus 1/ or exp human parainfluenza virus 2/ or exp human parainfluenza virus 3/ or exp human parainfluenza virus 4/ or piv.mp.
2. exp metapneumovirus/ or exp human metapneumovirus/ or metapneumovirus.mp.
3. exp respiratory diseases/ or exp bronchiolitis/ or exp lower respiratory tract infections/ or exp pneumonia/ or (respiratory diseases or lower respiratory tract infections).sh. or pneumonia.mp. or bronchiolitis.mp.
4. exp incidence/ or proportion.mp. or exp morbidity/ or hospitalization rate.mp. or hospitalisation rate.mp. or exp infant mortality/ or exp neonatal mortality/ or exp mortality/ or exp death/ or exp "causes of death"/
5. 1 or 2
6. 5 and 3 and 4
7. limit 6 to yr="1995 -2019"

CNKI
Topic: respiratory infections or pneumonia or bronchiolitis
And topic: parainfluenza virus or metapneumovirus
And topic: prevalence or deaths or incidence or disease burden or hospitalisation
And topic: children or infant
1995-2019

Wanfang
Topic: respiratory infections or pneumonia or bronchiolitis
And topic: parainfluenza virus or metapneumovirus
And topic: prevalence or deaths or incidence or disease burden or hospitalisation rate
And topic: children or infant
1995-2019

Chongqingvip
Any field: parainfluenza virus or metapneumovirus
AND title or key words: respiratory infection or respiratory tract infection or pneumonia or lung infection or severe pneumonia or bronchiolitis
AND title or key words: incidence or prevalence or death or hospitalisation or burden of disease
AND title or key words: children or infant.
1995-2019

Appendix 3. Characteristics of included studies

Table S3.1 Number of studies by age, region, and period for each outcome. [*]

	Incidence rate (N=11)[†]	Hospital admission rate (N=39)	Hospital admission rate of hMPV–ALRI with hypoxaemia (N=14)	Proportion of hospitalised hMPV–ALRI (N=115)	hCFR (N=73)
**From the collaboration network**	5	18	14	37	33
**0–59 m**	10	38	14	73	57
**Reporting by 0–5 m, 6–11 m, and 12–59 m**	6	29	14	63	28
**Developing countries**	7	28	14	91	60
**By World Bank income region**					
LIC	1	3	2	5	5
LMIC	5	11	5	26	25
UMIC	2	11	5	53	24
HIC	4	14	2	31	19
**By WHO region**					
AFR	1	12	5	16	17
AMR	3	11	1	12	13
EMR	1	1	1	11	11
EUR	0	5	1	17	7
SEAR	4	7	3	13	8
WPR	2	3	3	46	17
**By median study year**					
By 2005	1	5	2	12	11
2006–2010	4	12	0	31	23
2011 onward	5	22	12	72	39
**Number of hMPV–ALRI cases**					
0–99	9	24	NA	97	46
99–199	2	8	NA	14	10
200– ~	0	7	NA	4	10

[*] NA: not applicable.
[†] The number is the number of all studies identified in the review.

Table S3.2 Number of studies that were included in the main analysis by risk of bias for each outcome.[*]

	Incidence rate (N=10)[†]	Hospital admission rate (N=29)	Hospital admission rate of hMPV–ALRI with hypoxaemia (N=14)	Proportion of hospitalised hMPV–ALRI (N=73)	hCFR (N=28)
**Study design**	10	28	14	67	27
**Adjustment for healthcare utilization**	NA	22	9	NA	NA
**Patient groups excluded**	6	20	10	59	21
**Case definition**	9	22	13	43	23
**Sampling strategy**	7	23	10	54	20
**Test method**	10	26	14	67	NA
**Hypoxaemia ascertainment**	NA	NA	8	NA	NA

[*] NA: not applicable.
[†] The number refers to the number of studies included in the main analysis for each outcome. For incidence rates, all studies with data for children aged 0–59 months were included. For hospital admission rates and hCFRs, all studies with data for children aged 0–5 months, 6–11 months, and 12–59 months were included.

Appendix 4. Details of sensitivity analysis and summary of overall approaches used in this study

In Table S4.1, we estimated global hMPV-associated ALRI hospital admissions by summing up the estimates by World Bank income level and country development status where available.

In Table S4.2A and S4.2B, we estimated the hMPV-associated ALRI hospital admissions using the proportion-based approach. For the proportion-based approach, we applied the percent of hMPV in hospitalised ALRI to the global ALRI hospital admissions for 0-4 years. The estimates of all-cause ALRI hospital admissions for 2015-2016 ranged from 5,133,000 to 16,400,000 among children under five years, and were used in the proportion-based approach.[1,2]

In Table S4.3, we estimated global hMPV-associated ALRI mortality by summing up the estimates by World Bank income level and country development status where available.

In Table S4.4, we estimated the hMPV-associated ALRI in-hospital mortality for 2010 using available age-stratified hCFR data before 2010 (at least partly before 2010). The population estimate for 2010 was used.[3]

In Table S4.5, we estimated the number of hMPV-associated ALRI cases for 0-59 months in the community by World Bank income levels and country development status.

In Table S4.6, we estimated the number of hMPV-associated severe ALRI cases for 0-4 years in the community for high child mortality settings. We did not find any studies from low child mortality settings, so we were unable to estimate the number of hMPV-associated severe ALRI cases for this setting.

Figure S4.1 shows the overall approaches for hMPV burden estimation used in this study.

Table S4.1 Hospital admission rates (per 1,000 children per year) and hospital admissions for hMPV-associated ALRI by World Bank income levels and country development status*

Age		LMIC	UMIC	HIC	Global estimates by income	Developing	Industrialised	Global estimates by development status
**0-5 months (A)**	No. of studies	8	6	5		16	3	
	Hospital admission rate (/1,000)	2.4 (1.6-3.5)	3.3 (1.6-7.1)	3.3 (2.2-5.1)		2.7 (1.8-4.1)	3.4 (1.9-6)	
	Hospital admissions (*1,000)	97 (66-143)	55 (26-116)	19 (13-29)	171 (104-288)	153 (101-230)	22 (12-38)	174 (114-268)
**6-11 months (B)**	No. of studies	7	5	4		13	3	
	Hospital admission rate (/1,000)	2.7 (1.7-4.3)	2.5 (1.0-5.9)	2.8 (2.2-3.5)		2.5 (1.6-3.9)	2.5 (2.2-2.8)	
	Hospital admissions (*1,000)	129 (82-205)	50 (21-121)	19 (15-24)	199 (118-351)	168 (108-262)	19 (17-21)	187 (125-284)
**1-4 years (C)**	No. of studies	9	8	5		18	4	
	Hospital admission rate (/1,000)	0.6 (0.3-1)	0.4 (0.2-0.8)	0.3 (0.2-0.7)		0.5 (0.3-0.7)	0.4 (0.2-0.8)	
	Hospital admissions (*1,000)	206 (113-375)	59 (29-117)	15 (8-28)	280 (151-520)	242 (159-369)	22 (11-44)	264 (170-414)
**0-4 years (A+B+C)**	Hospital admissions (*1,000)	432 (260-723)	164 (77-354)	54 (36-82)	650 (373-1159)	563 (368-861)	63 (40-104)	626 (409-965)

* Hospital admission rates from meta-analysis. Global estimates were the sum of estimates by age and regions.

Table S4.2A. The proportion of hospitalised hMPV-associated ALRI for 0-4 years by World Bank income level.

	No. of studies	Proportion (%) for 0-4 y
**All studies for 0-4 years**	73	5.5 (4.9-6.4)
**By World Bank income group**		
Low income (L)	4	6.5 (5.3-7.9)
Middle income (M)	54	5.3 (4.4-6.3)
High income (H)	15	6.2 (4.5-8.5)

Table S4.2B. The hospital admissions of hMPV-associated ALRI for 0-4 years using the proportion-based approach

No. of studies	Proportion (%) for 0-4 y	Hospital admissions of all-cause ALRI	Hospital admissions of hMPV-associated ALRI
73	5.5	5,133,000– 16,400,000[1,2]	282,000 – 902,000

Table S4.3. The hCFRs (%) and in–hospital deaths of hMPV–associated ALRI by World Bank income levels and country development status*

Age		LMIC	UMIC	HIC	Global by income group	Developing	Industrialised	Global by country development status
	No. of studies	15	6	7		23	5	
0-5 months (A)	hCFR (%)	4.5 (2.3-8.6)	1.7 (0.6-5.1)	0.4 (0-8.6)		3.2 (1.7-6)	0.4 (0-8.5)	
	Deaths	4300 (2000-9200)	900 (300-3400)	100 (0-3100)	5400 (2300-15700)	4900 (2300-10200)	100 (0-3500)	5000 (2300-13700)
6-11 months (B)	hCFR (%)	0.7 (0-9)	..†	0.6 (0.1-3.9)		0.2 (0-7)	0.6 (0.1-4)	
	Deaths	900 (0-37800)	NA	100 (0-700)	1000 (0-38500)	300 (0-12400)	100 (0-700)	500 (0-13100)
1-4 years (C)	hCFR (%)	0.9 (0.3-2.8)	1.1 (0.1-9.1)	0.5 (0-7)		0.8 (0.2-3.6)	0.3 (0-3)	
	Deaths	1800 (500-6500)	600 (100-6600)	100 (0-2800)	2600 (600-15900)	1900 (400-8500)	100 (0-1700)	2000 (400-10300)
0-4 years (A+B+C)	Deaths	7200 (2600-52300)	1600 (300-10000)	300 (0-6600)	9100 (2900-68800)	7200 (2800-30500)	300 (0-5900)	7500 (2800-36800)

* hCFRs from meta-analysis. Global estimates were the sum of estimates by age and region.
† All studies reported no MPV-ALRI deaths for this stratum, so we were unable to compute hCFR and the number of deaths.

Table S4.4. The hCFRs (%) and in-hospital deaths of hMPV-associated ALRI for 2010 by the child mortality setting.*†

Age		Low child mortality setting	High child mortality setting	Global by child mortality setting
0-5 months (A)	No. of studies	6	4	
	hCFR (%)	0.9 (0.2-3.5)	2.9 (0.9-8.5)	
	Deaths	600 (100-2600)	6100 (1700-21200)	6600 (1800-23800)
6-11 months (B)	No. of studies	6	4	
	hCFR (%)	2.2 (0.5-8.2)	..‡	
	Deaths	1300 (300-5000)	..	1300 (300-5000)
1-4 years (C)	No. of studies	9	4	
	hCFR (%)	0.9 (0.3-2.8)	0.3 (0.0-2.4)	
	Deaths	900 (100-7400)	700 (0-15600)	1600 (100-22900)
0-4 years (A+B+C)	Deaths	2800 (500-15100)	6800 (1700-36800)	9600 (2300-51200)

* Only the hospital admission rate and hCFR data before 2010 (or partly before 2010) were included in this analysis. The population estimates for 2010 were used.

† hCFR from meta-analysis. Global estimates were the sum of estimates by age and region.

‡ All studies reported zero hMPV-associated ALRI death for this strata, so we were unable to compute hCFR and the number of deaths.

Table S4.5. The incidence and number of hMPV–associated ALRI cases for 0–59 months by World Bank income regions and country development status. [*]

	LMICs	UMICs	HICs	Global estimates by World Bank income levels	Developing	Industrialised	Global estimates by country development status
No. of studies	5 (1)	2 (1)	3 (2)		7 (2)	3 (2)	
Incidence rates (/1,000)	21.3 (15.5-29.3)	16.5 (8.3-32.8)	22.6 (13.4-38.2)		19.9 (14.6-27.1)	22.6 (13.4-38.2)	
Cases (*1,000)	9202 (6711-12620)	3019 (1521-5996)	1436 (854-2415)	13657 (9086-21031)	12127 (8926-16478)	1577 (938-2652)	13703 (9864-19130)

Table S4.6. The incidence and number of hMPV-associated severe ALRI cases for 2018 in high child mortality settings.

Age		High child mortality setting
0-5 months (A)	No. of studies	4
	Incidence rate (/1,000)	9.7 (1.5-58.1)
	Cases (*1,000)	446 (72-2,752)
6-11 months (B)	No. of studies	3
	Incidence rate (/1,000)	13.7 (3.5-51.6)
	Cases (*1,000)	625 (164-2,383)
1-4 years (C)	No. of studies	3
	Incidence rate (/1,000)	5.9 (2.2-15.8)
	Cases (*1,000)	2,104 (789-5,609)
0-4 years (A+B+C)	No. of studies	4 (1)
	Incidence rate (/1,000)	5.3 (1.6-17.9)
	Cases (*1,000)	2,375 (715-7,896)

[*] Incidence rates from meta-analysis. Global estimates were the sum of estimates by region.

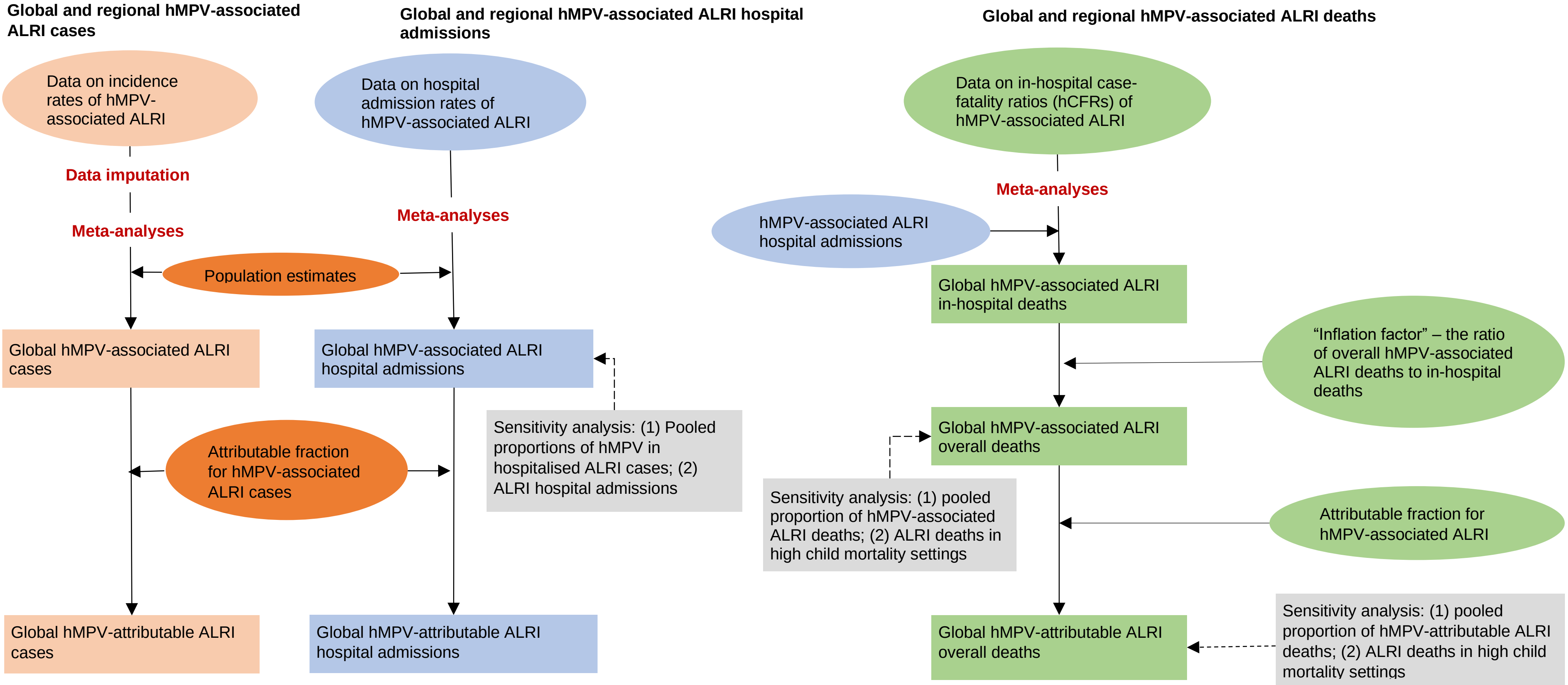

We report the global estimates of hMPV-associated ALRI cases, hMPV-associated ALRI hospital admissions, and hMPV-associated ALRI deaths in hospital and overall deaths (in-hospital and out-hospital). This figure summarises our approach for each outcome and also shows how they relate to each other. The oval shapes show input data and the square shapes show outputs. The solid lines show the main analyses and dashed lines show the sensitivity analyses. Global hospital admissions of hMPV–associated ALRI were estimated using an incidence-based approach in the main analysis (a proportion-based approach in the sensitivity analysis). hMPV–associated ALRI in–hospital deaths were estimated by combining hCFRs and hospital admissions of hMPV-associated ALRI. The overall mortality of hMPV–associated ALRI was estimated using the "inflation factor" approach in the main analysis. The inflation factor, defined as the ratio of overall hMPV-associated ALRI deaths to in-hospital deaths, was applied to the in-hospital mortality. In a sensitivity analysis, we estimated the overall hMPV-associated ALRI deaths by combining the proportion of hMPV-associated ALRI deaths and the total ALRI mortality for children aged 0-59 months. For hMPV-attributable morbidity and mortality burden, we applied the attributable fraction to the associated burden estimates in the main analysis. In a sensitivity analysis for hMPV-attributable mortality, we combined the proportion of hMPV-attributable ALRI death and the total number of ALRI deaths. hMPV: human metapneumovirus. ALRI: acute lower respiratory infection. hCFR: in-hospital case-fatality ratio.

Figure S4.1. Approaches for global human metapneumovirus–associated ALRI and human metapneumovirus–attributable morbidity and mortality estimation in children under five years

Appendix 5. Overall hMPV-associated ALRI mortality

(1) "Inflation factor" approach (main analysis)

The inflation factor of hMPV-associated ALRI in-hospital deaths was estimated by child mortality settings (Table S5.1). It is challenging to estimate the inflation factor for hMPV–associated ALRI deaths because the ALRI deaths occurring outside hospitals are rarely tested. Thus, we divided the overall pneumonia deaths by in-hospital pneumonia deaths, and used the median factor across sites as a proxy for the inflation factor for hMPV-specific ALRI deaths. For high child mortality settings, the details of the data for inflation factor estimation has been described previously.[4] For low child mortality settings, the inflation factor was estimated using the percent of children with pneumonia symptoms who received care at health providers as measured in Multiple Indicator Cluster Surveys, Demographic and Health Surveys, and other national surveys. The data were obtained from UNICEF.[5] In this analysis, the reciprocal of percent of children with pneumonia symptoms who sought care was estimated and used as a proxy for the inflation factor. The median inflation factor across regions and countries was applied to the in–hospital mortality estimate for low child mortality countries to yield the overall mortality of virus–associated ALRI deaths in that setting.

There are several factors that could affect the estimates of inflation factor and overall mortality. First, the paucity of data could affect the generalisation of the estimates, especially for high child mortality settings. Second, violations of assumptions could lead to bias in the estimates. For high child mortality settings, the estimates could have been biased if the location of childhood ALRI deaths (in–hospital versus other locations) is associated with the prevalence of hMPV in ALRI deaths. For low child mortality settings, estimation of inflation factor was based on one further assumption that CFR for hospitalised pneumonia cases was the same as CFR for pneumonia not hospitalised. The direction of bias related to this assumption could be complicated by the two–way association between disease severity and care–seeking.[6-8] For low child mortality settings, the estimate of inflation factor and overall mortality could likely have been underestimated because the definition of "care–seeking" in the UNICEF dataset is broader than the definition of "in–hospital": contact with primary care is included as "care–seeking" in surveys, but are not included in the "in–hospital mortality" estimates in the present analysis. The US vital statistics data show that about 40% of under–five ALRI deaths (ICD–10 J09–22; U04) occurred in outpatient or emergency departments during 2010–2017.[9] Moreover, the estimates of inflation factor could also be affected by accuracy of assessment and completeness of documentation for ALRI or pneumonia. For example, the diagnosis of (presumptive) pneumonia in the UNICEF dataset is based on caregivers' report, thus may be inaccurate and affect the estimates of inflation factor.

Table S5.1. Estimation of overall hMPV–associated ALRI mortality using the "inflation factor".

Setting	Site	Ratio of all pneumonia deaths over in–hospital deaths for 0–59 m (A)	Inflation factor (B=median of A)	In–hospital mortality of hMPV–associated ALRI (C)	Overall hMPV–associated ALRI mortality (D=B*C)
**High child mortality settings**	Nairobi, Kenya (urban), 2008, 2010-2015	1.7	2.2	6,800 (UR 2,500–27,100)	14,900 (UR 5,600–59,700)
	Siaya, Kenya (rural), 2011–2016	3.5			
	Nouna, Burkina Faso (rural), 2014–2016	1.5			
	Dodowa, Ghana (rural), 2011–2015	2.1			
	Manhiça, Mozambique (mixed), 2012–2016	2.8			
	Agincourt, South Africa (rural); 2010–2015	2.3			
	Mirzapur, Bangladesh (rural), 2008–2012 [10]	2.5			
	Multi–sites, Bangladesh (mixed), 2010–2012 [11*]	1.8			
**Low child mortality settings**	28 countries and regions [5]	Range from 1.1 to 4.5	1.3	800 (UR 100–22,200)	1,100 (UR 100–28,800)
**Global estimates**[†]					16,100 (UR 5,700–88,000)

[*] Including ARI deaths identified by community survey. ARI deaths were defined as for children under 5 years, sudden onset cough or difficulty in breathing within 2 weeks of death.
[†] Global estimates are the sum of estimates by child mortality setting.

Table S5.2. Proportion of children with pneumonia symptoms seeking care by country. [5]

Country	Data Source	Year	Care seeking (%)
Albania	DHS_2008-2009	2009	70
Argentina	MICS_2011-2012	2012	94
Armenia	DHS _2010	2010	57
Belarus	MICS_2012	2012	93
Belize	MICS_2011	2011	82
Bosnia and Herzegovina	MICS_2011-2012	2012	87
Brazil	MoH_PNDS_2006	2006	50
Colombia	DHS_2010	2010	64
Costa Rica	MICS_2011	2011	77
Cuba	MICS_2014	2014	93
El Salvador	MICS and MDG indicators(Prelim)_2014	2014	80
Georgia	MICS_2005	2005	74
Iran (Islamic Republic of)	IrMIDHS(Prelim)_2010-2011	2010	76
Jamaica	MICS_2011	2011	82
Jordan	DHS_2012	2012	77
Kazakhstan	MICS_2010-2011	2011	81
Lebanon	MICS_2000	2000	74
Maldives	MICS_2001	2001	22
Mongolia	MICS_2013-2014	2014	70
Montenegro	MICS_2005	2005	89
Panama	MICS(prelim)_2013	2013	82
Peru	DHS_2014	2014	60
Republic of Moldova	MICS_2012	2012	79
Serbia	MICS_2010	2010	90
Sri Lanka	DHS_2006-2007	2007	58
Thailand	MICS_2012	2012	83
The former Yugoslav Republic of Macedonia	MICS_2005-2006	2006	93
Ukraine	MICS_2012	2012	92

(2) Sensitivity analysis of hMPV-associated ALRI overall mortality in high child mortality settings using the proportion of hMPV-associated ALRI deaths

In sensitivity analysis, we estimated the overall hMPV-associated ALRI mortality for the high child mortality setting using the following formula:

$$\text{Overall hMPV} - \text{ALRI mortality} = \%hMPV\ in\ ALRI\ deaths * ALRI\ mortality$$

The proportion of hMPV positives (both mono– and co–infections) in ALRI deaths was estimated by combining the data from 13 hospital–based studies (including five PERCH sites) in which at least 90% of all–cause ALRI cases were tested, and at least five all–cause ALRI deaths were reported. In this analysis, we extrapolated the proportion of hMPV positives among ALRI deaths in hospital settings to community settings. About 19% of ALRI deaths in children under five years occur in the neonatal period.[12] However, there were no hMPV deaths among neonates in hospital–based studies. Therefore, to avoid overestimation we estimated the proportion for 1–59 months and applied the proportion to the all–cause ALRI mortality in children aged 1–59 months. As data were from hospital settings, we assumed that the location of childhood ALRI deaths (in–hospital versus other locations) was not associated with the prevalence of hMPV in ALRI deaths.

Table S5.2. Using the percent of hMPV-associated ALRI deaths based on hospital-based studies to estimate the overall hMPV-associated ALRI mortality for children aged 1-59 months in high child mortality settings.

	hMPV–ALRI deaths (A)	ALRI deaths (B)	% of hMPV in ALRI deaths (C)	2017 ALRI deaths for high child mortality settings (D)	Overall hMPV–ALRI deaths for high child mortality settings (E=C*D)
hMPV*†	18	573	3.2% (95%CI 1.9–5.2)	622,742 for 1–59 months	19,900 (UR 12,100–33,200) for 1–59 months

* A meta–analysis was conducted to combine data from 13 studies from Morocco, Philippines, Bangladesh, Gambia, Zambia, Mali, Kenya, South Africa, and Mozambique. The percent was 3.4% (95%CI 1.8–6.1) when pooling five PERCH sites in Gambia, Zambia, Mali, Kenya, and South Africa.
† The percent of hMPV was estimated for the 1–59 month age group, and only used to yield the hMPV–associated ALRI deaths for children aged 1–59 months.

Appendix 6. Estimating the attributable fraction for hMPV-associated ALRI deaths

(1) Estimating the attributable fraction (AF) for hMPV-associated ALRI deaths

As shown in Figure S6.1, we split the hCFR of hMPV–associated ALRI cases into two compartmental parts: hCFR of hMPV–attributable ALRI and hCFR of hMPV–unattributable ALRI. The AF for hMPV–associated ALRI mortality was modelled by assuming the hCFR for ALRI cases unattributed to hMPV was the same with the hCFR for hMPV–negative cases.

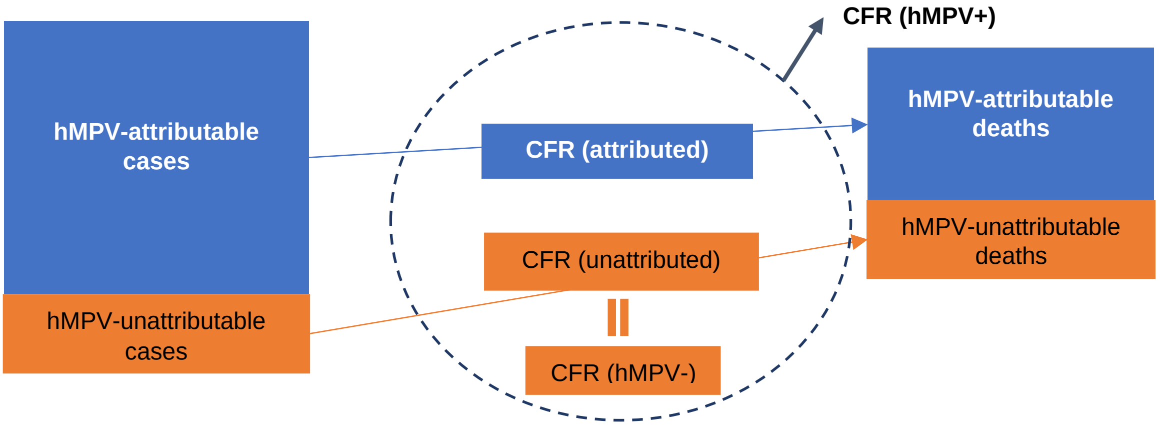

Figure S6.1. A schematic figure showing the estimation of attributable fraction for hMPV–associated ALRI deaths for children under five years.

The AF for hMPV-ALRI deaths was estimated based on the following formulas:

Formula A - $Deaths\ (hMPV+) = Cases(hMPV+) * hCFR(hMPV+)$

Formula B - $Deaths\ (hMPVattri) = [Cases(hMPV+) * \frac{AFcase(\%)}{100}] * hCFR(hMPVattri)$

The Deaths (hMPV+) and Cases (hMPV+) denote for the number of ALRI deaths and cases positive for hMPV; the hCFR (hMPV+) denote for the in-hospital case-fatality ratio for hMPV positive ALRI cases. Similarly, the Deaths (hMPVattri), Cases (hMPVattri), and hCFR (hMPVattri) denote for the measures for cases or deaths attributed to hMPV. The AFcase denotes for the AF (%) for hMPV-associated ALRI cases (we used 78% as the input - the median value of three pooled analyses of multi–country data).[13-15] Thus, based on Formula A and B, the AF (%) for hMPV-associated ALRI deaths could be estimated as follows:

Formula C - $AFdeaths(\%) = AFcase(\%) * \frac{hCFR(hMPVattri)}{hCFR(hMPV+)}$

Then we estimated the ratio of case-fatality of hMPV-attributable ALRI cases versus hMPV-positive cases following the strategy below. Results are in Table S6.1. The association between hCFR (hMPVattri), hCFR (hMPVnon-attri), and hCFR (hMPV+) is listed in Formula D and E:

Formula D - $Deaths\ (hMPV+) = Deaths\ (hMPVattri) + Deaths(hMPVnon-attri)$

$$= [Cases(hMPV+) * \frac{AFcase(\%)}{100}] * hCFR(hMPVattri) + [Cases(hMPV+) * \frac{100-AFcase(\%)}{100}] * hCFR(hMPVnon-attri)$$

Formula E (transformed from Formula D) -

$$hCFR\ (hMPV+) = \frac{AFcase(\%)}{100} * hCFR(hMPVattri) + \frac{100-AFcase(\%)}{100} * hCFR(hMPVnon-attri)$$

Table S6.1. Estimation of the attributable fraction for hMPV–associated ALRI deaths for 0–59 months.[*]

Study country and study period	hCFRs for hMPV–associated ALRI (%)	hCFRs for hMPV–negative ALRI (%)	hCFR meta-estimate for hMPV–associated ALRI (A)	hCFR meta-estimate for hMPV–negative ALRI (B)	Ratio of case–fatality for hMPV–unattributable to hMPV–positive ALRI (C=B/A)	Ratio of case–fatality for hMPV–attributable to hMPV–positive ALRI (D, estimated using A and C)[†]	AF for hMPV–associated ALRI deaths (=AF for hMPV–ALRI cases * D)[‡14,16,17]
**Morocco; 2010-2011**	4.3	3.8	3.2 (2.0–5.0)	4.5 (2.7–7.6)	1.4	0.9	70%
**Philippines; 2008-2015**	2.8	6.1					
**Philippines; 2012-2015**	0	4.9					
**Bangladesh; 2010-2014**	4.4	1.5					
**Gambia; 2012-2013**	2	2.8					
**Zambia; 2012-2013**	7.5	18.8					
**Mali; 2012-2014**	2.1	16.2					
**Kenya; 2011-2013**	3.6	4.9					
**South Africa; 2011-2013**	3.4	3.8					
**Philippines; 2014-2016**	0	5					
**Mozambique; 2011-2013**	3.6	1.3					
**Philippines; 2012-2015**	1.8	2.1					
**Philippines; 2012-2016**	0	2.6					

[*] Studies were eligible for the analysis if they tested ≥90% of cases and reported at least five ALRI deaths (to ensure the precision of estimates).

[†] Detailed formulas in Appendix 6.

[‡] The AF for hMPV–ALRI cases was calculated using odds ratios from one recent systematic review and two additional recent multi-country studies. The median estimate of odds ratio from the three studies was input to yield the attributable fraction for hMPV–ALRI cases (78%).

Appendix 7. Estimating hMPV-attributable ALRI mortality in high child mortality settings using CHAMPS data

CHAMPS investigates the causes of under-five mortality at seven sites in sub-Saharan Africa and South Asia, which are from high child mortality settings (Salzberg et al. 2019). Since the surveillance data are from high child mortality settings, the estimate was only used to yield hMPV-attributable ALRI mortality for high child mortality settings. For this analysis, we extracted the number of all-cause ALRI deaths and the number of hMPV-attributable ALRI deaths, where ALRI and hMPV could be anywhere in the causal pathway (including underlying cause or condition, immediate cause or condition, co-morbid causes or conditions) for the period December 2016 to October 2019.[18] The input data for this analysis and the results are in Table S7.1. Analysis was done separately for neonates and children aged 1-59 months.

Table S7.1. Estimating the hMPV–attributable ALRI deaths using CHAMPS data.

Age	hMPV–associated ALRI deaths (A) *†	ALRI deaths (B) ‡	% of hMPV–attributable ALRI (C=A*100/B)	2017 ALRI deaths for high child mortality settings (D)	HMPV–attributable ALRI deaths for high child mortality settings (E=C*D/100)
0–27 days	0	91	..	146,967	..
1–59 months	3	191	1.6 (0.3–3.5)	622,742	9,900 (UR 2,600–39,300)
0–59 months					..

In CHAMPS, the cause of ALRI deaths is ascertained based on the test result of post-mortem specimens and other individual information, including laboratory, histopathology and verbal autopsy, following a standardised procedure.[18,19] Although CHAMPS provides valuable data that improves the understanding of causes of ALRI deaths, several potential limitations should be noted as discussed in a recently published paper.[20] First, some deaths at the sites may be missed and the findings among captured deaths might differ from missed deaths. Second, the CHAMPS network includes seven sites in sub–Saharan Africa and South Asia, and the estimated viral attribution of these sites might not be generalizable to other regions and countries with high child mortality (e.g., the region of Americas). Third, although CHAMPS has conducted community–based and facility–based surveillance, the majority of the enrolled deaths during the initial phase of the surveillance were identified through health facilities. Fourth, CHAMPS provide site–combined data. Differences between sites could not be accounted for when analysing the combined dataset, thus the uncertainty of virus–attributable mortality estimates may have been underestimated.

* hMPV as any of immediate, co-morbid, and underlying cause of death (virus detected anywhere in the causal chain of deaths).

† Deaths occurring during Dec 2016-Oct 2019.

‡ ALRI as any of immediate, co-morbid, and underlying cause of death (ALRI occurred anywhere in the causal chain of deaths).

Appendix 8. Yearly variation in the hMPV-associated ALRI hospital admission rate

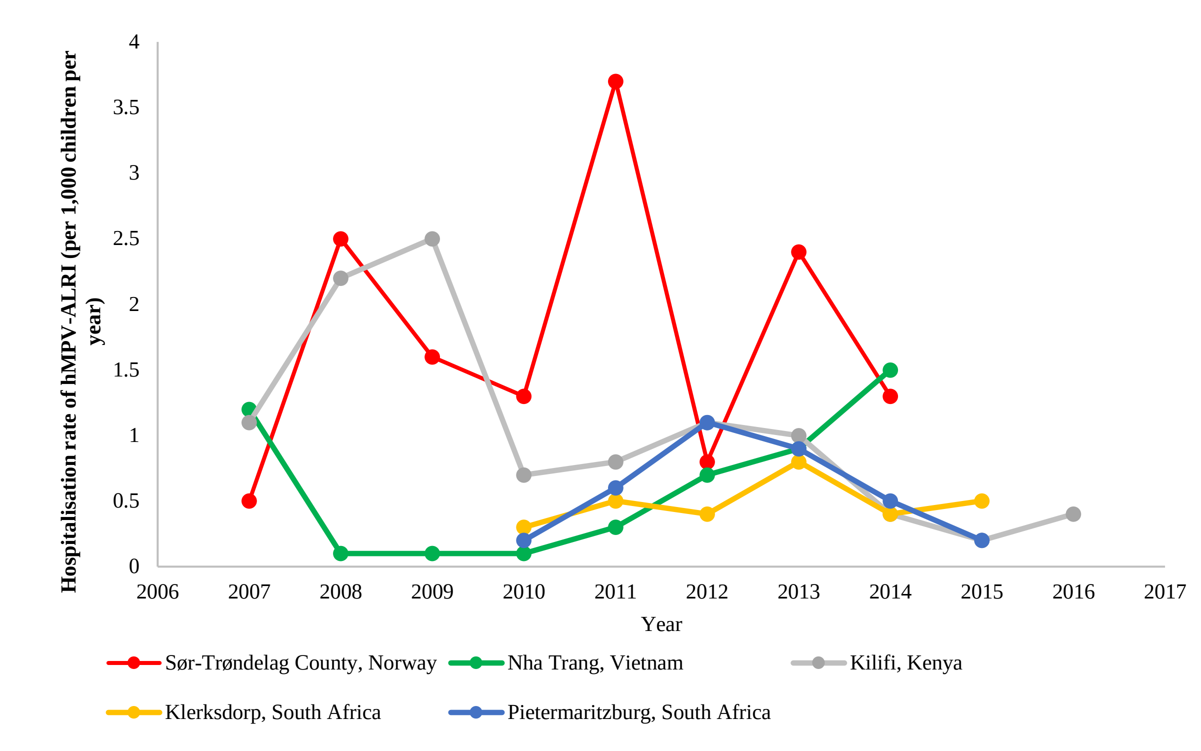

Figure S8.1. Yearly hospital admission rates of hMPV-associated ALRI in children under five years (with at least five years' data).

Table S8.1. The average hMPV-associated ALRI hospital admission rates in the above five studies for 0-59 months

ID	Location	Period	Average rate (per 1,000 children per year)
688	Sør-Trøndelag County, Norway	2007-2010	1.5
688	Sør-Trøndelag County, Norway	2011-2014	2.0
up_24	Nha Trang, Vietnam	2007-2010	0.3
up_24	Nha Trang, Vietnam	2011-2014	0.8
up_36	Kilifi, Kenya	2007-2010	1.6
up_36	Kilifi, Kenya	2011-2016	0.7
up_6	Klerksdorp, South Africa	2010-2012	0.4
up_6	Klerksdorp, South Africa	2013-2015	0.6
up_7	Pietermaritzburg, South Africa	2010-2012	0.7
up_7	Pietermaritzburg, South Africa	2013-2015	0.5

Appendix 9. Data imputation

Several studies reported data for 0–11 months, 0–23 months, and 0–35 months; to incorporate the information from these studies, the missing incidence rate for 0–59 months was imputed based on the available data in these age groups. The imputation was done at the study level following three steps: (1) imputing the denominator; (2) imputing the rate; (3) calculating the case number using the denominator and rate. Steps (2) and (3) were skipped if the case number was available. The reference group referred to the age group with available rate data and could be one of 0–35 months, 0–23 months or 0–11 months. When two or more age groups were available, the reference group was chosen in the following order: 0–35 months, 0–23 months, then 0–11 months.

We imputed data using the same approach as used previously for influenza burden estimation.[4] Details of each step of imputation were:

(1) The denominator was imputed by country income regions based on the probability of dying between age n and n+x (nqx) for both sexes in 2013, obtained from WHO life tables (World Health Organization, 2017). The proportion of total under–five population that are in the reference age group was calculated using the nqx estimates (World Health Organization, 2017). Using this proportion and the denominator in the reference group, the denominator for 0–59 months was estimated. Since the nqx is only available for 0–11 months and 12–59 months, one assumption is that the probability of dying is the same from 12 to 59 months (this assumption is only required when using 0–23 months and 0–35 months as the reference group). Another assumption is that the structure of the population under five years for each site is similar to the population structure for the corresponding country income region.

(2) The case number was imputed using a multiple imputation approach assuming the rates for 0–59 months were missing at random.[21] Figure 3–3 shows the process. First, meta–analysis was performed to estimate the rate ratios between 0–59 months and any of 0–11 months, 0–23 months, and 0–35 months (meta–analysis was only done when there were three or more studies). Second, the pooled rate ratio was assumed to follow a log–normal distribution, and 100 samples of rate ratios were simulated. Third, 100 samples of rates for 0–59 months were generated based on the rate in the reference group and the corresponding rate ratios. Fourth, case numbers were calculated using the denominator and imputed rates. Using the method, 100 datasets of imputed case numbers were generated. Fifth, meta–analysis was done for each dataset, and the meta–estimates were combined together using the Rubin's rules.[22,23]

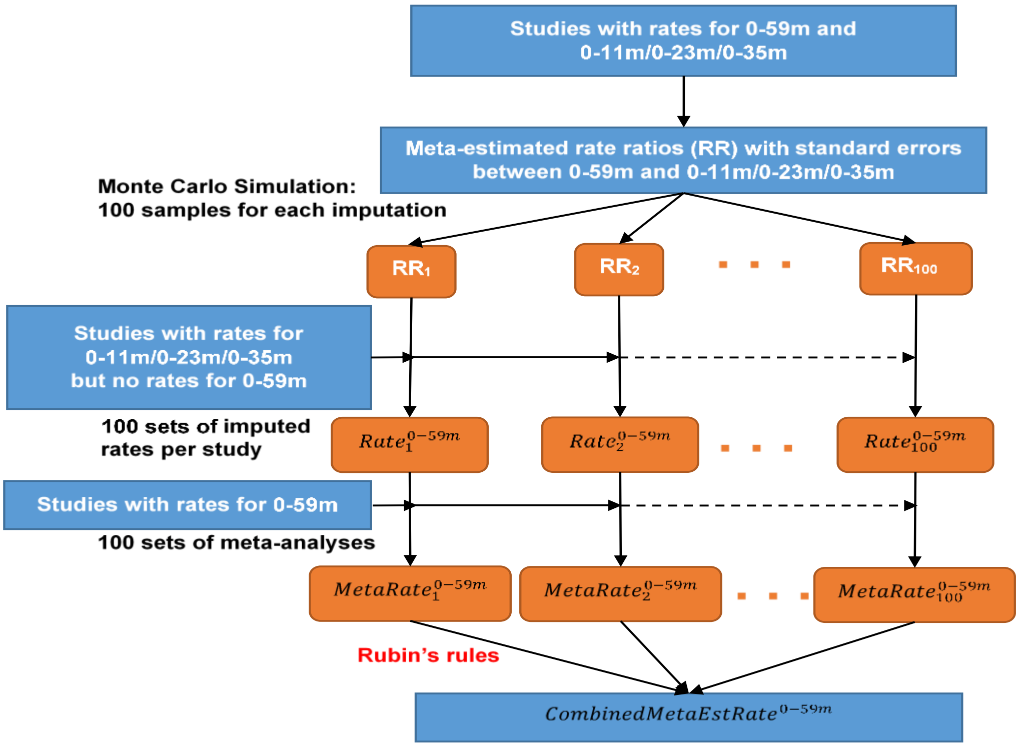

Figure S9.1. Imputing missing rates for 0–59 months using the multiple imputation approach.

Table S9.1. Pooled incidence rates of hMPV–associated ALRI for 0–59 months including and excluding imputed data*

	Pooled rates with imputed data		Pooled rates without imputed data	
	**No of studies (No. of imputed studies)**	**Rate**	**No of studies**	**Rate**
Low child mortality	4 (3)	18.9 (11.2-31.9)	1	17.5†
High child mortality	6 (1)	22.1 (17.0-28.7)	5	24.5 (20.2-29.6)

* Using multiple imputation method.
† Only one study reported data for 0-59 months in low child mortality settings.

Appendix 10. Details of denominator scaling

Before meta-analyses, we scaled the population-at-risk by applying the original denominator to the proportion of eligible cases who are tested at study levels. In published studies to prevent underestimating rates, the number of cases are usually scaled (the observed case number divided by the proportion of eligible cases who are tested) instead of the denominator. The rates would be the same using the two scaling strategies as in Formulas 1 and 2. However, we considered the scaled denominator could better reflect the true size of each study than the original denominator of each study, thus the weight of each study in meta-analyses.

Formula 1 - scaling the case number:

$$\text{Rate} = (\text{No. of observed cases}/\text{Proportion of cases tested})/\text{Original denominator}$$

Formula 2 - scaling the denominator:

$$\text{Rate} = \text{No. of observed cases}/(\text{Proportion of cases tested} * \text{Original denominator})$$

In Formula 1, the denominator used in meta-analyses would be the original denominator, while in Formula 2, the denominator is scaled (Proportion of cases tested * Original denominator). We estimated the hMPV-associated ALRI hospital admissions using Formula 1 (Table S8.1); the hospital admissions were very similar with those in the main analysis.

Table S10.1. Hospital admission rates of hMPV-ALRI and hospital admissions adjusting for the proportion of testing in the case number.

Age		LMIC	UMIC	HIC	Global
0-5 m (A)	No. of studies	8	6	5	
	Hospital admission rate (/1,000)	2.4 (1.6-3.6)	3.2 (1.6-6.6)	3.3 (2.2-5)	
	Hospital admissions (*1,000)	97 (65-145)	54 (27-109)	19 (13-29)	169 (104-282)
6-11 m (B)	No. of studies	7	5	4	
	Hospital admission rate (/1,000)	2.7 (1.7-4.2)	2.4 (1-5.9)	2.7 (2.2-3.4)	
	Hospital admissions (*1,000)	129 (83-203)	48 (20-117)	19 (15-23)	196 (118-343)
1-4 y (C)	No. of studies	9	8	5	
	Hospital admission rate (/1,000)	0.6 (0.3-1)	0.4 (0.2-0.8)	0.3 (0.2-0.7)	280 (151-520)
	Hospital admissions (*1,000)	206 (113-375)	59 (29-117)	15 (8-28)	
0-4 y (A+B+C)	Hospital admissions (*1,000)	432 (260-722)	160 (76-342)	53 (36-80)	645 (372-1144)

For hCFR estimation, we only used the information from the cases that were tested, and did not adjust for under-detection. The hCFR of tested ALRI cases was higher than the hCFR of untested ALRI cases (Table S10.2).

Table S10.2. The hCFR for tested ALRI cases and the hCFR for untested ALRI cases for 0-4 years using available data.*

ID	Location	Year	Tested cases (A1)	Tested deaths (A2)	Untested cases (B1)	Untested deaths (B2)	hCFRs for tested (A=A2/A1, %)	hCFR for untested (C=B2/B1, %)
up_17	Lusaka, Zambia	2012-2013	590	105	16	9	17.8	56.3
up_18	Bamako, Mali	2012-2014	659	100	1	0	15.2	0
up_19	Kilifi, Kenya	2011-2013	566	27	2	2	4.8	100
up_20	Soweto, South Africa	2011-2013	866	33	8	0	3.8	0
up_26	Manhiça, Mozambique	2011-2013	477	6	15	7	1.3	46.7
up_33	Soweto, South Africa	2000-2002	1409	66	235	25	4.7	10.6
up_36	Kilifi, Kenya	2007-2011; 2013-2016	2758	93	803	79	3.4	6.2
up_5	Berlin, Germany	2010-2014	2516	9	9	0	0.4	0
up_6	Klerksdorp, South Africa	2010-2015	1259	31	45	2	2.5	4.4
up_7	Pietermaritzburg, South Africa	2010-2015	2164	18	52	1	0.8	1.9
up_8	Aurora, Colorado, USA	2010-2016	6424	60	9261	18	0.9	0.2
up_16	Basse, Gambia	2012-2013	623	17	12	5	2.7	41.7
Meta-estimates							2.8 (1.5-5.3)	8.0 (1.9-27.7)

* Studies with small number of ALRI deaths (<5 ALRI deaths) were excluded in this analysis. The hCFRs in these studies were very imprecise. Also, very few hMPV-associated deaths would be missed due to under-detection.

Appendix 11. Assessment tool for risk of bias in individual studies

Table S11.1 Assessment of risk of biases for community-based studies

Category	Description	Risk of bias
Study design	Studies where the cases were prospectively enrolled	Low
	Other studies	High
Patient groups excluded	No exclusions that may affect estimates	Low
	Exclusions that may affect estimates, e.g., any of the following:<br>1. Not including very young children (e.g., neonates).<br>2. Excluding children with high-risk conditions.<br>3. Other exclusions that may affect estimates	High
Case definition	Using common/standard definitions	Low
	Using non-standard/inconsistent definitions	High
Sampling strategy	The proportion of testing is available AND either of the following:<br>1. ≥90% of eligible cases have been tested.<br>2. Testing a systematic sample of patients.	Low
	<90% of eligible cases have been tested<br>OR<br>The proportion of eligible cases who have been tested is unavailable.	High
Diagnostic test	PCR;<br>Or using other diagnostic tests, but confirming negative samples with PCR	Low
	1. Other diagnostic tests, e.g., culture, IFA, DFA.<br>2. No mention of diagnostic tests	High

Table S11.2 Assessment of risk of biases for hospital-based studies

Category	Description	Risk of bias
Study design	Studies where the cases were prospectively enrolled	Low
	Other study designs	High
Adjustment for healthcare utilization (only for hospital admission rate studies)	Meeting either of the following:<br>1. Including all or main hospitals;<br>2. Not including main hospitals, but adjusting for the proportion of patients admitted in the study hospitals	Low
	Not including main hospitals OR no related description; AND no adjustment for the proportion of patients admitted in the study hospitals	High
Patient groups excluded	No exclusions that may affect estimates	Low
	Exclusions that may affect estimates, e.g., any of the following:<br>1. Not including very young children (e.g., neonates).<br>2. Excluding children with high-risk conditions.<br>3. Other exclusions that may affect estimates	High
Case definition	Using common/standard definitions	Low
	Using non-standard/inconsistent definitions	High
Sampling strategy	The proportion of testing is available AND either of the following:<br>1. ≥90% of eligible patients have been tested.<br>2. Testing a systematic sample of patients.	Low
	<90% of eligible cases have been tested<br>OR<br>The proportion of eligible cases who have been tested is unavailable.	High
Diagnostic test (only for hospital admission rate studies)	PCR;<br>Or using other diagnostic tests, but confirming negative samples with PCR or culture	Low
	1. Other diagnostic tests, e.g., culture, IFA, DFA.<br>2. No mention of diagnostic tests	High
Hypoxaemia ascertainment (only for studies providing hypoxaemia data)	SpO2 was recorded for all human metapneumovirus-confirmed cases	Low
	1. SpO2 was recorded for a proportion of human metapneumovirus-confirmed cases.<br>2. No mention of how many human metapneumovirus-confirmed cases have been assessed for hypoxaemia.	High

Appendix 12. Risk of bias in individual studies

Table S12.1 Risk of bias for community-based studies reporting incidence rates of hMPV-associated ALRI*

ID	Location; period	Study Design	Patient groups excluded	Case definition	Sampling strategy	Test method
742	USA; Jan-Dec 2009	Low	Low	Low	High	Low
up_25	South Africa; Jun 2012-Dec 2017	Low	Low	Low	Low	Low
up_27	Pakistan; Dec 2012-Nov 2013	Low	Low	Low	Low	Low
up_3	Bangladesh; 2013-2014	Low	Low	Low	High	Low
up_39	Nepal; 2004-2007	Low	High	Low	Low	Low
up_4	India; Aug 2012-Aug 2014	Low	Low	Low	Low	Low
388	USA; 1976-2001	Low	High	Low	High	Low
395	Peru; Mar 2009 –Sep 2011	Low	Low	High	Low	Low
735	Australia; Sep 2010-Oct 2014	Low	High	Low	High	Low
737	Australia; Jul 1996-Jul 1999	Low	High	Low	Low	Low
869	Bangladesh; 2004-2008	Low	Low	Low	Low	Low

Table S12.2 Risk of bias for community-based studies reporting incidence rates of hMPV-associated chest wall indrawing ALRI.

ID	Location; period	Study Design	Patient groups excluded	Case definition	Sampling strategy	Test method
12	Pakistan; Oct 2011–July 2014	Low	Low	Low	Low	Low
up_25	South Africa; Jun 2012-Dec 2017	Low	Low	Low	Low	Low
up_3	Bangladesh; 2013-2014	Low	Low	Low	High	Low
up_39	Nepal; 2004-2007	Low	High	Low	Low	Low
up_4	India; Aug 2012-Aug 2014	Low	Low	Low	Low	Low

* Low for a low risk of bias, and high for a high risk of bias.

Table S12.3 Risk of bias for hospital-based studies reporting hospital admission rate of hMPV-associated ALRI [*]

ID	Location; period	Study Design	Adjustment for healthcare utilization	Patient groups excluded	Case definition	Sampling strategy	Test method
107	USA; Nov-May, 2003-2009	Low	Low	High	High	Low	Low
147	South Africa; Feb 2009-Dec 2012	Low	Low	High	Low	Low	Low
158	Thailand; 2005-2010	Low	Low	Low	High	Low	High
174	USA; Jan 2010-June 2012	Low	Low	High	Low	Low	High
264	USA; Aug 2000-Sep 2001	Low	Low	High	High	Low	Low
270	United Kingdom; Oct 2001-June 2002	Low	Low	Low	High	High	Low
460	USA; July 2007-June 2013	High	Low	Low	Low	High	High
51	India; Aug 2009-July 2011	Low	Low	Low	High	Low	Low
552	Guatemala; Nov 2007-Dec 2012	Low	Low	Low	Low	Low	Low
6	Kenya; Sep 2007-Aug 2010	Low	Low	Low	Low	Low	Low
742	USA; Jan-Dec 2009	Low	Low	Low	Low	High	Low
85	Spain; July 2004-June 2007	Low	Low	Low	High	Low	Low
up_13	Thailand; Jan 2012-Dec 2013	Low	High	High	Low	Low	Low
up_14	Thailand; Jan 2012-Dec 2013	Low	High	High	Low	Low	Low
up_23	Philippines; Feb 2014-Jun 2016	Low	Low	Low	Low	Low	Low
up_24	Viet Nam; Jan 2007-Dec 2014	Low	High	Low	Low	Low	Low
up_25	South Africa; Jun 2012-Dec 2017	Low	Low	Low	Low	Low	Low
up_26	Mozambique; Jan-Dec 2011	Low	Low	Low	Low	Low	Low
up_28	Chile; 2012-2013	Low	Low	Low	Low	Low	High
up_3	Bangladesh; 2013-2014	Low	Low	Low	Low	High	Low
up_30	Argentina; Jun 2008-Dec 2010	Low	Low	High	High	Low	Low
up_31	Philippines; Jul 2000-Dec 2004	Low	Low	High	Low	High	Low
up_32	South Africa; 2015-2017	Low	Low	Low	Low	High	Low
up_33	South Africa; Jan 2000-Dec 2002	Low	Low	Low	Low	High	Low
up_34	India; May 2009-Apr 2013	Low	Low	Low	Low	Low	Low
up_35	Jordan; Mar 2010-Mar 2013	Low	Low	Low	Low	Low	Low
up_36	Kenya; Jan 2007- Dec 2017	Low	Low	Low	Low	Low	Low
up_38	Argentina; May 2011-Aug 2013	Low	Low	Low	Low	Low	Low
up_6	South Africa; 2010-2015	Low	High	Low	Low	Low	Low
up_7	South Africa; 2010-2015	Low	High	Low	Low	Low	Low
389	USA; Oct 2001- Sep 2003	Low	Low	High	High	Low	Low
395	Peru; Mar 2009 –Sep 2011	Low	Low	Low	High	Low	Low
688	Norway; Nov 2006-July 2015	Low	Low	High	Low	High	Low
738	Mozambique; Sep 2006-Sep 2007	Low	Low	Low	Low	Low	Low
739	Kenya; Mar 2007-Feb 2010	Low	Low	Low	Low	High	Low
up_12	Bangladesh; 2010-2014	Low	Low	Low	High	Low	Low

[*] Low for a low risk of bias, and high for a high risk of bias.

Table S12.4 Risk of bias for hospital-based studies reporting proportions of hMPV-associated ALRI[*]

ID	Location; period	Study Design	Patient groups excluded	Case definition	Sampling strategy	Test method
104	Brazil; March 2008-Feb 2010	Low	Low	High	Low	Low
151	Brazil; April 2012-March 2013	Low	Low	Low	High	Low
158	Thailand; 2005-2010	Low	Low	High	Low	High
164	United Kingdom; Oct 2004-Oct 2005	High	Low	High	High	Low
167	France; Oct 2007-Sep 2008	High	Low	High	High	Low
173	India; May 2011–April 2013	Low	Low	Low	Low	Low
189	Cameroon; Sep 2011-Sep 2013	Low	Low	High	Low	Low
190	Oman; Dec 2007-Dec 2008	Low	High	High	Low	Low
223	China; Jan 2011-Dec 2013	High	Low	High	Low	Low
230	China; July 2008-June 2010	High	Low	Low	High	Low
238	Italy; Jan 2000-May 2002	Low	Low	High	High	Low
275	Brazil; 2003-2006	Low	Low	High	High	Low
329	USA; Oct 2005-Sep 2007	Low	Low	High	High	Low
383	Mexico; Oct 2010-Sep 2014	Low	Low	High	Low	Low
389	USA; Oct 2001- Sep 2003	Low	High	High	Low	Low
391	Israel; Nov 2001-Oct 2005	Low	Low	Low	Low	Low
398	Greece; Oct 1999-Sep 2000	Low	Low	High	High	Low
403	China; Dec 2011-Nov 2012	Low	Low	Low	Low	Low
410	Italy; 2004-2008	High	Low	Low	Low	Low
450	China; Sep 2008-Aug 2009	High	Low	Low	Low	High
463	Viet Nam; May 2009-Dec 2010	Low	High	High	Low	Low
469	Republic of Korea; Sep 2011-Aug 2012	High	Low	High	High	Low
488	Japan; April 2007-March 2012	Low	Low	Low	High	Low
503	China; Jan-Dec 2011	Low	Low	High	Low	Low
51	India; Aug 2009-July 2011	Low	Low	High	Low	Low
530	China; July 2009-June 2014	Low	Low	High	High	Low
534	China; March 2010-Feb 2012	High	Low	High	Low	Low
550	India; April 2010-March 2011	Low	Low	Low	Low	Low
556	Australia; Jan 2000-Dec 2005	High	Low	Low	Low	Low
57	Spain; Sep 2005-Aug 2008	Low	Low	High	High	Low
570	China; Jan-Dec 2007	High	Low	Low	Low	Low
573	Poland; Oct 2008-April 2011	High	Low	High	High	Low
593	Spain; Jan-Dec 2011	Low	High	High	Low	High
60	Italy; Oct 2004-Sep 2006	Low	Low	High	Low	Low
641	China; Jan-Dec 2011	Low	High	Low	Low	High
660	China; Sep 2007-Aug 2008	Low	Low	Low	High	Low
675	China; Feb 2011-Jan 2012	Low	Low	Low	High	Low
695	Bangladesh; Aug 2014-Jul 2015	Low	High	High	High	Low
730	Spain; Jan 2011-Jan 2013	Low	Low	Low	High	Low
731	United Kingdom; 2009-2012	Low	High	Low	High	Low
733	Cyprus; Nov 2010-Oct 2013	Low	Low	High	High	Low

[*] Low for a low risk of bias, and high for a high risk of bias.

ID	Location; period	Study Design	Patient groups excluded	Case definition	Sampling strategy	Test method
734	Iraq; Apr 2011-Mar 2012	Low	Low	High	Low	Low
738	Mozambique; Sep 2006-Sep 2007	Low	Low	Low	Low	Low
739	Kenya; Mar 2007-Feb 2010	Low	Low	Low	High	Low
74	China; April 2006-March 2008	Low	Low	Low	Low	Low
740	China; 2007-2010	Low	Low	High	Low	Low
741	Brazil; Apr 2008-Mar 2009	Low	Low	High	Low	Low
742	USA; Jan-Dec 2009	Low	Low	Low	High	Low
744	South Africa; 2003-2004	High	Low	High	High	Low
745	China; Mar 2010-Feb 2011	Low	Low	High	Low	Low
85	Spain; July 2004-June 2007	Low	Low	High	Low	Low
123	France; 2003-2004	Low	Low	High	High	Low
736A	Egypt; 2007-2014	Low	Low	High	High	Low
736B	Jordan; 2008-2010	Low	Low	High	High	Low
736C	Oman; 2008-2009	Low	Low	High	High	Low
736D	Qatar; 2008-2009	Low	Low	High	High	Low
736E	Yemen; 2010-2014	Low	Low	High	High	Low
c100	China; Apr 2012-Mar 2013	Low	Low	Low	Low	Low
c138	China; Apr 2008-Mar 2009	Low	Low	High	Low	Low
c14	China; Jan 2011-Dec 2012	High	Low	High	Low	Low
c192	China; Jan-Dec 2010	High	Low	High	Low	High
c276	China; Oct 2010-Sep 2012	High	High	High	Low	Low
c283	China; Jul 2013-Jun 2014	Low	Low	High	Low	Low
c287	China; Jan-Dec 2014	Low	High	Low	Low	High
c293	China; Jan-Dec 2015	High	Low	High	Low	Low
c298	China; Oct 2011-Sep 2012	Low	Low	High	Low	High
c303	China; Jul 2012-Jul 2013	Low	Low	Low	Low	Low
c309	China; Mar 2010-Feb 2011	Low	Low	Low	Low	Low
c36	China; Apr 2013-Mar 2014	Low	Low	Low	Low	Low
c44	China; Jun 2009-May 2012	Low	Low	High	Low	Low
c70	China; Jan 2011-Dec 2013	Low	Low	Low	Low	Low
769	China; 2016-2017	Low	Low	Low	Low	Low
785	China; 2012-2015	Low	Low	Low	Low	Low
786	China; 2013-2016	Low	Low	Low	Low	Low
C362	China; 2016-2017	Low	High	High	Low	Low
827	China; 2014-2016	Low	High	Low	Low	Low
831	China; 2008-2014	Low	Low	Low	High	Low
833	China; 2006-2015	Low	Low	High	High	High
838	China; 2017-2018	Low	Low	Low	Low	Low
up_1	Morocco; Nov 2010-Dec 2011	Low	Low	Low	Low	Low
up_10	Philippines; May 2008-Feb 2015	Low	Low	Low	Low	Low
up_11	Philippines; Sep 2012-Feb 2015	Low	Low	Low	Low	Low
up_12	Bangladesh; 2010-2014	Low	Low	High	Low	Low
up_13	Thailand; Jan 2012-Dec 2013	Low	High	Low	Low	Low
up_14	Thailand; Jan 2012-Dec 2013	Low	High	Low	Low	Low

ID	Location; period	Study Design	Patient groups excluded	Case definition	Sampling strategy	Test method
up_15	Iran (Islamic Republic of); Mar 2010- Mar 2013	High	Low	Low	High	Low
up_16	Gambia; 2012-2013	Low	High	Low	Low	Low
up_17	Zambia; Oct 2011 - Oct 2014	Low	High	Low	Low	Low
up_18	Mali; Jan 2012 - Jan 2014	Low	High	Low	Low	Low
up_19	Kenya; Aug 2011-Jul 2013	Low	High	Low	Low	Low
up_2	Pakistan; Aug 2009-Jul 2012	Low	Low	High	Low	Low
up_20	South Africa; Aug 2011-Aug 2013	Low	Low	Low	Low	Low
up_21	Bangladesh; Jan 2012 - Dec 2013	Low	High	Low	Low	Low
up_22	Bangladesh; Jan 2012-Dec 2013	Low	High	Low	Low	Low
up_23	Philippines; Feb 2014-Jun 2016	Low	Low	Low	Low	Low
up_24	Viet Nam; Jan 2007-Dec 2014	Low	Low	Low	Low	Low
up_25	South Africa; Jun 2012-Dec 2017	Low	Low	Low	Low	Low
up_26	Mozambique; Jan-Dec 2011	Low	Low	Low	Low	Low
up_28	Chile; 2012-2013	Low	Low	Low	Low	High
up_29	Philippines; Aug 2012-Feb 2015	Low	Low	Low	Low	Low
up_3	Bangladesh; 2013-2014	Low	Low	Low	High	Low
up_30	Argentina; Jun 2008-Dec 2010	Low	High	High	Low	Low
up_31	Philippines; Jul 2000-Dec 2004	Low	High	Low	High	Low
up_32	South Africa; 2015-2017	Low	Low	Low	High	Low
up_33	South Africa; Jan 2000-Dec 2002	Low	Low	Low	High	Low
up_34	India; May 2009-Apr 2013	Low	Low	Low	Low	Low
up_35	Jordan; Mar 2010-Mar 2013	Low	Low	Low	Low	Low
up_36	Kenya; Jan 2007- Dec 2017	Low	Low	Low	Low	Low
up_37	Spain; 2014-2017	Low	Low	High	High	Low
up_38	Argentina; May 2011-Aug 2013	Low	Low	Low	Low	Low
up_40	Nepal; Jan 2006-Jan 2008	Low	High	Low	Low	Low
up_5	Germany; Jan 2010-Dec 2014	Low	Low	Low	Low	Low
up_6	South Africa; 2010-2015	Low	Low	Low	Low	Low
up_7	South Africa; 2010-2015	Low	Low	Low	Low	Low
up_8	USA; 2010-2016	Low	Low	Low	High	High
up_9	Philippines; Sep 2012-Jul 2016	Low	Low	Low	Low	Low

Table S12.5. Risk of bias for hospital-based studies reporting in-hospital case-fatality ratios (hCFRs) of hMPV-associated ALRI*

ID	Location; period	Study Design	Patient groups excluded	Case definition	Sampling strategy
102	Norway; Nov 2002-April 2003	High	Low	High	High
104	Brazil; March 2008-Feb 2010	Low	Low	High	Low
135	Spain; Oct 2000-June 2003	Low	High	High	Low
144	USA; Oct 2001-May 2004	High	Low	High	High
148	Cambodia; April 2007-Feb 2010	Low	High	Low	High
152	USA; June 2007-June 2010	High	Low	Low	High
169	South Africa; June-August 2002	Low	Low	High	Low
31	Pakistan; March 2011-April 2012	Low	High	Low	Low
329	USA; Oct 2005-Sep 2007	Low	Low	High	High
351	Thailand; April 2002-Aug 2004	Low	High	High	High
389	USA; Oct 2001- Sep 2003	Low	High	High	Low
426	Saudi Arabia; July 2007-Nov 2008	High	Low	High	High
435	Turkey; Nov 2011-May 2012	High	Low	High	High
438	Mali; July 2011-Dec 2012	Low	High	Low	Low
459	South Africa; Jan 2010-Dec 2013	Low	Low	Low	Low
460	USA; July 2007-June 2013	High	Low	Low	High
462	Viet Nam; Nov 2004-Jan 2008	Low	High	High	Low
492	Turkey; Oct 2006-March 2007	Low	High	High	Low
51	India; Aug 2009-July 2011	Low	Low	High	Low
515	Jordan; Dec 2003-May 2004	Low	Low	High	High
540	Chile; May 2005-May 2007	Low	High	Low	High
552	Guatemala; Nov 2007-Dec 2012	Low	Low	Low	Low
577	Argentina; Nov-Dec 2009	High	Low	High	High
616	Japan; 37712	High	Low	Low	High
645	China; Jan 2006-Dec 2007	High	Low	High	Low
689	Norway; Nov 2006-July 2015	Low	Low	Low	High
736	Egypt, Jordan, Oman, Qatar and Yemen; Dec 2007-Feb 2014	Low	Low	High	High
738	Mozambique; Sep 2006-Sep 2007	Low	Low	Low	Low
741	Brazil; Apr 2008-Mar 2009	Low	Low	High	Low
82	Republic of Korea; Dec 2003-Feb 2005	Low	Low	High	High
85	Spain; July 2004-June 2007	Low	Low	High	Low
c149	China; June 2006-June 2007	High	Low	Low	Low
c157	China; Sep 2007-Aug 2008	Low	Low	Low	Low
c237	China; Jan 2009-Dec 2012	High	High	High	Low
c272	China; Jan 2006-Dec 2008	Low	High	High	Low
c306	China; Dec 2006-Feb 2008	Low	Low	Low	High
up_1	Morocco; Nov 2010-Dec 2011	Low	Low	Low	Low
up_10	Philippines; May 2008-Feb 2015	Low	Low	Low	Low
up_11	Philippines; Sep 2012-Feb 2015	Low	Low	Low	Low

* Low for a low risk of bias, and high for a high risk of bias.

ID	Location; period	Study Design	Patient groups excluded	Case definition	Sampling strategy
up_12	Bangladesh; 2010-2014	Low	Low	High	Low
up_13	Thailand; Jan 2012-Dec 2013	Low	High	Low	Low
up_14	Thailand; Jan 2012-Dec 2013	Low	High	Low	Low
up_17	Zambia; Oct 2011 - Oct 2014	Low	High	Low	Low
up_18	Mali; Jan 2012 - Jan 2014	Low	High	Low	Low
up_19	Kenya; Aug 2011-Jul 2013	Low	High	Low	Low
up_2	Pakistan; Aug 2009-Jul 2012	Low	Low	High	Low
up_20	South Africa; Aug 2011-Aug 2013	Low	Low	Low	Low
up_21	Bangladesh; Jan 2012 - Dec 2013	Low	High	Low	Low
up_22	Bangladesh; Jan 2012-Dec 2013	Low	High	Low	Low
up_23	Philippines; Feb 2014-Jun 2016	Low	Low	Low	Low
up_25	South Africa; Jun 2012-Dec 2017	Low	Low	Low	Low
up_26	Mozambique; Jan-Dec 2011	Low	Low	Low	Low
up_28	Chile; 2012-2013	Low	Low	Low	Low
up_29	Philippines; Aug 2012-Feb 2015	Low	Low	Low	Low
up_3	Bangladesh; 2013-2014	Low	Low	Low	High
up_30	Argentina; Jun 2008-Dec 2010	Low	High	High	Low
up_31	Philippines; Jul 2000-Dec 2004	Low	High	Low	High
up_32	South Africa; 2015-2017	Low	Low	Low	High
up_33	South Africa; Jan 2000-Dec 2002	Low	Low	Low	High
up_35	Jordan; Mar 2010-Mar 2013	Low	Low	Low	Low
up_36	Kenya; Jan 2007- Dec 2017	Low	Low	Low	Low
up_38	Argentina; May 2011-Aug 2013	Low	Low	Low	Low
up_5	Germany; Jan 2010-Dec 2014	Low	Low	Low	Low
up_6	South Africa; 2010-2015	Low	Low	Low	Low
up_7	South Africa; 2010-2015	Low	Low	Low	Low
up_8	USA; 2010-2016	Low	Low	Low	High
up_9	Philippines; Sep 2012-Jul 2016	Low	Low	Low	Low

Appendix 13. Details of individual studies

Glossary of abbreviations used in this section

Abbreviation	Full name
**ALRI**	acute lower respiratory infection
**ARI**	acute respiratory infection
**BAL**	bronchoalveolar lavage
**DFA**	Direct fluorescent antibody test
**EIA**	EIA: enzyme immunoassay.
**hMPV–ALRI**	human metapneumovirus–associated ALRI
**IFA**	indirect immunofluorescence assay
**m**	month(s)
**NA**	Not applicable
**NPA**	nasopharyngeal aspirate
**NPS**	nasopharyngeal swab
**NPW**	nasopharyngeal wash
**NS**	nasal swab
**NW**	Nasal wash
**OP specimen**	oropharyngeal specimen
**OPS**	oropharyngeal swab
**PCR**	polymerase chain reaction
**TS**	throat swab

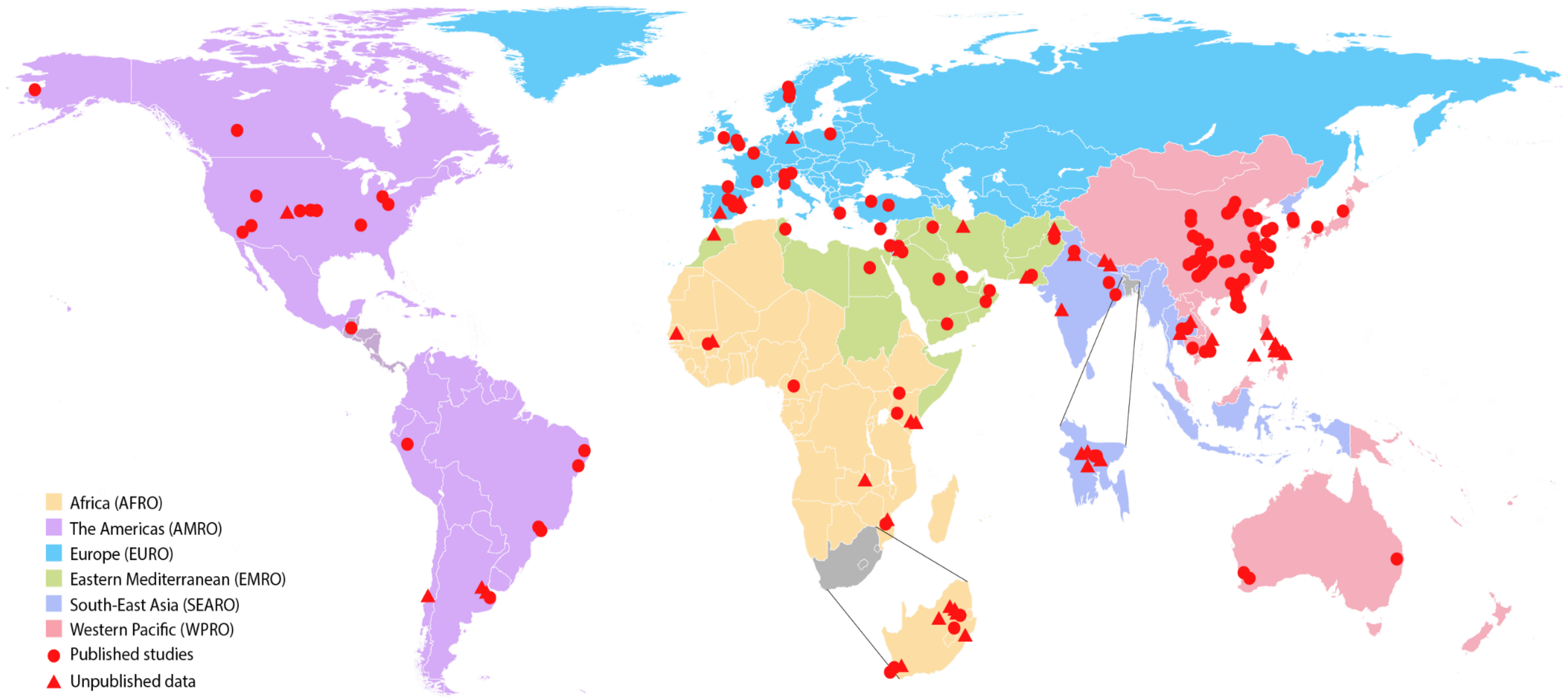

Figure S13.1. Location of included studies on incidence rate, hospital admission rate, proportion positives, and in–hospital case–fatality ratios of human metapneumovirus–associated ALRI.

Table S13.1. Description of included studies reporting incidence rates of hMPV–associated ALRI cases (per 1,000 children per year) in children under five years. *†

Location (reference)	Case Definition	Denominator type	Specimen and diagnostic test	Incidence rates				
				0–5 m	6–11 m	12-23 m	24-59 m	0–59 m
**Paarl, South Africa (Jun 2012–Dec 2017) (Zar and colleagues, unpublished)**	ALRI	Defined population estimates	NPS; PCR	60.4	37.3	23	12	24.5
**Oshikhandass, Pakistan (Dec 2012–Nov 2013) (Rasmussen and colleagues, unpublished)**	ALRI	Defined population estimates	NPS; PCR	0	0	20.5	13.6	12.3
**Kamalapur, Bangladesh (2013–2014) (Brooks and colleagues, unpublished)**	ALRI	Defined population estimates	NPW; PCR	6.9	12.9	37.5	38.2	20.7
**Bhaktapur, Nepal (2004–2007) (Strand and colleagues, unpublished)**	ALRI	Defined population estimates	NPA; PCR	28.8	27.5	23.2	··	··
**Faridabad, India (Aug 2012–Aug 2014) (Krishanan and colleagues, unpublished)**	ALRI	Census derived population	OP and nasal specimens; PCR	96	55.4	41.3	10.8	27.7
**San Marcos, Cajamarca, Peru; rural (Mar 2009 – Sep 2011) (Wu et al. 2015)[24]**	ALRI–Fever/ALRI	Defined population estimates	NS; PCR	··	··	··	··	··
**Nashville, USA (1976–2001) (Williams et al. 2004)[25]**	ALRI	Defined population estimates	NW; PCR and culture	··	··	··	··	17.5
**Brisbane, Australia (Sep 2010–Oct 2014) (Sarna et al. 2018)[26]**	ALRI	Defined population base	NS; PCR	··	··	··	··	··
**Perth, Australia (Jul 1996–Jul 1999) (Kusel et al. 2006)[27]**	ALRI	Defined population base	NPA; PCR	··	··	··	··	··
**Navajo and White Mountain Apache, USA (2009) (Bhat et al, 2013)[28]**	ALRI	Defined population base	NW; PCR	··	··	··	··	··
**Kamalapur, Bangladesh (2004-2008) (Havers, et al. 2019)[29]**	ALRI	Defined population base	NPW; PCR	176.2	116.7	85.5	12.1	31.2

* ALRI: acute lower respiratory infections according to WHO IMCI definition. ALRI–Fever: ALRI with fever. NPS: nasopharyngeal swab. PCR: polymerase chain reaction. NPW: nasopharyngeal wash. NPA: nasopharyngeal aspirate. OP specimens: oropharyngeal specimens. NS: nasal swab. NW: nasal wash.
† ··: not available. Some included studies did not provide data for any of these listed age groups, but provided data for other age groups.

Table S13.2. Description of included studies reporting incidence rates of hMPV–associated severe ALRI cases (per 1,000 children per year) in children under five years*†

Location (reference)	Case Definition	Denominator type	Specimen and diagnostic test	Incidence rates				
				0–5 m	6–11 m	12-23 m	24-59 m	0–59 m
**Karachi, Pakistan (Oct 2011–July 2014) (Ali et al. 2016)[30]**	sALRI	Defined population estimates	NPS; PCR	2.5	··	··	··	··
**Paarl, South Africa (Jun 2012–Dec 2017) (Zar and colleagues, unpublished)**	sALRI	Defined population estimates	NPS; PCR	49.5	29	12.6	9.0	18.0
**Kamalapur, Bangladesh (2014) (Brooks and colleagues, unpublished)**	sALRI	Defined population estimates	NPW; PCR	1.2	1.8	0	2.2	1.3
**Faridabad, India (Aug 2012–Aug 2014) (Krishanan and colleagues, unpublished)**	sALRI	Census derived population	OP and nasal specimens; PCR	55.6	31.1	26.4	5.7	16.8

* sALRI: severe acute lower respiratory infections according to 2005 WHO IMCI. NPS: nasopharyngeal swab. PCR: polymerase chain reaction. NPW: nasopharyngeal wash. OP specimens: oropharyngeal specimens.
† ··: not available.

Table S13.3. Description of included studies reporting hospital admission rates of hMPV–associated ALRI cases in children younger than five years*†

Location (reference)	Case Definition	Denominator type	Specimen and test	Hospital admission rates				
				0–5 m	6–11 m	12-23 m	24-59 m	0–59 m
**NVSN sites, USA (Nov–May, 2003–2009) (Edwards et al. 2013)[31]**	Fever; ARI	Census derived population	NS and TS; PCR	3	2	1.2	0.5	1
**Soweto, South Africa (Feb 2009–Dec 2012) (Groome et al. 2015)[32]**	ALRI	Census derived estimate	NPA; PCR	··	··	1.7	0.3	1.8
**Sa Kaeo and Nakhon Phanom, Thailand (2005–2010) (Hasan et al. 2014)[33]**	ARI	Census derived population	NPS and serum specimens; PCR, serologic test and culture	1.8	··	··	1.3	2
**Memphis, Nashville, and Salt Lake City (EPIC), USA (Jan 2010–June 2012) (Jain et al. 2015)[34]**	ALRI	Census derived population	NPS and OPS; PCR and serologic testing	··	··	··	0.4	0.6
**Rochester, New York; Nashville, Tennessee (NVSN), USA (Aug 2000–Sep 2001) (Mullins et al. 2004)[35]**	Fever; ARI	Census derived population	NS and TS; PCR	··	··	1.3	0.1	0.6
**Leicester, UK (Oct 2001–June 2002) (Nicholson et al. 2006)[36]**	Fever; ARI	Census derived population	NS and TS; PCR	··	··	2.4	··	··
**Salt Lake County, Utah, USA (July 2007–June 2013) (Davis et al. 2016)[37]**	ALRI	Census derived population	··; DFA and PCR	2	2.5	1.8	0.5	1.1
**Haryana, India (Aug 2009–July 2011) (Broor et al. 2014)[38]**	Fever; ARI	Census derived population	NS and TS; PCR	··	··	··	··	0.2
**Santa Rosa, Quetzaltenango, Guatemala (Nov 2007–Dec 2012) (McCracken et al. 2014)[39]**	ALRI	Census derived population	NPS and OPS; PCR	2.1	··	··	0.3	1
**Kakuma and Dadaab, Kenya (Sep 2007-Aug 2010) (Ahmed et al, 2012) [40]**	ALRI	Census derived population	NPS and OPS; PCR	NA	NA	NA	NA	3.6
**Navajo and White Mountain Apache, USA (Jan-Dec 2009) (Bhat et al, 2013) [28]**	ALRI	Defined population base	NW; PCR	NA	NA	NA	NA	NA
**Gipuzkoa, Spain; (July 2004–June 2007) (Cilla et al. 2009)[41]**	ARI; ARI–Fever	Census derived population	NPA; PCR and culture	6.8	3.5	1.8	··	··
**Nakhon Phanom, Thailand (Jan 2012–Dec 2013) (Knoll and colleagues, unpublished)**	ALRI	Census derived population	NP/OP and induced sputum; PCR	··	··	0.6	0	0.1
**Sa Kaeo, Thailand (Jan 2012–Dec 2013) (Knoll and colleagues, unpublished)**	ALRI	Census derived population	NP/OP and induced sputum; PCR	··	··	0	0.1	0.1
**Basse, Gambia (2011-2013) (Knoll and colleagues, unpublished)**	ALRI	NA	NPS, OPS, induced sputum specimens; PCR	10.9	9.8	4	0.5	3.3
**Kawayan and Caibiran, Philippines (Feb 2014–Jun 2016) (Oshitani and colleagues, unpublished)**	ALRI	Defined population estimates	NPS; PCR	12.1	2.2	2.0	1.2	2.2

* ARI: acute respiratory infections requiring hospital admission. ALRI: acute lower respiratory infections requiring hospital admission. ARI–Fever: hospitalised acute respiratory infections with fever. ALRI–Fever: hospitalised acute lower respiratory infections with fever. NS: nasal swab. TS: throat swab. PCR: polymerase chain reaction. NPA: nasopharyngeal aspirate. NPS: nasopharyngeal swab. OPS: oropharyngeal swab. NPW: nasopharyngeal wash. IFA: indirect immunofluorescence assay. DFA: direct immunofluorescence assay.

† ··: not available. Some included studies did not provide data for any of these listed age groups but provided data for other age groups.

Location (reference)	Case Definition	Denominator type	Specimen and test	Hospital admission rates				
				0–5 m	6–11 m	12-23 m	24-59 m	0–59 m
**Nha Trang city, Viet Nam (Jan 2007–Dec 2014) (Yoshida and colleagues, unpublished)**	ALRI	Census derived population	NPS; PCR	··	··	0.8	0.2	0.6
**Paarl, South Africa (Jun 2012–Dec 2017) (Zar and colleagues, unpublished)**	ALRI	Defined population estimates	NPS; PCR	18	10.9	3.1	1.3	5.5
**Manhiça, Mozambique (Jan–Dec 2011) (Bassat and colleagues, unpublished)**	ALRI	Census derived population	NPA; PCR	1.1	2.1	0.4	0.3	0.6
**Concepcion, Chile (2012–2013) (Fasce and colleagues, unpublished)**	ALRI	Census derived population	NPA; IFA	··	··	0.3	0.1	0.3
**Kamalapur, Bangladesh (2013–2014) (Brooks and colleagues, unpublished)**	ALRI	Defined population estimates	NPW; PCR	1.5	1.9	1.6	2.8	1.8
**Ciudad de Buenos Aires, Argentina (Jun 2008–Dec 2010) (Echavarria and colleagues, unpublished)**	ARI	Defined population estimates	NPA; DFA	0	··	··	0.5	0.6
**Multiple sites, Philippines (Jul 2000–Dec 2004) (Lucero and colleagues, unpublished)**	ALRI	Defined population estimates	NPA and NPS; Viral culture	4.4	6.4	2.6	··	··
**Soweto, Gauteng, South Africa (2015–2017) (Nunes and colleagues, unpublished)**	ALRI	Census derived population	NPS; PCR	··	··	1	0.4	0.9
**Soweto, Gauteng, South Africa (Jan 2000–Dec 2002) (Madhi and colleagues, unpublished)**	ALRI	Defined population estimates	NPA; PCR	9.6	7.4	2.6	1.1	2.6
**Pune, India (May 2009–Apr 2013) (Hirve and colleagues, unpublished)**	ALRI	Census derived population	NPS; PCR	1.1	0.9	1	0.3	0.5
**Amman, Jordan (Mar 2010–Mar 2013) (Khuri-Bulos and colleagues, unpublished)**	ALRI	Census derived population	NS and TS; PCR	1.5	1	0.4	··	··
**Kilifi, Kenya (Jan 2007– Dec 2017) (Nokes and colleagues, unpublished)**	ALRI	Census derived population	NPS; PCR	3.3	3.4	0.9	0.3	1.1
**Valencia Region, Spain (2014–2017) (Mira Iglesias and colleagues, unpublished)**	All	Census derived population	NPS and NS; PCR	··	··	0.3	0.1	0.4
**Buenos Aires, Argentina (May 2011–Aug 2013) (Polack and colleagues, unpublished)**	ALRI	Census derived population	NPA; PCR	3.8	3.5	0.7	··	··
**Klerksdorp, South Africa (2010–2015) (Cohen and colleagues, unpublished)**	ALRI	Census derived population	NPA; PCR	1.8	1	0.6	0.1	0.5
**Pietermaritzburg, Kwa–Zulu Natal Province, South Africa (2010–2015) (Cohen and colleagues, unpublished)**	ALRI	Census derived population	NPA; PCR	2.2	1.6	0.5	0.2	0.6
**NVSN sites, USA (Oct 2001– Sep 2003) (Williams et al. 2004)[42]**	ARI–Fever; Fever	Census derived population	NS and TS; PCR and culture	··	··	··	··	1.2
**San Marcos, Cajamarca, Peru; rural (Mar 2009 –Sep 2011) (Wu et al. 2015)[24]**	ALRI–Fever/ALRI	Defined population estimates	NS; PCR	··	··	··	··	··
**Madrid, Spain (2011-2012) (Olabarrieta et al, 2015)[43]**	ALRI	Defined population base	NPA; PCR	··	··	··	··	··
**Sør–Trøndelag County, Norway (Nov 2006–July 2015) (Moe et al. 2017) [44]**	ALRI	Census derived estimates	NPA; PCR	··	··	··	··	1.8
**Manhica, Mozambique (Sep 2006–Sep 2007) (O'Callaghan-Gordo et al. 2011)[45]**	ALRI	Census derived estimate	NPA; PCR	··	··	··	··	1.8
**Asembo, Kenya (Mar 2007–Feb 2008) (Feikin et al. 2013)[46]**	ALRI	Census derived estimate	NPS or OPS; PCR	··	··	··	··	14.6

**Location (reference)**	**Case Definition**	**Denominator type**	**Specimen and test**	**Hospital admission rates**				
				**0–5 m**	**6–11 m**	**12-23 m**	**24-59 m**	**0–59 m**
**Bangladesh (2010–2014) (Homaira and colleagues, unpublished)**	ARI–Fever; ALRI	Census derived population	NS and TS; PCR	··	··	··	··	1.1

Table S13.4. Description of included studies reporting hCFRs (%) of hMPV–associated ALRI cases in children under five years*

Location (reference)	Case definition	Specimen and test	0–5 m		6–11 m		12–59 m		0–59 m	
			Cases (No.)	hCFR (%)	Cases (No.)	hCFR (%)	Cases (No.)	hCFR (%)	Cases (No.)	hCFR (%)
**Seoul, Korea (Dec 2003–Feb 2005) (Chung et al. 2006)[47]**	ARI	··; PCR	3	0	7	0	18	5.6	25	4.0
**Gipuzkoa, Spain; (July 2004–June 2007) (Cilla et al. 2009)[41]**	ARI; ARI–Fever	NPA; PCR and culture	··	··	··	··	··	··	··	··
**Trondheim, Norway (Nov 2002–April 2003) (Dollner et al. 2004)[48]**	ARI	NPA; PCR	··	··	··	··	··	··	··	··
**Madrid, Spain (Oct 2000–June 2003) (García-García et al. 2006)[49]**	ARI	NPA; PCR	··	··	··	··	··	··	··	··
**Iowa, USA (Oct 2001–May 2004) (Gray et al. 2006)[50]**	ARI	mainly NW; PCR	2	0	3	0	14	7.1	15	6.7
**Columbus, Ohio, USA (June 2007–June 2010) (Hahn et al. 2013)[51]**	ALRI	··; DFA	··	··	··	··	··	··	··	··
**Yukon Kuskokwim Delta, USA (Oct 2005–Sep 2007) (Singleton et al. 2010) [52]**	ALRI	NP specimens; PCR	··	··	··	··	··	··	··	··
**NVSN sites, USA (Oct 2001– Sep 2003)(Williams et al. 2010)[53]**	ARI–Fever; Fever	NS and TS; PCR and culture	··	··	··	··	··	··	42	0
**Salt Lake County, Utah, USA (July 2007–June 2013) (Davis et al. 2016)[37]**	ALRI	··; DFA and PCR	129	0.8	··	··	··	··	725	0.3
**Hiroshima, Japan (Takao et al. 2003)[54]**	ALRI	NPA; PCR	··	··	··	··	··	··	7	0
**Sør–Trøndelag County, Norway (Nov 2006–July 2015) (Moe et al. 2017)[44]**	ALRI	NPA; PCR	29	0	34	0	97	1	160	0.6
**Valencia Region, Spain (2014–2017) (Mira Iglesias and colleagues, unpublished)**	All	NPS and NS; PCR	29	0	10	0	17	0	56	0
**Berlin, Germany (Jan 2010–Dec 2014) (Rath and colleagues, unpublished)**	ALRI	NPS; PCR	27	3.7	27	0	78	0	132	0.8
**Aurora, Colorado, United States of America (2010–2016) (Simões and colleagues, unpublished)**	ALRI	NW; PCR (and DFA)	106	0	93	1.1	432	0	631	0.2
**Islamabad, Pakistan (March 2011–April 2012) (Bashir et al. 2017)[55]**	ALRI	NPS or OPS; PCR	··	··	··	··	··	··	··	··
**Haryana, India (Aug 2009–July 2011) (Broor et al. 2014a)[56]**	Fever; ARI	NS and TS; PCR	··	··	··	··	··	··	3	0
**São Paulo city, Brazil (March 2008–Feb 2010) (Durigon et al. 2015)[57]**	ARI	NPA; PCR	··	··	··	··	··	··	··	··
**Takeo Province and Kampong Cham Province, Cambodia (April 2007–Feb 2010) (Guerrier et al. 2013)[58]**	ALRI	NPA; PCR	··	··	··	··	··	··	43	0

* ARI: acute respiratory infections requiring hospital admission. ALRI: acute lower respiratory infections requiring hospital admission. ALRI–Fever: hospitalised acute lower respiratory infections with fever. NS: nasal swab. TS: throat swab. PCR: polymerase chain reaction. NPA: nasopharyngeal aspirate. NPS: nasopharyngeal swab. NW: nasopharyngeal wash. OPS: oropharyngeal swab. NPW: nasopharyngeal wash. BAL: bronchoalveolar lavage. IFA: indirect immunofluorescence assay. DFA: direct immunofluorescence assay. ··: not available.

Location (reference)	Case definition	Specimen and test	0–5 m		6–11 m		12–59 m		0–59 m	
			Cases (No.)	hCFR (%)	Cases (No.)	hCFR (%)	Cases (No.)	hCFR (%)	Cases (No.)	hCFR (%)
**Parow, South Africa (June–August 2002) (Ijpma et al. 2004)[59]**	ARI	NPA; PCR	··	··	··	··	··	··	··	··
**Khon Kaen, Thailand (April 2002–Aug 2004) (Teeratakulpisarn et al. 2007)[60]**	ALRI	NP secretion; PCR	··	··	··	··	··	··	··	··
**KFSHRC, Riyadh, Saudi Arabia (July 2007–Nov 2008) (Al Hajjar et al. 2011)[61]**	ARI	NPA and BAL; PCR	1	0	1	0	7	28.6	9	22.2
**Ankara, Turkey (Nov 2011–May 2012) (Azkur et al. 2014)[62]**	ALRI	NPS; PCR	··	··	··	··	··	··	··	··
**Bamako, Mali (July 2011–Dec 2012) (Benet et al. 2015)[63]**	ALRI	··; PCR	··	··	··	··	··	··	12	0
**Soweto, Gauteng, South Africa (Jan 2010–Dec 2013) (Cohen et al. 2016)[64]**	ALRI	NPA; PCR	147	0.7	··	··	··	··	··	··
**Ho Chi Minh City, Vietnam (Nov 2004–Jan 2008) (Do et al. 2011a)[65]**	ARI	NS, TS, and NPA; PCR	··	··	··	··	··	··	20	0
**Istanbul, Turkey (Oct 2006–March 2007) (Hatipoglu et al. 2011)[66]**	ALRI–Fever; ALRI	NPS; PCR	··	··	··	··	··	··	7	0
**Amman, Jordan (Dec 2003–May 2004)(Kaplan et al. 2006)[67]**	ARI	NPA; PCR	··	··	··	··	··	··	8	0
**Santa Maria (May 2005–May 2007) (Lozano C et al. 2009)[68]**	ALRI	NPA; PCR	··	··	··	··	··	··	24	0
**Santa Rosa, Quetzaltenango, Guatemala (Nov 2007–Dec 2012) (McCracken et al. 2014)[39]**	ALRI	NPS and OPS; PCR	··	··	··	··	··	··	508	1.8
**Buenos Aires, Argentina (Nov–Dec 2009) (Pérez et al. 2012)[†69]**	ARI	NPA; IFA	··	··	··	··	··	··	··	··
**Suzhou, China (Jan 2006–Dec 2007) (Wang et al. 2009)[70]**	ARI	respiratory specimens; PCR	··	··	··	··	··	··	··	··
**Sousse area, Tunisia (Sep 2013–Dec 2014) (Brini et al. 2017)[71]**	ARI	NPA; PCR	··	··	··	··	··	··	60	8.3
**Egypt, Jordan, Oman, Qatar and Yemen (Dec 2007–Feb 2014) (Horton et al. 2017)[72]**	ARI	NPS and OPS; PCR	··	··	··	··	··	··	425	2.1
**Manhica, Mozambique (Sep 2006–Sep 2007) (O'Callaghan-Gordo et al. 2011)[45]**	ALRI	NPA; PCR	··	··	··	··	··	··	29	10.3
**Recife, Brazil (Apr 2008–Mar 2009) (Bezerra et al. 2011) [73]**	ARI	NPA; PCR	··	··	··	··	··	··	··	··
**Cuangdong, China (June 2006–June 2007) (林创兴 et al. 2009)[74]**	ALRI	NP secretions, TS; PCR	··	··	··	··	··	··	··	··
**Hunan Provincial People's Hospital, Changsha, China (Sep 2007–Aug 2008) (梁沫 et al. 2012)[75]**	ALRI	NPA; PCR	··	··	··	··	··	··	··	··
**Suzhou, China (Jan 2009–Dec 2012) (邱秀娟 2015)[76]**	ARI	NPA; PCR	··	··	··	··	··	··	472	0
**Suzhou, China (Jan 2006–Dec 2008) (骆亚丽 2009)[77]**	ARI	NPA; PCR	··	··	··	··	··	··	439	0

[†] Among 13 hMPV-associated ALRI in infants <1 year, zero deaths were reported.

Location (reference)	Case definition	Specimen and test	0–5 m		6–11 m		12–59 m		0–59 m	
			Cases (No.)	hCFR (%)	Cases (No.)	hCFR (%)	Cases (No.)	hCFR (%)	Cases (No.)	hCFR (%)
**Shanghai, China (Dec 2006–Feb 2008) (沈军 2009)[78]**	ALRI	NPS; PCR	3	0	5	0	15	0	··	··
**Rabat, Morocco (Nov 2010–Dec 2011) (Bassat and colleagues, unpublished)**	ALRI	NPA; PCR	15	20	17	0	37	0	69	4.3
**Taclobal, Philippines (May 2008–Feb 2015) (Oshitani and colleagues, unpublished)**	ALRI	NPS; PCR	27	7.4	24	4.2	58	0	109	2.8
**Muntinlupa, Philippines (Sep 2012–Feb 2015) (Oshitani and colleagues, unpublished)**	ALRI	NPS; PCR	1	0	··	··	2	0	3	0
**, Bangladesh (2010–2014) (Homaira and colleagues, unpublished)**	ARI–Fever; ALRI	NS and TS; PCR	30	6.7	8	0	7	0	45	4.4
**Nakhon Phanom, Thailand (Jan 2012–Dec 2013) (Deloria-Knoll and colleagues, unpublished)**	ALRI	NP/OP and induced sputum; PCR	··	··	··	··	··	··	2	0
**Sa Kaeo, Thailand (Jan 2012–Dec 2013) (Deloria-Knoll and colleagues, unpublished)**	ALRI	NP/OP and induced sputum; PCR	1	0	··	··	1	0	2	0
**Basse, Gambia (Nov 2011–Nov 2013) (Deloria-Knoll and colleagues, unpublished)**	ALRI	NPS, OPS, induced sputum specimens; PCR	72	0	73	1.4	74	1.4	219	0.9
**Lusaka, Zambia (Oct 2011 – Oct 2014) (Deloria-Knoll and colleagues, unpublished)**	ALRI	NPS, OPS, induced sputum specimens; PCR	22	9.1	19	10.5	12	0	53	7.5
**Bamako, Mali (Jan 2012 – Jan 2014) (Deloria-Knoll and colleagues, unpublished)**	ALRI	NPS, OPS, induced sputum specimens; PCR	16	0	14	0	18	5.6	48	2.1
**Kilifi, Kenya (Aug 2011–Jul 2013) (Deloria-Knoll and colleagues, unpublished)**	ALRI	NPS, OPS, induced sputum specimens; PCR	18	5.6	16	0	22	4.5	56	3.6
**Karachi, Pakistan (Aug 2009–Jul 2012) (Ali and colleagues, unpublished)**	ARI	TS; PCR	32	3.1	24	0	28	0	84	1.2
**Soweto, South Africa (Aug 2011–Aug 2013) (Deloria-Knoll and colleagues, unpublished)**	ALRI	NPS, OPS, induced sputum specimens; PCR	22	0	19	0	17	11.8	58	3.4
**Matlab, Bangladesh (Jan 2012 – Dec 2013) (Deloria-Knoll and colleagues, unpublished)**	ALRI	NPS, OPS, induced sputum specimens; PCR	6	0	6	0	14	0	26	0
**Dhaka, Bangladesh (Jan 2012–Dec 2013) (Deloria-Knoll and colleagues, unpublished)**	ALRI	NPS, OPS, induced sputum specimens; PCR	5	0	5	0	4	0	14	0
**Kawayan and Caibiran, Philippines (Feb 2014–Jun 2016) (Oshitani and colleagues, unpublished)**	ALRI	NPS; PCR	1	0	··	··	3	0	4	0
**Paarl, South Africa (Jun 2012–Dec 2017) (Zar and colleagues, unpublished)**	ALRI	NPS; PCR	9	0	6	0	5	0	20	0
**Manhiça, Mozambique (Jan–Dec 2011) (Bassat and colleagues, unpublished)**	ALRI	NPA; PCR	5	20	10	0	13	0	28	3.6
**Concepcion, Chile (2012–2013) (Fasce and colleagues, unpublished)**	ALRI	NPA; IFA	6	0	6	0	11	0	23	0
**Pueto Princesa City, Philippines (Aug 2012–Feb 2015) (Oshitani and colleagues, unpublished)**	ALRI	NPS; PCR	16	6.2	15	0	25	0	56	1.8
**Kamalapur, Bangladesh (2013–2014) (Brooks and colleagues, unpublished)**	ALRI	NPW; PCR	2	0	1	0	3	0	6	0
**Ciudad de Buenos Aires, Argentina (Jun 2008–Dec 2010) (Echavarria and colleagues, unpublished)**	ARI	NPA; DFA	··	··	2	0	3	0	5	0

Location (reference)	Case definition	Specimen and test	0–5 m		6–11 m		12–59 m		0–59 m	
			Cases (No.)	hCFR (%)	Cases (No.)	hCFR (%)	Cases (No.)	hCFR (%)	Cases (No.)	hCFR (%)
**Multiple sites, Philippines (Jul 2000–Dec 2004) (Lucero and colleagues, unpublished)**	ALRI	NPA and NPS; Viral culture	13	7.7	29	0	··	··	··	··
**Soweto, Gauteng Prov, South Africa (2015–2017) (Nunes and colleagues, unpublished)**	ALRI	NPS; PCR	63	0	58	0	30	0	151	0
**Soweto, Gauteng, South Africa (Jan 2000–Dec 2002) (Madhi and colleagues, unpublished)**	ALRI	NPA; PCR	22	4.5	34	0	70	1.4	126	1.6
**Amman, Jordan (Mar 2010–Mar 2013) (Khuri-Bulos and colleagues, unpublished)**	ALRI	NS and TS; PCR	101	1	68	0	··	··	··	··
**Kilifi, Kenya (Jan 2007– Dec 2017) (Nokes and colleagues, unpublished)**	ALRI	NPS; PCR	69	1.4	54	0	53	0	176	0.6
**Buenos Aires, Argentina (May 2011–Aug 2013) (Polack and colleagues, unpublished)**	ALRI	NPA; PCR	163	0.6	149	0.7	··	··	··	··
**Klerksdorp, South Africa (2010–2015) (Cohen and colleagues, unpublished)**	ALRI	NPA; PCR	19	5.3	11	0	18	0	50	2
**Pietermaritzburg, Kwa–Zulu Natal Province, South Africa (2010–2015) (Cohen and colleagues, unpublished)**	ALRI	NPA; PCR	41	2.4	29	0	41	0	111	0.9
**Naval, Philippines (Sep 2012–Jul 2016) (Oshitani and colleagues, unpublished)**	ALRI	NPS; PCR	12	0	8	0	21	0	41	0

Table S13.5. Description of included studies reporting proportions of hospitalised hMPV–associated ALRI cases in children under five years[*†‡]

Location (reference)	Case definition	Specimen and test	0–5 m		6–11 m		0–59 m	
			Tested ALRI (No.)	Proportion (%)	Tested ALRI (No.)	Proportion (%)	Tested ALRI (No.)	Proportion (%)
**Montpellier, France (Nov 2003–Oct 2004) (Foulongne et al. 2006)[79]**	ARI	NPA; PCR	296	8.8	169	8.9	589	9.0
**Liverpool, UK (Oct 2004–Oct 2005) (Hopkins et al. 2008)[80]**	ARI	NPA and non–bronchoscopic BAL; PCR	··	··	··	··	··	··
**Reims, France (Oct 2007–Sep 2008) (Huguenin et al. 2012)[81]**	ALRI	NPA; PCR	··	··	··	··	··	··
**Pisa, Italy (Jan 2000–May 2002) (Maggi et al. 2003)[82]**	ARI	NS; PCR	··	··	··	··	··	··
**NVSN sites, USA (Oct 2001– Sep 2003) [53]**	ARI–Fever; Fever	NS and TS; PCR and culture	··	··	··	··	1104	3.8
**Beersheba, Israel (Nov 2001–Oct 2005) (Wolf et al. 2010)[83]**	ALRI	NPW; PCR	··	··	··	··	997	8.0
**Greece (Oct 1999–Sep 2000) (Xepapadaki et al. 2004)[84]**	ALRI	NPW; PCR	··	··	··	··	··	··
**Milan, Italy (2004–2008) (Zappa et al. 2011)[85]**	ALRI	Pharyngeal swabs; PCR	144	9.0	36	8.3	··	··
**Seoul, Korea (Sep 2011–Aug 2012) (Eem et al. 2014)[86]**	ARI	NPS; PCR	··	··	··	··	··	··
**Tokyo, Japan (April 2007–March 2012) (Hamada et al. 2014)[87]**	ALRI	NS; PCR	··	··	··	··	··	··
**Perth, Australia (Jan 2000–Dec 2005) (Moore et al. 2012)[88]**	ALRI	NPA; PCR	··	··	··	··	1179	13.7
**Leganes, Madrid, Spain (Sep 2005–Aug 2008) (Calvo et al. 2010)[89]**	ALRI	NPA; PCR	··	··	··	··	··	··
**Warsaw, Poland (Oct 2008–April 2011) (Pancer et al. 2014)[90]**	ARI	NPS; PCR and EIA	··	··	··	··	297	10.8
**Cordoba, Spain (Jan–Dec 2011) (Rodriguez et al. 2016)[91]**	ARI	NPA; DFA	··	··	··	··	223	4.0
**Melegnano, Italy (Oct 2004–Sep 2006) (Canducci et al. 2008)[92]**	ARI	NPA; PCR	··	··	··	··	··	··
**Spain (Jan 2011–Jan 2013) (Cebey-López et al. 2015)[93]**	ALRI	NP sample; PCR	··	··	··	··	··	··

[*] ARI: acute respiratory infections requiring hospital admission. ALRI: physician diagnosed acute lower respiratory infections requiring hospital admission. ARI–Fever: hospitalised acute respiratory infections with fever. NS: nasal swab. TS: throat swab. PCR: polymerase chain reaction. NPA: nasopharyngeal aspirate. NPS: nasopharyngeal swab. NPW: nasopharyngeal wash. NW: nasopharyngeal wash. OPS: oropharyngeal swab. BAL: bronchoalveolar lavage. IFA: indirect immunofluorescence assay. DFA: direct immunofluorescence assay. EIA: enzyme immunoassay.
[†] ··: Not available. Some included studies did not provide data for 0–5 m, 6–11 m, or 0–59 m while provided data for other age groups (e.g., 0–35 months).
[‡] Proportion of hMPV in total ALRI cases that were tested.

Location (reference)	Case definition	Specimen and test	0–5 m		6–11 m		0–59 m	
			Tested ALRI (No.)	Proportion (%)	Tested ALRI (No.)	Proportion (%)	Tested ALRI (No.)	Proportion (%)
**London, UK (2009–2012) (Cebey-López et al. 2015)[93]**	NA	NP sample; PCR	··	··	··	··	··	··
**Nicosia, Cyprus (Nov 2010–Oct 2013) (Richter et al. 2016)[94]**	ARI	NS; PCR	··	··	··	··	··	··
**Gipuzkoa, Spain; (July 2004–June 2007) (Cilla et al. 2009)[41]**	ARI; ARI–Fever	NPA; PCR and culture	386	10.1	153	13.1	··	··
**Valencia Region, Spain (2014–2017) (Mira Iglesias and colleagues, unpublished)**	All	NPS and NS; PCR	920	3.2	245	4.1	1929	2.9
**Berlin, Germany (Jan 2010–Dec 2014) (Rath and colleagues, unpublished)**	ALRI	NPS; PCR	731	4.8	424	9.2	2516	7.2
**Aurora, Colorado, United States of America (2010–2016) (Simões and colleagues, unpublished)**	ALRI	NW; PCR (and DFA)	2173	4.9	862	10.8	6424	9.8
**São Paulo city, Brazil (March 2008–Feb 2010) (Durigon et al. 2015)[57]**	ARI	NPA; PCR	··	··	··	··	··	··
**Aracaju´, Salvador, Recife, and Maceio, Brazil (April 2012–March 2013) (Gurgel et al. 2016)[95]**	ALRI	NPA; PCR	··	··	··	··	··	··
**Sa Kaeo and Nakhon Phanom, Thailand (2005–2010) (Hasan et al. 2014)[33]**	ARI	NPS and serum specimens; PCR, serologic test and culture	397	2	··	··	3810	2.9
**Lucknow, India (May 2011–April 2013) (Jain et al. 2014)[96]**	ALRI	NPA; PCR	··	··	··	··	235	5.1
**Yaounde, Cameroon (Sep 2011–Sep 2013) (Kenmoe et al. 2016)[97]**	ARI–Fever	NPS; PCR	··	··	··	··	307	3.6
**Sultan Qaboos University Hospital, Oman; (Dec 2007–Dec 2008) (Khamis et al. 2012) [98]**	ARI	NPA; PCR	··	··	··	··	518	1.2
**Shandong, China (Jan 2011–Dec 2013) (Liu et al, 2015)[99]**	ARI–Fever	TS; PCR	··	··	··	··	243	1.2
**Beijing, China (July 2008–June 2010) (Lu et al. 2013)[100]**	ALRI	NPA; PCR	428	7	155	9.7	··	··
**Sao Paulo, Brazil (2003–2006) (Oliveira et al. 2009)[101]**	ARI	NPA and NS; PCR	··	··	··	··	1670	11.4
**Yukon Kuskokwim Delta, USA (Oct 2005–Sep 2007) (Singleton et al. 2010)[52]**	ALRI	NP specimens; PCR	··	··	··	··	··	··
**Arizona, Mexico (Oct 2010–Sep 2014) (Wansaula et al. 2016)[102]**	ARI–Fever; ALRI	NPS; PCR	··	··	··	··	17	11.8
**Lanzou, China (Dec 2011–Nov 2012) (Yan et al. 2017)[103]**	ALRI	NPA; PCR	··	··	··	··	360	12.8
**Ho Chi Minh City, Vietnam (May 2009–Dec 2010) (Do et al. 2016)[104]**	ARI	NPS; PCR	··	··	··	··	··	··

Location (reference)	Case definition	Specimen and test	0–5 m		6–11 m		0–59 m	
			Tested ALRI (No.)	Proportion (%)	Tested ALRI (No.)	Proportion (%)	Tested ALRI (No.)	Proportion (%)
**Lanzhou, China (Jan–Dec 2011) (Huang et al. 2013)[105]**	ARI	TS; PCR	··	··	··	··	··	··
**Haryana, India (Aug 2009–July 2011) (Broor et al. 2014a)[56]**	All	NS and TS; PCR	··	··	··	··	245	1.2
**Guangzhou, China (July 2009–June 2014) (Liao et al. 2015)[106]**	ARI	Pharyngeal swabs; PCR	··	··	··	··	··	··
**Beijing, China (March 2010–Feb 2012) (Liu et al. 2013)[107]**	ARI	TS; PCR	··	··	··	··	··	··
**Kolkata, India (April 2010–March 2011) (Mazumdar et al. 2013)[108]**	ALRI	NS and TS; PCR	··	··	··	··	108	0.9
**Shantou, China (Jan–Dec 2007) (Ou et al. 2009)[109]**	ALRI	NPA; PCR	··	··	··	··	345	3.2
**Hangzhou, China (Jan–Dec 2011) (Wang et al. 2013)[110]**	ALRI	NPA; DFA	··	··	··	··	··	··
**Changsha, China (Sep 2007–Aug 2008) (Xiao et al. 2012)[111]**	ALRI	NPA; PCR	350	6.6	320	7.8	1123	6.3
**Beijing, China (Feb 2011–Jan 2012) (Zhang et al. 2015)[112]**	ALRI	Tracheal aspirate; PCR	··	··	··	··	··	··
**Dhaka, Bangladesh (Aug 2014–Jul 2015) (Bhuyan et al. 2017)[113]**	ARI	NS; PCR	··	··	··	··	200	13
**Sulaimani, Iraq (Apr 2011–Mar 2012) (TAG 2015)[114]**	ARI	NPS and TS; ··	··	··	··	··	300	16
**Manhica, Mozambique (Sep 2006–Sep 2007) (O'Callaghan-Gordo et al. 2011)[45]**	ALRI	NPA; PCR	··	··	··	··	807	4.8
**Asembo, Kenya (Mar 2007–Feb 2008) (Feikin et al. 2013)[46]**	ALRI	NPS or OPS; PCR	··	··	··	··	350	4.9
**Chongqing, China (April 2006–March 2008) (Chen et al. 2010)[115]**	ALRI	NPA; PCR	428	27.8	··	··	··	··
**Recife, Brazil (Apr 2008–Mar 2009) (Bezerra et al, 2011)[73]**	ARI	NPA; PCR	··	··	··	··	211	9.5
**Cape Town, South Africa (2003–2004) (Smuts 2008)[116]**	ARI	NPA, tracheal aspirate, BAL; PCR	··	··	··	··	1055	2.7
**Guangdong, China (Mar 2010–Feb 2011) (Xu et al. 2012)[117]**	ARI	TS; PCR	··	··	··	··	··	··
**Changsha, China (Apr 2012–Mar 2013) (彭颖 2014)[118]**	ALRI	NPA; PCR	143	13.3	159	20.8	595	17.1
**Chongqing, China (Apr 2008–Mar 2009) (杜丽娜 2010)[119]**	ARI	NPA; PCR	··	··	··	··	··	··
**Yanting, China (Jan 2011–Dec 2012) (何杨 2015)[120]**	ARI	NPA; PCR	··	··	··	··	··	··

Location (reference)	Case definition	Specimen and test	0–5 m		6–11 m		0–59 m	
			Tested ALRI (No.)	Proportion (%)	Tested ALRI (No.)	Proportion (%)	Tested ALRI (No.)	Proportion (%)
**Jiaxing, China (Jan–Dec 2010) (盛曙君 2013)[121]**	ARI	NPA; DFA	2153	2.1	690	1.6	··	··
**Beijing, China (Oct 2010–Sep 2012) (魏美晨 2013)[122]**	ARI	NP specimens; PCR	28	3.6	25	16	··	··
**Chenzhou, China (Jul 2013–Jun 2014) (吴琼 et al. 2017)[123]**	ARI–Fever	NS; PCR	··	··	··	··	489	5.3
**Wenzhou, China (Jan–Dec 2014) (张海邻 et al. 2017)[124]**	ALRI	NPA; DFA	··	··	··	··	922	3.4
**Guangzhou, China (Jan–Dec 2015) (蔡勇 et al. 2017)[125]**	ARI	NPS; PCR	216	0.9	310	5.8	··	··
**Yinchuan, China (Oct 2011–Sep 2012)(张俊华 et al. 2013)[126]**	ARI	NPS; DFA	103	12.6	152	9.9	··	··
**Baiyin, China (Jul 2012–Jul 2013) (于德山 et al. 2017) [127]**	ALRI	NPA; PCR	20	15.0	93	9.7	391	13.0
**Changsha, China (Mar 2010–Feb 2011) (赵辛 2012)[128]**	ALRI	NPA; PCR	171	4.1	173	6.9	707	4.1
**Changsha, China (Apr 2013–Mar 2014) (刘沁 et al. 2015)[129]**	ALRI	NPA; PCR	138	5.8	142	6.3	442	6.8
**Chongqing, China (Jun 2009–May 2012) (卢庆彬 2013)[130]**	ARI	NPA; PCR	1028	2.6	505	5.1	2272	3.4
**Hangzhou, China (Jan 2011–Dec 2013) (Kou et al. 2016)[131]**	ALRI	NPA; PCR	··	··	··	··	··	··
**Suzhou, China (July 2007–June 2008) (季伟 et al. 2010)[132]**	ARI	NPA; PCR	563	9.9	507	10.3	··	··
**Wenzhou, China (2014-2016) (Wen et al, 2019) [133]**	ALRI	NP secretion; PCR	··	··	··	··	··	··
**Shanghai, China (2008-2014) (Zhao et al, 2019)[134]**	ALRI	NPA; PCR	139	1.4	68	0	··	··
**Suzhou, China (2006-2015) (任吟莹, et al., 2019)[135]**	ARI	NPA; DFA	··	··	··	··	15583	1.8
**Dongguan, China (2017-2018) (孙志豪, et al., 2019)[136]**	ALRI	TS; PCR	1051	2.0	554	3.1	··	··
**Rabat, Morocco (Nov 2010–Dec 2011) (Bassat and colleagues, unpublished)**	ALRI	NPA; PCR	100	15	112	15.2	631	10.9
**Taclobal, Philippines (May 2008–Feb 2015) (Oshitani and colleagues, unpublished)**	ALRI	NPS; PCR	816	3.1	510	4.5	2420	4.2

Location (reference)	Case definition	Specimen and test	0–5 m		6–11 m		0–59 m	
			Tested ALRI (No.)	Proportion (%)	Tested ALRI (No.)	Proportion (%)	Tested ALRI (No.)	Proportion (%)
**Muntinlupa, Philippines (Sep 2012–Feb 2015) (Oshitani and colleagues, unpublished)**	ALRI	NPS; PCR	70	1.4	59	0	188	1.1
**Bangladesh (2010–2014) (Homaira and colleagues, unpublished)**	ARI–Fever; ALRI	NS and TS; PCR	451	6.7	198	4	831	5.4
**Nakhon Phanom, Thailand (Jan 2012–Dec 2013) (Deloria-Knoll and colleagues, unpublished)**	ALRI	NP/OP and induced sputum; PCR	5	0	6	0	44	2.3
**Sa Kaeo, Thailand (Jan 2012–Dec 2013) (Deloria-Knoll and colleagues, unpublished)**	ALRI	NP/OP and induced sputum; PCR	7	14.3	11	0	51	3.9
**Tehran, Iran (Islamic Republic of) (Mar 2010– Mar 2013) (Vahid and colleagues, unpublished)**	ALRI	NPS and TS; PCR	78	1.3	26	7.7	158	5.7
**Lusaka, Zambia (Oct 2011 – Oct 2014) (Deloria-Knoll and colleagues, unpublished)**	ALRI	NPS, OPS, induced sputum specimens; PCR	314	7	143	13.3	590	9
**Bamako, Mali (Jan 2012 – Jan 2014) (Deloria-Knoll and colleagues, unpublished)**	ALRI	NPS, OPS, induced sputum specimens; PCR	297	5.4	151	9.3	659	7.3
**Kilifi, Kenya (Aug 2011–Jul 2013) (Deloria-Knoll and colleagues, unpublished)**	ALRI	NPS, OPS, induced sputum specimens; PCR	185	9.7	116	13.8	566	9.9
**Karachi, Pakistan (Aug 2009–Jul 2012) (Ali and colleagues, unpublished)**	ARI	TS; PCR	372	8.6	295	8.1	1150	7.3
**Soweto, South Africa (Aug 2011–Aug 2013) (Deloria-Knoll and colleagues, unpublished)**	ALRI	NPS, OPS, induced sputum specimens; PCR	431	5.1	212	9	866	6.7
**Matlab, Bangladesh (Jan 2012 – Dec 2013) (Deloria-Knoll and colleagues, unpublished)**	ALRI	NPS, OPS, induced sputum specimens; PCR	94	6.4	74	8.1	327	8
**Dhaka, Bangladesh (Jan 2012–Dec 2013) (Deloria-Knoll and colleagues, unpublished)**	ALRI	NPS, OPS, induced sputum specimens; PCR	42	11.9	47	10.6	198	7.1
**Kawayan and Caibiran, Philippines (Feb 2014–Jun 2016) (Oshitani and colleagues, unpublished)**	ALRI	NPS; PCR	39	2.6	28	0	123	3.3
**Nha Trang city, Viet Nam (Jan 2007–Dec 2014) (Yoshida and colleagues, unpublished)**	ALRI	NPS; PCR	255	2.4	241	3.3	1300	4.9
**Paarl, South Africa (Jun 2012–Dec 2017) (Zar and colleagues, unpublished)**	ALRI	NPS; PCR	102	8.8	42	14.3	201	10

Location (reference)	Case definition	Specimen and test	0–5 m		6–11 m		0–59 m	
			Tested ALRI (No.)	Proportion (%)	Tested ALRI (No.)	Proportion (%)	Tested ALRI (No.)	Proportion (%)
**Manhiça, Mozambique (Jan–Dec 2011) (Bassat and colleagues, unpublished)**	ALRI	NPA; PCR	114	4.4	96	10.4	413	6.3
**Concepcion, Chile (2012–2013) (Fasce and colleagues, unpublished)**	ALRI	NPA; IFA	216	2.8	85	7.1	464	5
**Pueto Princesa City, Philippines (Aug 2012–Feb 2015) (Oshitani and colleagues, unpublished)**	ALRI	NPS; PCR	236	3.4	166	6	694	5
**Kamalapur, Bangladesh (2013–2014) (Brooks colleagues, unpublished)**	ALRI	NPW; PCR	19	10.5	16	6.2	67	9
**Ciudad de Buenos Aires, Argentina (Jun 2008–Dec 2010) (Echavarria and colleagues, unpublished)**	ARI	NPA; DFA	12	0	18	5.6	73	2.7
**Multiple sites, Philippines (Jul 2000–Dec 2004) (Lucero and colleagues, unpublished)**	ALRI	NPA and NPS; Viral culture	233	5.6	278	10.4	··	··
**Soweto, Gauteng Prov, South Africa (2015–2017) (Nunes and colleagues, unpublished)**	ALRI	NPS; PCR	2461	2.6	1185	4.9	4400	3.4
**Soweto, Gauteng, South Africa (Jan 2000–Dec 2002) (Madhi and colleagues, unpublished)**	ALRI	NPA; PCR	326	6.7	301	11.3	1409	8.9
**Pune, India (May 2009–Apr 2013) (Hirve and colleagues, unpublished)**	ALRI	NPS; PCR	6	33.3	17	11.8	76	19.7
**Amman, Jordan (Mar 2010–Mar 2013) (Khuri-Bulos and colleagues, unpublished)**	ALRI	NS and TS; PCR	1017	9.1	497	12.1	··	··
**Kilifi, Kenya (Jan 2007– Dec 2017) (Nokes and colleagues, unpublished)**	ALRI	NPS; PCR	1347	5.1	760	7.1	3372	5.2
**Buenos Aires, Argentina (May 2011–Aug 2013) (Polack and colleagues, unpublished)**	ALRI	NPA; PCR	1794	9.1	956	15.6	··	··
**Kathmandu and surrouning disctricts, Nepal (Jan 2006–Jan 2008) (Strand and colleagues, unpublished)**	ALRI	NPA; PCR	248	2.4	173	1.2	··	··
**Klerksdorp, South Africa (2010–2015) (Cohen and colleagues, unpublished)**	ALRI	NPA; PCR	504	3.8	269	4.1	1259	4
**Pietermaritzburg, Kwa–Zulu Natal Province, South Africa (2010–2015) (Cohen and colleagues, unpublished)**	ALRI	NPA; PCR	883	4.6	442	6.6	2164	5.1
**Naval, Philippines (Sep 2012–Jul 2016) (Oshitani and colleagues, unpublished)**	ALRI	NPS; PCR	451	2.4	218	2.8	1092	2.9
**Navajo and White Mountain Apache, USA (2009) (Bhat et al, 2013)[28]**	ALRI	NW; PCR	··	··	··	··	··	··

Location (reference)	Case definition	Specimen and test	0–5 m		6–11 m		0–59 m	
			Tested ALRI (No.)	Proportion (%)	Tested ALRI (No.)	Proportion (%)	Tested ALRI (No.)	Proportion (%)
**Nanjing, China (2008-2009) (Chen et al, 2010)** [115]	ALRI	NP specimen; DFA	··	··	··	··	··	··
**Egypt (2007-2014) (Horton et al, 2017)** [72]	ARI	NPS and OPS; PCR	··	··	··	··	3292	9.0
**Jordan (2008-2010) (Horton et al, 2017)** [137]	ARI	NPS and OPS; PCR	··	··	··	··	578	9.2
**Oman (2008-2009) (Horton et al, 2017)**[137]	ARI	NPS and OPS; PCR	··	··	··	··	473	11.6
**Qatar (2008-2009) (Horton et al, 2017)** [137]	ARI	NPS and OPS; PCR	··	··	··	··	15	13.3
**Yemen (2010-2014) (Horton et al, 2017)** [137]	ARI	NPS and OPS; PCR	··	··	··	··	628	1.6
**Shenzhen, China (2007-2010) (He et al, 2014)** [138]	ARI	NPA; PCR	595	3.5	408	2.9	1815	3.3
**Shanghai, China (2016-2017) (Li et al, 2018)** [139]	ARI	NPS or sputum specimens; PCR	··	··	··	··	··	··
**Beijing and Shandong, China (2012-2015) (Yu et al, 2018)** [140]	ARI	respiratory specimens (NPS, NPA, sputum, bronchoalveolar lavage); PCR	··	··	··	··	1206	4.1
**Guangzhou, China (2013-2016) (Zhang et al, 2018)**[141]	ARI	TS; NA	902	1.7	1342	2.2	4399	2.2
**Weifang, China (2016-2017) (蒋华芳 et al, 2018)** [142]	ARI	NP secretion; PCR	218	1.4	203	2.5	··	··
**The Gambia (2012-2013) (Knoll and colleagues, unpublished)**	ALRI	NPS, OPS, induced sputum; PCR	256	4.7	138	11.6	623	8

Table S13.6. Description of included studies reporting hospital admission rates of hMPV–associated ALRI with hypoxaemia (per 1,000 children per year).[*]

Location (reference)	Case Definition	Denominator type	Specimen and test	Hospital admission rates				
				0–5 m	6–11 m	12–23 m	24–59 m	0–59 m
**Nakhon Phanom, Thailand (Jan 2012–Dec 2013) (Knoll and colleagues, unpublished)**	ALRI AND hypoxaemia OR danger signs	Census derived population	NP/OP and induced sputum; PCR	··	··	0	0	0
**Sa Kaeo, Thailand (Jan 2012–Dec 2013) (Knoll and colleagues, unpublished)**	ALRI AND hypoxaemia OR danger signs	Census derived population	NP/OP and induced sputum; PCR	··	··	0	0.1	0.1
**The Gambia (2012-2013) (Knoll and colleagues, unpublished)**	ALRI AND hypoxaemia OR danger signs	Census derived population	NP/OP and induced sputum; PCR	0	0	0	0	0
**Kawayan and Caibiran, Philippines (Feb 2014–Jun 2016) (Oshitani and colleagues, unpublished)**	ALRI AND hypoxaemia OR danger signs	Defined population estimates	NPS; PCR	0	0	1	0	0.2
**Nha Trang city, Viet Nam (Jan 2007–Dec 2014) (Yoshida and colleagues, unpublished)**	ALRI AND hypoxaemia OR danger signs	Census derived population	NPS; PCR	··	··	0.3	0.1	0.2
**Paarl, South Africa (Jun 2012–Dec 2017) (Zar and colleagues, unpublished)**	ALRI AND hypoxaemia OR ICU OR MV	Defined population estimates	NPS; PCR	6	0	0	0	0.8
**Manhiça, Mozambique (Jan–Dec 2011) (Bassat and colleagues, unpublished)**	ALRI AND hypoxaemia OR danger signs	Census derived population	NPA; PCR	0	0.4	0	0.1	0.1
**Kamalapur, Bangladesh (2013–2014) (Brooks colleagues, unpublished)**	ALRI AND hypoxaemia OR danger signs	Defined population estimates	NPW; PCR	0.8	0	0	0	0.3
**Multiple sites, Philippines (Jul 2000–Dec 2004) (Lucero and colleagues, unpublished)**	ALRI AND hypoxaemia OR danger signs	Defined population estimates	NPA and NPS; Viral culture	1.7	2.2	1.3	··	··
**Soweto, Gauteng, South Africa (Jan 2000–Dec 2002) (Madhi and colleagues, unpublished)**	ALRI AND hypoxaemia OR ICU OR danger signs	Defined population estimates	NPA; PCR	2.2	1.1	0.7	0.3	0.6
**Amman, Jordan (Mar 2010–Mar 2013) (Khuri-Bulos and colleagues, unpublished)**	ALRI AND hypoxaemia OR ICU OR MV	Census derived population	NS and TS; PCR	0.3	0.1	0	··	··

[*] ALRI: physician diagnosed acute lower respiratory infections requiring hospital admission. ICU: intensive care unit. MV: mechanical ventilation. NP specimens: nasopharyngeal specimens. OP specimens: oropharyngeal specimens. NS: nasal swab. TS: throat swab. NPA: nasopharyngeal aspirate. NPS: nasopharyngeal swab. PCR: polymerase chain reaction.

Location (reference)	Case Definition	Denominator type	Specimen and test	Hospital admission rates				
				0–5 m	6–11 m	12–23 m	24–59 m	0–59 m
**Kilifi, Kenya (Jan 2007– Dec 2017) (Nokes and colleagues, unpublished)**	ALRI AND hypoxaemia OR ICU OR danger signs	Census derived population	NPS; PCR	1.5	1.3	0.3	0.1	0.4
**Valencia Region, Spain (2014–2017) (Mira Iglesias and colleagues, unpublished)**	All AND hypoxaemia OR ICU OR MV	Census derived population	NPS and NS; PCR	··	··	0	0	0
**Buenos Aires, Argentina (May 2011–Aug 2013) (Polack and colleagues, unpublished)**	ALRI AND hypoxaemia OR ICU OR MV	Census derived population	NPA; PCR	1.1	1	0.2	··	··

Appendix 14. Meta-estimates by narrow age groups

Table S14.1 Incidence rates of hMPV-associated ALRI cases (per 1,000 children per year) in children younger than five years

	0-27 d		1-2 m		3-5 m		6-11 m		12-23 m		2-4 y	
	**No** *	**Rate**†	**No**	**Rate**	**No**	**Rate**	**No**	**Rate**	**No**	**Rate**	**No**	**Rate**
**Developing**	4	1.7 (0.1-40.3)	5	16 (4.2-58.9)	5	28.7 (10.7-74.5)	6	32.8 (16.0-65.9)	6	35.1 (22.9-53.3)‡	4	16.3 (9.3-28.5)‡
**Lower middle income**	3	NA	3	11.9 (1.1-118.4)	3	29.4 (7.9-102.7)	5	30.7 (12.2-75.1)	5	38.4 (24.1-60.6)‡	3	18.2 (9-36.4)‡

* No: number of studies.
† Rate: incidence rate.
‡ Of the included studies, one study reported a much higher incidence rate of hMPV-associated ALRI in the 12-23 month and 24-59 month age groups than in the 0-11 month age group.

Table S14.2 Incidence rates of hMPV-associated severe ALRI cases (per 1,000 children per year) by narrow age groups.

	0-27 d		1-2 m		3-5 m		6-11 m		12-23 m		2-4 y	
	**No***	**Rate†**	**No**	**Rate**	**No**	**Rate**	**No**	**Rate**	**No**	**Rate**	**No**	**Rate**
**Developing**	3	1.9 (0.1-43.7)	4	6.2 (0.4-83.4)	4	8.7 (1.1-67.5)	4	3.3 (0.2-47.5)	4	2.5 (0.3-24.4)	3	5.9 (3.4-10)
**Lower middle income**	2	NA	2	10.2 (0.4-219)	2	10.8 (0.9-121.7)	2	8.2 (1-63.4)	2	2.2 (0-237.3)	2	4.5 (2.5-7.8)

* No: number of studies.
† Rate: incidence rate.

Table S14.3 Hospital admission rates of hMPV-associated ALRI (per 1,000 children per year) by narrow age groups.

	0-27 d		1-2 m		3-5 m		6-11 m		12-23 m		2-4 y	
	**No***	**Rate†**	**No**	**Rate**	**No**	**Rate**	**No**	**Rate**	**No**	**Rate**	**No**	**Rate**
**Developing**	9	0.4 (0.2-0.8)	11	4.0 (2.5-6.4)	11	3.5 (2.4-5.2)	12	2.6 (1.6-4.1)	18	1 (0.6-1.6)	18	0.3 (0.2-0.5)
**Industrialised**	0	NA	0	NA	0	NA	3	2.5 (2.2-2.8)	5	1.6 (1.3-1.9)	4	0.4 (0.4-0.5)
**Low and lower middle**	5	0.3 (0.1-1.2)	5	3.2 (1.5-6.9)	6	2.9 (2.1-3.9)	6	2.8 (1.6-4.9)	7	1.0 (0.6-1.6)	7	0.3 (0.2-0.3)
**Upper middle**	3	0.6 (0.2-1.8)	4	5.2 (2.1-12.7)	4	4.2 (1.9-8.8)	5	2.5 (1-5.9)	9	0.8 (0.5-1.4)	9	0.4 (0.2-0.7)
**High**	1	NA	1	NA	1	NA	4	2.8 (2.2-3.5)	7	1.1 (0.7-1.7)	6	0.3 (0.2-0.5)

* No: number of studies.
† Rate: hospital admission rate.

Table S14.4 Hospital admission rates of hMPV-associated ALRI with hypoxaemia (per 1,000 children per year) by narrow age groups.

	0-27 d		1-2 m		3-5 m		6-11 m		12-23 m		2-4 y	
	No*	Rate†	No	Rate	No	Rate	No	Rate	No	Rate	No	Rate
**Developing**	6	0.1 (0-2.9)	8	1.4 (0.8-2.6)	8	1.5 (1.1-1.9)	9	0.6 (0.3-1.3)	12	0.2 (0.1-0.5)	9	0.1 (0.1-0.2)
**Low and Lower middle**	2	NA	5	2.3 (1.5-3.5)	5	0.9 (0.3-3.3)	5	1.1 (0.6-2.2)	6	0.3 (0.1-0.8)	5	0.1 (0.1-0.1)
**Upper middle**	1	NA	2	1.1 (0.2-7.9)	2	3.1 (1.4-7)	3	0.3 (0.1-1.2)	5	0.1 (0-0.6)	4	0.2 (0.1-0.4)
**High**	1	NA	1	NA	1	NA	1	NA	1	NA	1	NA

* No: number of studies.
† Rate: hospital admission rate.

Table S14.5 hMPV-associated ALRI in-hospital proportion meta-estimates by narrow age groups*

	0-27 d		1-2 m		3-5 m		6-11 m		12-23 m		2-4 y	
	**No †**	**Proportions‡**	**No**	**Proportions**	**No**	**Proportions**	**No**	**Proportions**	**No**	**Proportions**	**No**	**Proportions**
**Developing**	19	1.5 (0.7-3.1)	31	4.8 (3.9-5.8)	34	5.9 (4.8-7.2)	50	6.7 (5.6-8)	39	5.7 (4.6-7)	33	5.2 (4.2-6.4)
**Industrialised**	3	1.6 (1-2.8)	3	3 (1.8-5)	3	7.1 (5.7-8.7)	5	8 (5.5-11.6)	8	8 (5.2-12.1)	4	8.2 (3.3-18.8)
**Low**	1	NA	4	4.7 (3-7.4)	4	4 (2.6-6.1)	4	6.7 (3-14.1)	4	5.5 (2.4-12.3)	3	6.9 (4.1-11.5)
**Lower middle**	9	1.4 (0.6-3.1)	16	5 (4-6.2)	17	6.3 (4.6-8.5)	17	6.6 (4.9-8.9)	16	5.8 (4.5-7.4)	16	5.6 (4.2-7.6)
**Upper middle**	6	2.4 (0.7-7.6)	9	4.5 (3.2-6.3)	10	5.7 (4-8)	26	6.5 (5.1-8.3)	16	5.3 (3.6-7.7)	12	4.5 (3.2-6.2)
**High**	6	1.6 (0.9-2.6)	5	3.2 (1.6-6.3)	6	7.5 (5.7-9.9)	8	8.8 (6.2-12.3)	11	7.7 (5.4-10.8)	6	7.4 (3.7-14.3)

* Based on all available in-hospital proportion data.
† No: number of studies.
‡ Rate: hospital admission rate.

Appendix 15. Checklist of information that should be included in new reports of global health estimates

Item #	Checklist item	Reported on page #
**Objectives and funding**		
**1**	Define the indicator(s), populations (including age, sex, and geographic entities), and time period(s) for which estimates were made.	9-10
**2**	List the funding sources for the work.	Summary
**Data Inputs**		
For all data inputs from multiple sources that are synthesized as part of the study:		
**3**	Describe how the data were identified and how the data were accessed.	9-10
**4**	Specify the inclusion and exclusion criteria. Identify all ad-hoc exclusions.	10
**5**	Provide information on all included data sources and their main characteristics. For each data source used, report reference information or contact name/institution, population represented, data collection method, year(s) of data collection, sex and age range, diagnostic criteria or measurement method, and sample size, as relevant.	Supplementary material 6-7; 36-53.
**6**	Identify and describe any categories of input data that have potentially important biases (e.g., based on characteristics listed in item 5).	17; Supplementary material 27-33.
For data inputs that contribute to the analysis but were not synthesized as part of the study:		
**7**	Describe and give sources for any other data inputs.	Supplementary material 10; 15-16; 18-19; 36-53
For all data inputs:		
**8**	Provide all data inputs in a file format from which data can be efficiently extracted (e.g., a spreadsheet rather than a PDF), including all relevant meta-data listed in item 5. For any data inputs that cannot be shared because of ethical or legal reasons, such as third-party ownership, provide a contact name or the name of the institution that retains the right to the data.	Data have been presented in the supplementary material. Data will be made available on Edinburgh Datashare (https://datashare.is.ed.ac.uk/) later.
**Data analysis**		
**9**	Provide a conceptual overview of the data analysis method. A diagram may be helpful.	Supplementary material 15
**10**	Provide a detailed description of all steps of the analysis, including mathematical formulae. This description should cover, as relevant, data cleaning, data pre-processing, data adjustments and weighting of data sources, and mathematical or statistical model(s).	10-13; Supplementary material 15-19; 21; 23
**11**	Describe how candidate models were evaluated and how the final model (s) were selected.	12-13; Supplementary material 8-19; 22-23.
**12**	Provide the results of an evaluation of model performance, if done, as well as the results of any relevant sensitivity analysis.	Supplementary material 8-19; 22-23.
**13**	Describe methods for calculating uncertainty of the estimates. State which sources of uncertainty were, and were not, accounted for in the uncertainty analysis.	11
**14**	State how analytic or statistical source code used to generate estimates can be accessed.	Major code used in this study will be made available upon request.
**Results and Discussion**		
**15**	Provide published estimates in a file format from which data can be efficiently extracted.	Estimates can be easily extracted in main table and supplementary table. Main tables will be provided on Edinburgh Datashare (https://datashare.is.ed.ac.uk/) later.
**16**	Report a quantitative measure of the uncertainty of the estimates (e.g. uncertainty intervals).	13-15
**17**	Interpret results in light of existing evidence. If updating a previous set of estimates, describe the reasons for changes in estimates.	16-17
**18**	Discuss limitations of the estimates. Include a discussion of any modelling assumptions or data limitations that affect interpretation of the estimates.	16-17

References

1. Troeger C, Blacker B, Khalil IA, et al. Estimates of the global, regional, and national morbidity, mortality, and aetiologies of lower respiratory infections in 195 countries, 1990–2016: a systematic analysis for the Global Burden of Disease Study 2016. *The Lancet Infectious Diseases* 2018; **18**(11): 1191-210.
2. McAllister DA, Liu L, Shi T, et al. Global, regional, and national estimates of pneumonia morbidity and mortality in children younger than 5 years between 2000 and 2015: a systematic analysis. *The Lancet Global Health* 2018.
3. United Nations, Department of Economic and Social Affairs, Population Division. World Population Prospects: The 2017 Revision. 2017.
4. Wang X, Li Y, O'Brien KL, et al. Global burden of respiratory infections associated with seasonal influenza in children under 5 years in 2018: a systematic review and modelling study. *The Lancet Global Health*.
5. UNICEF. Pneumonia care-seeking 2016. https://data.unicef.org/resources/pneumonia-care-seeking-interactive-dashboard/ (accessed 1 Oct 2019).
6. Bennett A, Eisele T, Keating J, Yukich J. Global Trends in Care Seeking and Access to Diagnosis and Treatment of Childhood Illnesses. Rockville, Maryland, USA: ICF International, 2015.
7. Najnin N, Bennett CM, Luby SP. Inequalities in care-seeking for febrile illness of under-five children in urban Dhaka, Bangladesh. *Journal of health, population, and nutrition* 2011; **29**(5): 523-31.
8. Onyango D, Kikuvi G, Amukoye E, Omolo J. Risk factors of severe pneumonia among children aged 2-59 months in western Kenya: a case control study. *The Pan African medical journal* 2012; **13**: 45.
9. Centers for Disease Control and Prevention, National Center for Health Statistics. Underlying Cause of Death 1999-2017 on CDC WONDER Online Database. December 2018 http://wonder.cdc.gov/ucd-icd10.html (accessed 23 November 2019).
10. Ferdous F, Ahmed S, Das SK, et al. Pneumonia mortality and healthcare utilization in young children in rural Bangladesh: a prospective verbal autopsy study. *Tropical Medicine and Health* 2018; **46**(1): 17.
11. Ahmed M, Aleem MA, Roguski K, et al. Estimates of seasonal influenza-associated mortality in Bangladesh, 2010-2012. *Influenza Other Respir Viruses* 2018; **12**(1): 65-71.
12. WHO. Causes of child mortality, 2017. Dec 2018 2018. https://www.who.int/gho/child_health/mortality/causes/en/ (accessed 20 Sep 2019).
13. Causes of severe pneumonia requiring hospital admission in children without HIV infection from Africa and Asia: the PERCH multi-country case-control study. *Lancet (London, England)* 2019.
14. Benet T, Sanchez Picot V, Messaoudi M, et al. Microorganisms Associated With Pneumonia in Children <5 Years of Age in Developing and Emerging Countries: The GABRIEL Pneumonia Multicenter, Prospective, Case-Control Study. *Clinical infectious diseases : an official publication of the Infectious Diseases Society of America* 2017; **65**(4): 604-12.
15. Shi T, McLean K, Campbell H, Nair H. Aetiological role of common respiratory viruses in acute lower respiratory infections in children under five years: A systematic review and meta–analysis. *Journal of global health* 2015; **5**(1).
16. Shi T, Balsells E, Wastnedge E, et al. Risk factors for respiratory syncytial virus associated with acute lower respiratory infection in children under five years: Systematic review and meta-analysis. *J Glob Health* 2015; **5**(2): 020416.
17. Pneumonia Etiology Research for Child Health Study Group (PERCH). Causes of severe pneumonia requiring hospital admission in children without HIV infection from Africa and Asia: the PERCH multi-country case-control study. *Lancet (London, England)* 2019; **394**(10200): 757-79.

18. CHAMPS. Child Health and Mortality Prevention Surveillance. https://champshealth.org/ (accessed 6 Oct 2019).
19. Blau DM, Caneer JP, Philipsborn RP, et al. Overview and Development of the Child Health and Mortality Prevention Surveillance Determination of Cause of Death (DeCoDe) Process and DeCoDe Diagnosis Standards. *Clinical Infectious Diseases* 2019; **69**(Supplement_4): S333-S41.
20. Salzberg NT, Sivalogan K, Bassat Q, et al. Mortality Surveillance Methods to Identify and Characterize Deaths in Child Health and Mortality Prevention Surveillance Network Sites. *Clinical infectious diseases : an official publication of the Infectious Diseases Society of America* 2019; **69**(Supplement_4): S262-s73.
21. Sterne JA, White IR, Carlin JB, et al. Multiple imputation for missing data in epidemiological and clinical research: potential and pitfalls. *Bmj* 2009; **338**: b2393.
22. Rubin DB. Multiple imputation for nonresponse in surveys. New York: Wiley; 1987.
23. Honaker J, King G, Blackwell M. Amelia II: A Program for Missing Data. *J Stat Softw* 2011; **45**(7): 47.
24. Wu A, Budge PJ, Williams J, et al. Incidence and risk factors for respiratory syncytial virus and human metapneumovirus infections among children in the remote highlands of Peru. *PloS one* 2015; **10**(6): e0130233.
25. Williams JV, Harris PA, Tollefson SJ, et al. Human metapneumovirus and lower respiratory tract disease in otherwise healthy infants and children. *New England Journal of Medicine* 2004; **350**(5): 443-50.
26. Sarna M, Lambert SB, Sloots TP, et al. Viruses causing lower respiratory symptoms in young children: findings from the ORChID birth cohort. *Thorax* 2018; **73**(10): 969-79.
27. Kusel MM, de Klerk NH, Holt PG, Kebadze T, Johnston SL, Sly PD. Role of respiratory viruses in acute upper and lower respiratory tract illness in the first year of life: a birth cohort study. *Pediatr Infect Dis J* 2006; **25**(8): 680-6.
28. Bhat N, Tokarz R, Jain K, et al. A prospective study of agents associated with acute respiratory infection among young American Indian children. *Pediatr Infect Dis J* 2013; **32**(8): e324-33.
29. Havers FP, Fry AM, Goswami D, et al. Population-based Incidence of Childhood Pneumonia Associated With Viral Infections in Bangladesh. *Pediatr Infect Dis J* 2019; **38**(4): 344-50.
30. Ali A, Akhund T, Warraich GJ, et al. Respiratory Viruses Associated With Severe Pneumonia in Children Under 2 Years Old in a Rural Community in Pakistan. *Journal of Medical Virology* 2016; **88**(11): 1882-90.
31. Edwards KM, Zhu Y, Griffin MR, et al. Burden of human metapneumovirus infection in young children. *The New England journal of medicine* 2013; **368**(7): 633-43.
32. Groome MJ, Moyes J, Cohen C, et al. Human metapneumovirus-associated severe acute respiratory illness hospitalisation in HIV-infected and HIV-uninfected South African children and adults. *Journal of Clinical Virology* 2015; **69**: 125-32.
33. Hasan R, Rhodes J, Thamthitiwat S, et al. Incidence and etiology of acute lower respiratory tract infections in hospitalized children younger than 5 years in rural Thailand. *Pediatr Infect Dis J* 2014; **33**(2): e45-52.
34. Jain S, Williams DJ, Arnold SR, et al. Community-acquired pneumonia requiring hospitalization among U.S. children. *New England Journal of Medicine* 2015; **372**(9): 835-45.
35. Mullins JA, Erdman DD, Weinberg GA, et al. Human metapneumovirus infection among children hospitalized with acute respiratory illness. *Emerging infectious diseases* 2004; **10**(4): 700.
36. Nicholson KG, McNally T, Silverman M, Simons P, Stockton JD, Zambon MC. Rates of hospitalisation for influenza, respiratory syncytial virus and human metapneumovirus among infants and young children. *Vaccine* 2006; **24**(1): 102-8.
37. Davis CR, Stockmann C, Pavia AT, et al. Incidence, Morbidity, and Costs of Human Metapneumovirus Infection in Hospitalized Children. *Journal of the Pediatric Infectious Diseases Society* 2016; **5**(3): 303-11.

38. Broor S, Dawood FS, Pandey BG, et al. Rates of respiratory virus-associated hospitalization in children aged <5 years in rural northern India. *Journal of Infection* 2014; **68**(3): 281-9.

39. McCracken JP, Arvelo W, Ortiz J, et al. Comparative epidemiology of human metapneumovirus- and respiratory syncytial virus-associated hospitalizations in Guatemala. *Influenza & Other Respiratory Viruses* 2014; **8**(4): 414-21.

40. Ahmed JA, Katz MA, Auko E, et al. Epidemiology of respiratory viral infections in two long-term refugee camps in Kenya, 2007-2010. *Bmc Infectious Diseases* 2012; **12**.

41. Cilla G, Onate E, Perez-Yarza EG, Montes M, Vicente D, Perez-Trallero E. Hospitalization rates for human metapneumovirus infection among 0- to 3-year-olds in Gipuzkoa (Basque Country), Spain. *Epidemiology and infection* 2009; **137**(1): 66-72.

42. Williams JV, Harris PA, Tollefson SJ, et al. Human metapneumovirus and lower respiratory tract disease in otherwise healthy infants and children. *New England Journal of Medicine* 2004; **350**(5): 443-50.

43. Olabarrieta I, Gonzalez-Carrasco E, Calvo C, Pozo F, Casas I, Garcia-Garcia ML. Hospital admission due to respiratory viral infections in moderate preterm, late preterm and term infants during their first year of life. *Allergologia et Immunopathologia* 2015; **43**(5): 469-73.

44. Moe N, Krokstad S, Stenseng IH, et al. Comparing Human Metapneumovirus and Respiratory Syncytial Virus: Viral Co-Detections, Genotypes and Risk Factors for Severe Disease. *PLoS One* 2017; **12**(1): e0170200.

45. O'Callaghan-Gordo C, Bassat Q, Morais L, et al. Etiology and epidemiology of viral pneumonia among hospitalized children in rural Mozambique: a malaria endemic area with high prevalence of human immunodeficiency virus. *Pediatr Infect Dis J* 2011; **30**(1): 39-44.

46. Feikin DR, Njenga MK, Bigogo G, et al. Viral and bacterial causes of severe acute respiratory illness among children aged less than 5 years in a high malaria prevalence area of western Kenya, 2007-2010. *Pediatr Infect Dis J* 2013; **32**(1): e14-9.

47. Chung JY, Han TH, Kim BE, Kim CK, Kim SW, Hwang E-S. Human metapneumovirus infection in hospitalized children with acute respiratory disease in Korea. *J Korean Med Sci* 2006; **21**(5): 838-42.

48. Dollner H, Risnes K, Radtke A, Nordbo SA. Outbreak of human metapneumovirus infection in norwegian children. *Pediatric Infectious Disease Journal* 2004; **23**(5): 436-40.

49. García-García ML, Calvo C, Martín F, Pérez-Breña P, Acosta B, Casas I. Human metapneumovirus infections in hospitalised infants in Spain. *Archives of Disease in Childhood* 2006; **91**(4): 290-5.

50. Gray GC, Capuano AW, Setterquist SF, et al. Multi-year study of human metapneumovirus infection at a large US Midwestern Medical Referral Center. *Journal of clinical virology : the official publication of the Pan American Society for Clinical Virology* 2006; **37**(4): 269-76.

51. Hahn A, Wang W, Jaggi P, et al. Human metapneumovirus infections are associated with severe morbidity in hospitalized children of all ages. *Epidemiology and infection* 2013; **141**(10): 2213-23.

52. Singleton RJ, Bulkow LR, Miernyk K, et al. Viral respiratory infections in hospitalized and community control children in Alaska. *Journal of Medical Virology* 2010; **82**(7): 1282-90.

53. Williams JV, Edwards KM, Weinberg GA, et al. Population-based incidence of human metapneumovirus infection among hospitalized children. *The Journal of infectious diseases* 2010; **201**(12): 1890-8.

54. Takao S, Shimozono H, Kashiwa H, et al. Clinical study of pediatric cases of acute respiratory diseases associated with human metapneumovirus in Japan. *Jpn J Infect Dis* 2003; **56**(3): 127-9.

55. Bashir U, Nisar N, Arshad Y, et al. Respiratory syncytial virus and influenza are the key viral pathogens in children <2 years hospitalized with bronchiolitis and pneumonia in Islamabad Pakistan. *Archives of Virology* 2017; **162**(3): 763-73.

56. Broor S, Dawood FS, Pandey BG, et al. Rates of respiratory virus-associated hospitalization in children aged <5 years in rural northern India. *Journal of Infection* 2014; **68**(3): 281-9.

57. Durigon GS, Oliveira DBL, Felicio MCC, et al. Poor outcome of acute respiratory infection in young children with underlying health condition in Brazil. *International journal of infectious diseases : IJID : official publication of the International Society for Infectious Diseases* 2015; **34**: 3-7.
58. Guerrier G, Goyet S, Chheng ET, et al. Acute viral lower respiratory tract infections in Cambodian children: clinical and epidemiologic characteristics. *Pediatr Infect Dis J* 2013; **32**(1): e8-13.
59. Ijpma FFA, Beekhuis D, Cotton MF, et al. Human metapneumovirus infection in hospital referred South African children. *J Med Virol* 2004; **73**(3): 486-93.
60. Teeratakulpisarn J, Ekalaksananan T, Pientong C, Limwattananon C. Human metapneumovirus and respiratory syncytial virus detection in young children with acute bronchiolitis. *Asian Pacific Journal of Allergy & Immunology* 2007; **25**(2-3): 139-45.
61. Al Hajjar S, Al Thawadi S, Al Seraihi A, Al Muhsen S, Imambaccus H. Human metapneumovirus and human coronavirus infection and pathogenicity in Saudi children hospitalized with acute respiratory illness. *Annals of Saudi Medicine* 2011; **31**(5): 523-7.
62. Azkur D, Özaydin E, Dibek-Misirlioglu E, et al. Viral etiology in infants hospitalized for acute bronchiolitis. *Turk J Pediatr* 2014; **56**(6): 592-6.
63. Benet T, Sylla M, Messaoudi M, et al. Etiology and Factors Associated with Pneumonia in Children under 5 Years of Age in Mali: A Prospective Case-Control Study. *PLoS ONE [Electronic Resource]* 2015; **10**(12): e0145447.
64. Cohen C, Moyes J, Tempia S, et al. Epidemiology of Acute Lower Respiratory Tract Infection in HIV-Exposed Uninfected Infants. *Pediatrics* 2016; **137**(4).
65. Do AHL, van Doorn HR, Nghiem MN, et al. Viral etiologies of acute respiratory infections among hospitalized Vietnamese children in Ho Chi Minh City, 2004–2008. *PloS one* 2011; **6**(3): e18176.
66. Hatipoglu N, Somer A, Badur S, et al. Viral etiology in hospitalized children with acute lower respiratory tract infection. *Turkish Journal of Pediatrics* 2011; **53**(5): 508-16.
67. Kaplan NM, Dove W, Abu-Zeid AF, Shamoon HE, Abd-Eldayem SA, Hart CA. Evidence of human metapneumovirus infection in Jordanian children. *Saudi Med J* 2006; **27**(7): 1081-3.
68. Lozano C J, Yáñez P L, Budnik O I, et al. Infección por metapneumovirus humano en niños hospitalizados por una enfermedad respiratoria aguda grave: descripción clínico- epidemiológica. *Rev chil enferm respir* 2009; **25**(4): 211-7.
69. Pérez MG, Viale D, Parra A, et al. Infección por metapneumovirus en pacientes internados en un hospital pediátrico. *Med infant* 2012; **19**(3): 199-201.
70. Wang YQ, Ji W, Chen ZR, et al. [Prevalence and clinical features of human metapneumovirus infection in hospitalized pediatric patients with respiratory tract infection in Suzhou area]. [Chinese]. *Zhonghua er ke za zhi* 2009; **Chinese journal of pediatrics. 47**(8): 617-20.
71. Brini I, Guerrero A, Hannachi N, et al. Epidemiology and clinical profile of pathogens responsible for the hospitalization of children in Sousse area, Tunisia. *PLOS ONE* 2017; **12**(11): e0188325.
72. Horton KC, Dueger EL, Kandeel A, et al. Viral etiology, seasonality and severity of hospitalized patients with severe acute respiratory infections in the Eastern Mediterranean Region, 2007–2014. *PLOS ONE* 2017; **12**(7): e0180954.
73. Bezerra PGM, Britto MCA, Correia JB, et al. Viral and atypical bacterial detection in acute respiratory infection in children under five years. *PloS one* 2011; **6**(4): e18928-e.
74. 林创兴, 陆学东, 林广裕, et al. 粤东地区喘息性疾病患儿中人偏肺病毒的检出与病原学初步研究. *中华哮喘杂志(电子版)* 2009; **28**(1): 5-7.
75. 梁沫, 张兵, 黄寒, et al. 长沙地区急性下呼吸道感染儿童呼吸道合胞病毒、偏肺病毒临床特征及流行状况分析. *实用预防医学* 2012; **19**(7).
76. 邱秀娟. 2009-2012 年苏州地区住院儿童人偏肺病毒感染临床研究 [硕士]: 苏州大学; 2015.
77. 骆亚丽. 儿童呼吸道人类偏肺病毒感染临床流行特征及免疫发病机制研究; 2009.

78. 沈军. 人偏肺病毒及多种病原体致儿童急性下呼吸道感染的临床及分子流行病学特征 [博士]: 复旦大学; 2009.
79. Foulongne V, Guyon G, Rodiere M, Segondy M. Human metapneumovirus infection in young children hospitalized with respiratory tract disease. *Pediatric Infectious Disease Journal* 2006; **25**(4): 354-9.
80. Hopkins MJ, Redmond C, Shaw JM, et al. Detection and characterisation of human metapneumovirus from children with acute respiratory symptoms in north-west England, UK. *Journal of Clinical Virology* 2008; **42**(3): 273-9.
81. Huguenin A, Moutte L, Renois F, et al. Broad respiratory virus detection in infants hospitalized for bronchiolitis by use of a multiplex RT-PCR DNA microarray system. *Journal of Medical Virology* 2012; **84**(6): 979-85.
82. Maggi F, Pifferi M, Vatteroni M, et al. Human metapneumovirus associated with respiratory tract infections in a 3-year study of nasal swabs from infants in Italy. *Journal of Clinical Microbiology* 2003; **41**(7): 2987-91.
83. Wolf DG, Greenberg D, Shemer-Avni Y, Givon-Lavi N, Bar-Ziv J, Dagan R. Association of human metapneumovirus with radiologically diagnosed community-acquired alveolar pneumonia in young children. *Journal of Pediatrics* 2010; **156**(1): 115-20.
84. Xepapadaki P, Psarras S, Bossios A, et al. Human Metapneumovirus as a causative agent of acute bronchiolitis in infants. *Journal of clinical virology : the official publication of the Pan American Society for Clinical Virology* 2004; **30**(3): 267-70.
85. Zappa A, Canuti M, Frati E, et al. Co- circulation of genetically distinct human metapneumovirus and human bocavirus strains in young children with respiratory tract infections in Italy. *Journal of Medical Virology* 2011; **83**(1): 156-64.
86. Eem YJ, Bae EY, Lee JH, Jeong DC. Risk factors associated with respiratory virus detection in infants younger than 90 days of age. [Korean]. *Korean Journal of Pediatric Infectious Diseases* 2014; **21**(1): 22-8.
87. Hamada H, Ogura A, Hotta C, Wakui T, Ogawa T, Terai M. [Epidemiological study of respiratory viruses detected in patients under two years old who required admission because of lower respiratory disease]. *Kansenshogaku Zasshi* 2014; **88**(4): 423-9.
88. Moore HC, de Klerk N, Keil AD, et al. Use of data linkage to investigate the aetiology of acute lower respiratory infection hospitalisations in children. *Journal of Paediatrics & Child Health* 2012; **48**(6): 520-8.
89. Calvo C, Pozo F, Garcia-Garcia ML, et al. Detection of new respiratory viruses in hospitalized infants with bronchiolitis: a three-year prospective study. *Acta Paediatrica* 2010; **99**(6): 883-7.
90. Pancer KW, Gut W, Abramczuk E, Lipka B, Litwinska B. Non-influenza viruses in acute respiratory infections among young children. High prevalence of HMPV during the H1N1V.2009 pandemic in Poland. *Przeglad Epidemiologiczny* 2014; **68**(4): 627-32.
91. Rodriguez PE, Adamo MP, Paglini MG, Moreno L, Camara JA, Camara A. [Monoinfection of human Metapneumovirus in Cordoba: first clinical and epidemiological research in children with respiratory infection in 2011]. *Revista de la Facultad de Ciencias Medicas de Cordoba* 2016; **73**(3): 170-5.
92. Canducci F, Debiaggi M, Sampaolo M, et al. Two-year prospective study of single infections and co-infections by respiratory syncytial virus and viruses identified recently in infants with acute respiratory disease. *Journal of Medical Virology* 2008; **80**(4): 716-23.
93. Cebey-López M, Herberg J, Pardo-Seco J, et al. Viral Co-Infections in Pediatric Patients Hospitalized with Lower Tract Acute Respiratory Infections. *PLoS One* 2015; **10**(9): e0136526-e.
94. Richter J, Panayiotou C, Tryfonos C, et al. Aetiology of Acute Respiratory Tract Infections in Hospitalised Children in Cyprus. *PLOS ONE* 2016; **11**(1): e0147041.
95. Gurgel RQ, Bezerra PG, Duarte Mdo C, et al. Relative frequency, Possible Risk Factors, Viral Codetection Rates, and Seasonality of Respiratory Syncytial Virus Among Children With Lower Respiratory Tract Infection in Northeastern Brazil. *Medicine* 2016; **95**(15): e3090.

96. Jain B, Singh AK, Dangi T, et al. High prevalence of human metapneumovirus subtype B in cases presenting as severe acute respiratory illness: an experience at tertiary care hospital. *The clinical respiratory journal* 2014; **8**(2): 225-33.
97. Kenmoe S, Tchendjou P, Vernet MA, et al. Viral etiology of severe acute respiratory infections in hospitalized children in Cameroon, 2011-2013. *Influenza and other Respiratory Viruses* 2016; **10**(5): 386-93.
98. Khamis FA, Al-Kobaisi MF, Al-Areimi WS, Al-Kindi H, Al-Zakwani I. Epidemiology of respiratory virus infections among infants and young children admitted to hospital in Oman. *Journal of Medical Virology* 2012; **84**(8): 1323-9.
99. Liu T, Li Z, Zhang S, et al. Viral Etiology of acute respiratory tract infections in hospitalized children and adults in Shandong Province, China. *Virol J* 2015; **12**: 168-.
100. Lu G, Li J, Xie Z, et al. Human metapneumovirus associated with community-acquired pneumonia in children in Beijing, China. *Journal of Medical Virology* 2013; **85**(1): 138-43.
101. Oliveira DB, Durigon EL, Carvalho AC, et al. Epidemiology and genetic variability of human metapneumovirus during a 4-year-long study in Southeastern Brazil. *Journal of Medical Virology* 2009; **81**(5): 915-21.
102. Wansaula Z, Olsen SJ, Casal MG, et al. Surveillance for severe acute respiratory infections in Southern Arizona, 2010-2014. *Influenza and other Respiratory Viruses* 2016; **10**(3): 161-9.
103. Yan XL, Li YN, Tang YJ, et al. Clinical Characteristics and Viral Load of Respiratory Syncytial Virus and Human Metapneumovirus in Children Hospitaled for Acute Lower Respiratory Tract Infection. *Journal of Medical Virology* 2017; **89**(4): 589-97.
104. Do LAH, Bryant JE, Tran AT, et al. Respiratory syncytial virus and other viral infections among children under two years old in southern Vietnam 2009-2010: Clinical characteristics and disease severity. *PLoS ONE* 2016; **11 (8) (no pagination)**(e0160606).
105. Huang G, Yu D, Mao N, et al. Viral Etiology of Acute Respiratory Infection in Gansu Province, China, 2011. *PLoS ONE* 2013; **8 (5) (no pagination)**(e64254).
106. Liao X, Hu Z, Liu W, et al. New Epidemiological and Clinical Signatures of 18 Pathogens from Respiratory Tract Infections Based on a 5-Year Study. *PLoS ONE [Electronic Resource]* 2015; **10**(9): e0138684.
107. Liu C-Y, Xiao Y, Xie Z-d, et al. [Viral etiology of acute respiratory tract infection among pediatric inpatients and outpatients from 2010 to 2012 in Beijing, China]. *Zhonghua Er Ke Za Zhi* 2013; **51**(4): 255-9.
108. Mazumdar J, Chawla-Sarkar M, Rajendran K, et al. Burden of respiratory tract infections among paediatric in and out-patient units during 2010-11. *European Review for Medical & Pharmacological Sciences* 2013; **17**(6): 802-8.
109. Ou S-Y, Lin G-Y, Wu Y, Lu X-D, Lin C-X, Zhou R-B. [Viral pathogens of acute lower respiratory tract infection in hospitalized children from East Guangdong of China]. *Zhongguo Dang Dai Er Ke Za Zhi* 2009; **11**(3): 203-6.
110. Wang TL, Zheng GM, Jiang ZY, Tang LF, Tang HF, Chen ZM. Human Metapneumovirus Infection in Hospitalised Children with Acute Lower Respiratory Tract Infection in Hangzhou, China. *Hong Kong Journal of Paediatrics* 2013; **18**(1): 6-11.
111. Xiao N-G, Zhang B, Duan Z-J, et al. [Viral etiology of 1165 hospitalized children with acute lower respiratory tract infection]. *Zhongguo Dang Dai Er Ke Za Zhi* 2012; **14**(1): 28-32.
112. Zhang TG, Li AH, Lyu M, Chen M, Huang F, Wu J. Detection of respiratory viral and bacterial pathogens causing pediatric community-acquired pneumonia in Beijing using real-time PCR. *Chronic Diseases and Translational Medicine* 2015; **1**(2): 110-6.
113. Bhuyan GS, Hossain MA, Sarker SK, et al. Bacterial and viral pathogen spectra of acute respiratory infections in under-5 children in hospital settings in Dhaka city. *PLoS ONE* 2017; **12 (3) (no pagination)**(e0174488).

114. TAG A. Detection of Human Metapneumovirus in Hospitalized Children with Acute Respiratory Tract Infections in Sulaimani Province, Iraq. *Medical Microbiology & Diagnosis* 2015; **4**(2).
115. Chen X, Zhang ZY, Zhao Y, Liu EM, Zhao XD. Acute lower respiratory tract infections by human metapneumovirus in children in Southwest China: a 2-year study. *Pediatric Pulmonology* 2010; **45**(8): 824-31.
116. Smuts H. Human coronavirus NL63 infections in infants hospitalised with acute respiratory tract infections in South Africa. *Influenza & Other Respiratory Viruses* 2008; **2**(4): 135-8.
117. Xu L, He X, Zhang D-m, et al. Surveillance and genome analysis of human bocavirus in patients with respiratory infection in Guangzhou, China. *PLoS One* 2012; **7**(9): e44876-e.
118. 彭颖. 2012-2013 年长沙地区急性下呼吸道感染住院儿童病毒谱流行学调查 [硕士]: 湖南师范大学; 2014.
119. 杜丽娜. 重庆地区儿童呼吸道合胞病毒和偏肺病毒分子流行病学研究 [硕士]: 重庆医科大学; 2010.
120. 何杨. 2011 ~2012 年某院门诊及住院患儿呼吸道病毒感染特征分析. *中国妇幼保健* 2015; **30**(3): 382-4.
121. 盛曙君. 呼吸道感染患儿人偏肺病毒的感染情况. *浙江预防医学* 2013; (01).
122. 魏美晨. 住院喘息患儿偏肺病毒及呼吸道合胞病毒感染的研究 [硕士]: 首都医科大学; 2013.
123. 吴琼, 陈礼娟, 黄新泉, 欧书腾, 刘子菁, 范楚平. 郴州地区 5 岁以下住院儿童严重急性呼吸道感染病毒病原学研究基金项目:郴州市科技局资助重点项目(CZ2013065);郴州市第一人民医院重点项目(N2013-005).通讯作者:范楚平. *医学理论与实践* 2017; (7): 943-5,51.
124. 张海邻, 陈小芳, 吕芳芳, et al. 多重 PCR 技术检测儿童下呼吸道感染病毒和不典型病原体的价值. *温州医科大学学报* 2017; (11): 791-5,800.
125. 蔡勇, 陈德晖, 刘文宽, 王群, 陈晓雯, 周荣. 广州地区急性呼吸道感染住院儿童病毒病原谱. *中国医学创新* 2017; (21): 19-22.
126. 张俊华, 柏学民, 金颖, 叶青, 雷晓平, 李磊. 银川地区儿童偏肺病毒感染状况的研究. *宁夏医学杂志* 2013; **35**(7): 612-3.
127. 于德山, 任丽丽, 陈建华, et al. 白银市 5 岁以下急性下呼吸道感染住院儿童病原学分析. *中国病毒病杂志* 2017; **7**(05): 360-5.
128. 赵辛. 急性下呼吸道感染住院儿童病毒谱调查及人副流感病毒 1-4 型的现状分析 [硕士]: 湖南师范大学; 2012.
129. 刘沁, 张兵, 谢志萍, et al. 长沙地区急性下呼吸道感染住院儿童的病毒病原学分析. *湖南师范大学学报（医学版）* 2015; (1): 26-31.
130. 卢庆彬. 儿童急性呼吸道感染病毒流行特征与基因特征研究; 2013.
131. 寇宇, 于新芬, 李钧, et al. 儿童呼吸道感染标本人偏肺病毒的分子检测与分型. *中华实验和临床病毒学杂志* 2016; **30**(2): 161-5.
132. 季伟, 王宇清, 陈正荣, 邵雪军, 季正华, 徐俊. 2006-2008 年苏州地区儿童呼吸道人偏肺病毒感染的流行和临床特征. *临床儿科杂志* 2010; **28**(12): 1155-8.
133. Wen S, Lv F, Chen X, et al. Application of a nucleic acid-based multiplex kit to identify viral and atypical bacterial aetiology of lower respiratory tract infection in hospitalized children. *J Med Microbiol* 2019; **68**(8): 1211-8.
134. Zhao Y, Lu R, Shen J, Xie Z, Liu G, Tan W. Comparison of viral and epidemiological profiles of hospitalized children with severe acute respiratory infection in Beijing and Shanghai, China. *BMC Infectious Diseases* 2019; **19**(1): 729.
135. 任吟莹, 黄莉, 王美娟, et al. 儿童呼吸道人副流感病毒感染临床特征及流行病学特点. *中华实用儿科临床杂志* 2017; (4): 270-4.

136. 孙志豪, 张荣华, 钟超珍. 东莞市 2017-2018 年儿童下呼吸道感染病例监测结果. *分子诊断与治疗杂志* 2019; **11**(4): 329-37.
137. Horton KC, Dueger EL, Kandeel A, et al. Viral etiology, seasonality and severity of hospitalized patients with severe acute respiratory infections in the Eastern Mediterranean Region, 2007-2014. *PloS one* 2017; **12**(7): e0180954-e.
138. He Y, Lin G-Y, Wang Q, et al. A 3-year prospective study of the epidemiology of acute respiratory viral infections in hospitalized children in Shenzhen, China. *Influenza and Other Respiratory Viruses* 2014; **8**(4): 443-51.
139. Li J, Tao Y, Tang M, et al. Rapid detection of respiratory organisms with the FilmArray respiratory panel in a large children's hospital in China. *BMC Infectious Diseases* 2018; **18 (1) (no pagination)**(510).
140. Yu J, Xie Z, Zhang T, et al. Comparison of the prevalence of respiratory viruses in patients with acute respiratory infections at different hospital settings in North China, 2012-2015. *BMC Infectious Diseases* 2018; **18**(1): 72.
141. Zhang L, Liu W, Liu D, et al. Epidemiological and clinical features of human metapneumovirus in hospitalised paediatric patients with acute respiratory illness: a cross-sectional study in Southern China, from 2013 to 2016. *BMJ Open* 2018; **8**(2): e019308.
142. 蒋华芳, 李锦亮, 李丽, 王媛媛, 韩文宁. 潍坊地区儿童急性呼吸道感染病毒病原学分析. *中华实验和临床病毒学杂志* 2018; **32**(3): 272-6.
